# Explore the Potential Ingredients for Detoxification of Honey-Fired Licorice (ZGC) Based on the Metabolic Profile by UPLC-Q-TOF-MS

**DOI:** 10.3389/fchem.2022.924685

**Published:** 2022-07-13

**Authors:** Yinjie Wang, Yu Ning, Ting He, Yingtong Chen, Wenhui Han, Yinping Yang, Cui-Xian Zhang

**Affiliations:** ^1^ School of Pharmaceutical Sciences, Guangzhou University of Chinese Medicine, Guangzhou, China; ^2^ Ningxia Chinese Medicine Research Center, Yinchuan, China; ^3^ Ningxia Hui Medicine Research Institute, Yinchuan, China

**Keywords:** honey-fired licorice, UPLC-Q-TOF-MS, metabolism, network pharmacology, detoxification

## Abstract

Licorice is well known for its ability to reduce the toxicity of the whole prescription in traditional Chinese medicine theory. However, honey-fired licorice (ZGC for short), which is made of licorice after being stir-fried with honey water, is more commonly used for clinical practice. The metabolism *in vivo* and detoxification-related compounds of ZGC have not been fully elucidated. In this work, the chemical constituents in ZGC and its metabolic profile in rats were both identified by high ultra-performance liquid chromatography quadrupole time-of-flight mass spectrometry (UPLC-Q-TOF-MS). The network pharmacology was applied to predict the potential detoxifying ingredients of ZGC. As a result, a total of 115 chemical compounds were identified or tentatively characterized in ZGC aqueous extract, and 232 xenobiotics (70 prototypes and 162 metabolites) were identified in serum, heart, liver, kidneys, feces, and urine. Furthermore, 41 compounds absorbed in serum, heart, liver, and kidneys were employed for exploring the detoxification of ZGC by network pharmacology. Ultimately, 13 compounds (five prototypes including P5, P24, P30, P41 and P44, and 8 phase Ⅰ metabolites including M23, M47, M53, M93, M100, M106, M118, and M134) and nine targets were anticipated to be potential mediums regulating detoxification actions. The network pharmacology analysis had shown that the ZGC could detoxify mainly through regulating the related targets of cytochrome P450 and glutathione. In summary, this study would help reveal potential active ingredients *in vivo* for detoxification of ZGC and provided practical evidence for explaining the theory of traditional Chinese medicine with modern technology.

## 1 Introduction

*Glycyrrhizae Radix et Rhizoma*, also named Gancao in Chinese or licorice in English, is the dried root and rhizoma of *Glycyrrhiza uralensis Fisch. or G. glabra L. or G. inflata Bat.* from the family Leguminosae (Li et al., 2019). Licorice is a traditional herb medicine that is regarded as “the progenitor of herbs” (Maria Pia et al., 2018) and has a unique function of detoxification. After stir-fried licorice with honey water, honey-fired licorice (short for ZGC) is obtained. It was originally mentioned in a traditional Chinese medicine (TCM) classics named “*Treatise on Cold Damage*” written by Zhongjing Zhang in the 3rd century. The chemical composition of licorice changed after processing with honey. Honey used as an adjuvant can accelerate the mutual transformation of liquiritin and isoliquirtin in ZGC (Ota and Makino, 2022). In addition, the content of liquiritin, liquiritin apioside, and glycyrrhizin in ZGC has significantly reduced as well (Wang et al., 2012). Concurrently, ZGC is widely used for detoxification in clinic based on TCM theory.

In TCM theory, the detoxification of licorice could be interpreted as the synergistic effect of reducing the toxicity of toxic herbs and the increased tolerance of toxicity in prescription. Licorice has been widely observed in combined usage with other herbal medicines, including *Aconiti Lateralis Radix Praeparata* (Sun et al., 2016), *Aconiti Kusnezoffii Radix* (Yan et al., 2017), *Sophorae Flavescentis Radix* (Shi et al., 2015), *Strychni Semen* (Gu et al., 2014), and so on. Moreover, it may contribute to reducing oxidative stress and further alleviating toxicity through regulation of the expression cytochrome P450 (CYP450) (Liu et al., 2019).

The published study indicated that the flavonoids and pentacyclic triterpene saponin were the major constituents of the licorice combined with toxic substances for the detoxification of licorice (Liu et al., 2019). Liquiritigenin and isoliquiritigenin generally exhibited potent inhibitory bioactivities against the CYP1A2 and CYP2C9 (Li et al., 2019). Glycyrrhetic acid-related metabolites could be induced by CYP3A4 (Hou et al., 2012). In addition, glycyrrhizin and glycyrrhetinic acid have a powerful antioxidant effect which could scavenge free radicals and further reduce the cardiac-toxic lipid peroxidation reaction (Chan et al., 2021). Nevertheless, the detoxification ingredients of ZGC have not been fully elucidated.

The research on the metabolic profile of drugs is valuable for a better understanding of the process which contains absorption, distribution, metabolism, and excretion after administration. The published studies about the metabolism of licorice have almost focused on serum, urine, and feces (Huang et al., 2018). Since heart, liver, and kidneys are vital organs for acting detoxification. Thus, it is of crucial interest to establish a sensitive and effective analytical method for a systematic study on the metabolic profile of ZGC in rat serum, heart, liver, kidneys, urine, and feces.

**FIGURE 1:**
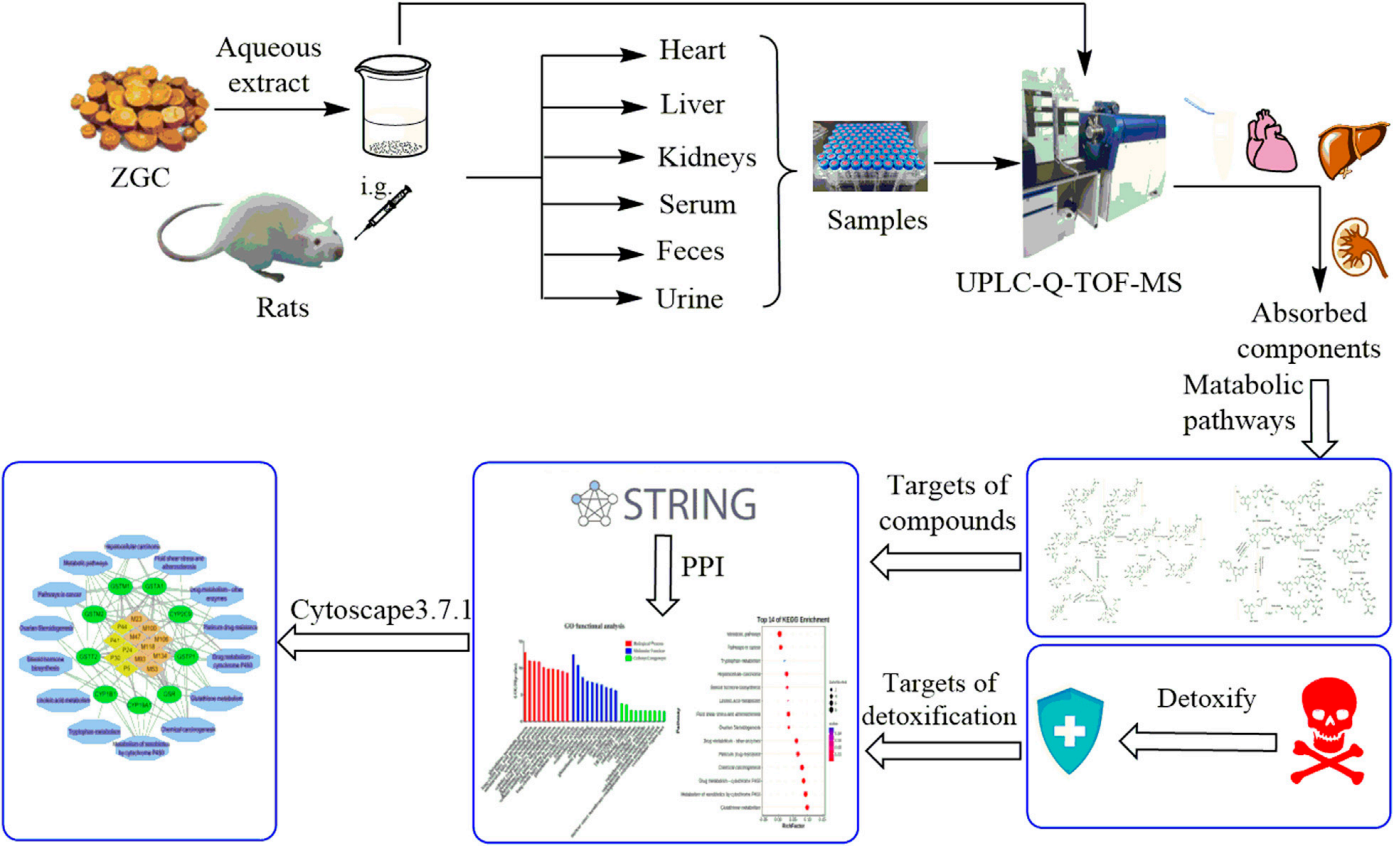
Workflow for metabolic profile and potential ingredients detoxification of ZGC.

In the present study, a fast and selective ultra-performance liquid chromatography quadrupole time-of-flight mass spectrometry (UPLC-Q-TOF-MS) was applied to characterize the chemical compounds of ZGC aqueous extract and metabolic profile *in vivo* (serum, heart, liver, kidneys, feces, and urine). Next, the prototypes and phase Ⅰ metabolites identified in serum, heart, liver and kidneys were screened for network pharmacology to explore the potential ingredients of detoxification in ZGC. The workflow was shown in Figure 1.

## 2 Materials and Methods

### 2.1 Materials and Reagents

ZGC (batch number: 181201191) was obtained from Kangmei Pharmaceutical Limited Corporation Co., Ltd (Nei Mongo, China). All ZGC samples were authenticated by associated professor Haibo Huang (Guangzhou University of Chinese Medicine, Guangzhou, China).

The reference standards for liquiritin (batch number: RP200207), liquiritin apioside (batch number: RP200107), ononin (batch number: RP200209), isoliquiritin (batch number: RP190308), liquiritigenin (batch number: RP200702), isoliquiritigenin (batch number: RP210115), glycyrrhizic acid (batch number: RP190517), glycyrrhizic acid ammonium salt (batch number: RP201104), and glycyrrhetinic acid (batch number: RP190512) were obtained from Chengdu Madsen Technology Co., Ltd. Chromatographic grade methanol (batch number: I1077207008), chmatographic acetonitrile (lot number: JB079830) and chromatformic acid (batch number: H158A) were purchased from Honeywell Trading Co., Ltd (Shanghai, China). Purified water was supplied by Watsons (Hong Kong, China). Oasis HLB solid-phase extraction (SPE) cartridge (6 cc/200 mg, 30 μm) was brought from Waters Technologies Co., Ltd. (Milford, America).

### 2.2 Standard Solutions

All the individual reference standard stock solutions in methanol were prepared with appropriate concentration, Then, the stock standard solutions were further diluted appropriately by methanol to prepare the mixed standard solution. The concentrations of liquiritin, liquiritin apioside, ononin, isoliquiritin, liquiritigenin, isoliquiritigenin, glycyrrhizic acid, glycyrrhizic acid ammonium salt, and glycyrrhetinic acid were 54.2, 99.8, 102.1, 87.3, 89.0, 56.3, 106.3, 79.3, and 102.3 μg/ml, respectively.

### 2.3 Preparation of ZGC Aqueous Extract

ZGC (30 g) was weighed and soaked in 300 ml water for 30 min at room temperature. After boiling for 2 h, the extract solution was filtered and concentrated to 50 ml. Finally, the ZGC aqueous extract at a concentration of 0.6 g/ml was prepared.

### 2.4 Animals and Drug Administration

Twelve healthy male rats weighing 220–250 g were obtained from the Experimental Animal Center of Guangzhou University of Chinese Medicine (Guangzhou, China). This study was approved by the Animal Ethics Committee of Guangzhou University of Chinese Medicine (No. 20190926003). The animals were bred in the Class SPF Experimental Animal Room of Guangzhou University of Chinese Medicine for a week. All of the rats were kept in an environment at a temperature of 20 ± 2°C with the dark-light for 12 h. Food and water were provided spontaneously to rats for 2 weeks. Before the experiment, these animals were fasted with free access to water in cages separately overnight. Twelve rats were randomized into two groups: blank and ZGC-dosed group. ZGC aqueous extract was given to rats at a dosage of 7.2 g/kg twice a day (Tan et al., 2010; Xiang et al., 2011; Qiao et al., 2012) for three continuous days. At the same time, the blank group was given the same dosage of normal saline. Three rats in each group were used for sampling serum, heart, liver, kidneys, and the other three rats were used for sampling urine and feces.

### 2.5 Biological Samples Collection and Pretreatment

#### 2.5.1 Serum Samples

The blood samples of rats (*n* = 3) (Lin et al., 2018; Hu et al., 2019) were collected from the orbital venous plexus into tubes at 0.25, 0.5, 1, 2, 4h after the last administration, then separated by centrifuging at 4,000 rpm for 10 min to attain serum. The pooled serum sample (2 ml) was processed 4-fold volume of methanol to precipitate the protein, followed by vortexing for 2 min and centrifuging at 14,000 rpm for 10 min to obtain the supernatant. The supernatant was dried through vacuum centrifugal concentrator on AL mode vent at 30°C, the supernatant was dried. The residues were reconstituted with 200 μl of 50% acetonitrile, vortexed for 1 min, and centrifuged at 14,000 rpm for 10 min. Finally, a 2 μl supernatant was injected into UPLC-Q-TOF-MS for analysis.

#### 2.5.2 Samples of Heart, Liver and Kidneys

Each group of rats (*n* = 3) was sacrificed at the last time point. The heart, liver, and kidneys were removed and washed with normal saline. The tissue samples (0.1 g) were homogenized in 1 ml normal saline under the condition of ice bath. The mixture was then centrifuged at 12,000 rpm for 10 min at 4°C to obtain the supernatant. Then, the 4-fold volume of methanol was added to the supernatant solution obtained to precipitate protein, followed by vortexing for 2 min and centrifuging at 12,000 rpm for 10 min. Through vacuum centrifugal concentrator on AL mode vent at 30°C, the supernatant was dried. The residues were reconstituted with 200 μl of 50% acetonitrile and vortexed for 1 min and centrifuged at 12,000 rpm for 10 min. Subsequently, 1 μl supernatant was injected into the UPLC-Q-TOF-MS system for analysis.

#### 2.5.3 Urine Samples

The urine samples of rats (*n* = 3) were collected from 0 to 12 h and 12–24 h after administration. The pooled urine sample (6 ml) was centrifuged at 4,000 rpm for 10 min. Then the 6 ml supernatant urine samples were added to pre-treated SPE columns and eluted with 0.1% formic acid and methanol, and washed with pure water once which would be discarded then. Then 0.1% formic acid and methanol were applied as the eluent for elution, with the further elution solution being collected. Through vacuum centrifugal concentrator on AL mode vent at 30°C, the supernatant was dried. The residues were reconstituted with 200 μl of 50% acetonitrile, vortexed for 1 min, and centrifuged at 14,000 rpm for 10 min. Finally, a 2 μl supernatant was injected into UPLC-Q-TOF-MS for analysis.

#### 2.5.4 Feces Samples

The feces samples of rats (*n* = 3) were collected from 0 to 12 h and 12–24 h after administration. All feces samples of the two periods were mixed and dried. The feces samples (1 g) were extracted with methanol, on which the ultrasonic was performed for 30 min under the condition of 500 W and 40 kHz, before being centrifuged at 14,000 rpm for 10 min. Through vacuum centrifugal concentrator on AL mode vent at 30°C, the supernatant was dried. The residues were reconstituted with 50% acetonitrile, vortexed for 1 min, and centrifuged at 14,000 rpm for 10 min. Finally, a 2 μl supernatant was injected into UPLC-Q-TOF-MS for analysis.

### 2.6 Instrument and Analysis Conditions

All samples were analyzed on an AB Sciex 5600 Triple-TOF™ mass spectrometer (AB Sciex, CA, United States) coupled with a Shimadzu UPLC LC-30AD system (Kyoto, Japan) and controlled with the Analyst TF 1.7 software (AB Sciex). Chromatographic separation was carried out at 30°C using a Hypersil GOLD aQ C18 column (Waters, United States; 2.1 mm × 100 mm, 1.9 μm). The mobile phase consisted of acetonitrile (A) and water (B) (both including 0.1% aqueous formic acid, v/v), and the flow rate was 0.4 ml/min. The gradient elution was performed as follows: 95% B at 0.0–1.0 min, 95–90% B at 1.0–2.0 min, 90–85% B at 2.0–4.0 min, 85–80% B at 4.0–6.0 min, 80–75% B at 6.0–8.0 min,75–65%B at 8.0–15.0 min, 65–55% B at 15.0–20.0 min, 55–5% B at 20.0–24.0 min, 5–0% B at 24.0–25.0 min, 0% B at 25.0–26.0 min. The mass spectrometer was coupled with an electrospray ionization (ESI) source, acquiring TOF-MS and TOF-MS-IDA-MS/MS spectra in the dynamic background subtraction by running in negative ion and high sensitivity mode. In addition, the automated calibration delivery system could acquire precise mass measurements. The operating parameters were as follows: Behind optimization, nitrogen was used as a source of gas, and the nebulizer gas, curtain gas, and heater gas were set at 55, 35 and 55 psi, respectively. The source temperature, ion spray voltage (ESI^+^/ESI^−^), and declustering potential were set at 550°C, 5500/−4500 V and 100/−100 V, respectively. For the IDA experiments, the collision energies were set at −45 and 45 eV, and the collision energy spread was set at 10/15 eV. TOF-MS spectra were obtained from 100 to 2,000 Da.

### 2.7 UPLC-Q-TOF-MS Data Processing

The self-building chemical library including compound name, molecular formula, structure and accurate molecular mass was established by literature, PubMed, Chemspider, CNKI, SciFinder, in which every single compound was numbered.

The characteristic chromatographic behaviors of available reference standards were obtained to identify the unknown compounds in ZGC aqueous extract. The molecular formula and order number of chemicals from the self-built library were imported into Peakview 1.5 to identify the chemical constituents of ZGC. The most rational molecular formula and the mass accuracy within a reasonable degree of measurement error (ppm <5) were gained. Meanwhile, compounds were identified by the fragmentation information obtained. Finally, LC-MS data file in positive and negative ion modes was applied to characterize ZGC-related xenobiotics (prototypes and metabolites) for biosamples from rats being administrated with ZGC. The representative prototypes were imported into MetabolitePilot 2.0.4 to search and identify the metabolites along with their metabolic pathways. The useful clogP values of compounds calculated by ChemDraw Ultra 19.0 was introduced to distinguish between structural isomers. In general, compounds had a longer retention time when the values of clogP were higher in reversed-phase liquid chromatography systems (Hallgas et al., 2004; Liang et al., 2013).

### 2.8 Network Pharmacology Study

#### 2.8.1 Prediction of Compounds and Targets of Detoxification in ZGC

The compounds (prototypes and phase Ⅰ metabolites) of ZGC identified from serum, heart, liver, and kidneys were imported into PharmMapper (Z′-score ≥ 1.5, http://www.lilab-ecust.cn/pharmmapper/) (Zhou et al., 2019) and Swiss Target Prediction (top 15, http://www.swisstargetprediction.ch/) (Daina et al., 2019) to predict the targets, respectively. Then, the corresponding target and uniport ID were obtained through Uniprot database (http://www.uniprot.org/). “Detoxify” were imported into GeneCards database (https://www.genecards.org/) to collect the detoxification-related targets.

The potential detoxification targets of ZGC were selected using Protein–Protein Interaction (PPI) with a high-combining score of more than 0.900 (Huang et al., 2019). The key compounds and targets were screened with the medians of Betweenness Centrality (BC), Closeness Centrality (CC), and Degree Centrality (DC) respectively by NetworkAnalyzer (a plug-in of Cytoscape).

#### 2.8.2 GO and KEGG Pathway Enrichment

Based on the mechanism of detoxification, the detoxification-related targets were further screened by existing literature. OmicShare (https://www.omicshare.com/) was used for Gene Ontology (GO) and Kyoto Encyclopedia of Genes and Genomes (KEGG) pathway enrichment analysis. The cut-off criterion of those GO and pathway terms was *p*-value < 0.05 (Qi et al., 2019), which was considered statistically significant.

## 3 Results

### 3.1 Determination of Ingredients in ZGC by HPLC

In this study, according to the method of the Chinese Pharmacopoeia 2020 edition, the ingredients in ZGC assayed by HPLC, the two content-determined compounds of ZGC, were liquiritin (6.11 mg/ml) and glycyrrhizic acid (33.88 mg/ml).

### 3.2 Identification and Characterization of Chemical Compounds

The exposed chemical components were identified by referring to relative retention time, accurate *m/z* values of quasimolecular ion, characteristic fragment ion, mass fragmentation rules from available databases and by comparing mass data with those from authentic standards and previous studies (Infante et al., 2012; He et al., 2014; Qiao et al., 2015). A total of six flavonoids and three pentacyclic triterpenoid saponins were exactly identified with reference standards, including liquiritin, liquiritin apioside, ononin, isoliquiritin, liquiritigenin, isoliquiritigenin, glycyrrhizic acid, glycyrrhizic acid ammonium salt, and glyrrhetinic acid. The chemical structures of nine reference standards of ZGC were shown in Supplementary Figure S1, and the detailed MS information of nine reference standards in ZGC was shown in Supplementary Table S1. The extracted ion chromatograms (EICs) of nine reference standards of ZGC were shown in Supplementary Figure S2. The fragment pathways of these chemical compounds have been well elucidated in published references (Xu et al., 2013; Zhou et al., 2013; Zhang et al., 2019). The spectrums and the proposed fragmentation pathways of these reference standards were shown in Supplementary Figures S3–S20. Meanwhile, a total of 115 chemical compounds were recognized and tentatively characterized by UPLC-Q-TOF-MS. Among them, these compounds included 57 flavonoids, 44 pentacyclic triterpenoids, 6 coumarins, and 8 other compounds. The base peak chromatograms (BPCs) of the chemical compounds of ZGC aqueous extract were depicted in Supplementary Figure S21, and the detailed MS information of these components were shown in Supplementary Table S2.

#### 3.2.1 Flavonoids

In the present study, a total of 57 flavonoids had been observed in ZGC aqueous extract. For aglycones, the characteristic fragment pathways were mainly Retro-Diels-Alder reaction (RDA), and the loss of radicals (CO, CO_2_, H_2_O, etc) and neutral small molecules. Compound 4 (Rt = 4.70 min, C_27_H_32_O_14_) was identified as liquiritigenin-in-7,4′-diglucoside, which exhibited characteristic ions at *m/z* 625.1783 [M + COOH]^-^, 417.1193 [M + COOH-HCOOH-C_6_H_10_O_5_]^-^, 255.0660 [M + COOH-HCOOH-2C_6_H_10_O_5_]^-^, with the further RDA fragmentation creating fragment pathway involved RDA with fragment ions at *m/z* 135.0088 [M + COOH-HCOOH-2C_6_H_10_O_5_-C_8_H_8_O]^-^. Compound 11 (Rt = 6.90 min, C_15_H_12_O_4_) showed [M + H]^+^ ion at *m/z* 257.0803 and obvious fragment ion at *m/z* 137.0235, which was due to RDA cleavage. The fragment ions at *m/z* 119.0498 and 109.0293 by the elimination of a H_2_O moiety or CO moiety of *m/z* 137.0235, suggested that it was tentatively characterized as pinocembrin. Compound 10 (Rt = 6.69 min, C_26_H_30_O_13_) gave [M-H]^-^ ion at *m/z* 549.1626, which was 162.0528 Da (glu) and 132.0423 Da (apiose) more than the fragment ion (*m/z* 255.0654) of aglycone. Compound 13 (Rt = 6.95 min, C_26_H_30_O_13_) gave the mother ion at *m/z* 549.1654, which was 132.0420 Da more than the fragment ion (*m/z* 417.1215) of aglycone. Compound 13 was characterized as an isomer of compound 10 because of their identical structural formulas. Moreover, the clogP values of compounds 10 and 13 were −0.52 and −0.27, respectively. And compounds 10 and 13 were characterized as liguiritigenin-7-O-D-apiosyl-4′-O-D-glucoside (Xu et al., 2013) and liquiritin apioside, respectively. Compound 18 (Rt = 9.31 min, C_16_H_12_O_4_), giving [M-H]^-^ ion at *m/z* 267.0691 which could spill into *m/z* 252.0453 and 135.0103, while the fragment ion at *m/z* 104.0330 was produced by loss of C_9_H_7_O_3_. These evidences allowed the tentative characterization of compound 18 as formononetin (Xu et al., 2013). Compound 27 (Rt = 10.46 min, C_16_H_12_O_5_) had [M + H]^+^ ion at *m/z* 285.0748, It produced *m/z* 270.0523 and 253.0483 fragments due to the elimination of the CH_3_ and CH_4_O, and the *m/z* 225.0538 was produced by the loss of CO. Thus, compound 27 was characterized as prunetin.

#### 3.2.2 Pentacyclic Triterpenoid Saponins

In the present study, a total of 44 pentacyclic triterpenoids had been observed in ZGC aqueous extract. They performed similar ion fragmentation; shared the same neutral loss of glucuronide (176 Da, gluA), the remaining aglycones fundamentally had some neutral small molecule loss such as -CH_3_, -CO, -H_2_O, etc.

Compound 30 (Rt = 11.42 min, C_42_H_62_O_17_) displayed [M-H]^-^ ion at *m/z* 837.3988 which cracked into *m/z* 661.3639 and 485.3227. Compound 58 (Rt = 15.89 min, C_42_H_62_O_17_) gave [M-H]^-^ ion at *m/z* 837.3989 which exhibited fragment ions at *m/z* 661.3646 [M-H-C_6_H_8_O_6_]^-^, 351.0577 [M-H-C_30_H_46_O_5_]^-^, 193.0356 [M-H-C_30_H_46_O_5_-C_6_H_8_O_5_]^-^ and 113.0253 [M-H-C_30_H_46_O_5_-C_6_H_8_O_5_-2H_2_O-CO_2_]^-^. Besides, they were defined as a pair of isomers sharing the same structural formula. Furthermore, Compounds 30 and 58 were assigned as macedenosin A and licoricesaponin G2 (Tao et al., 2013) based on clogP values of 1.65 and 1.74 by ChemDraw 14.0 software, respectively. Compound 38 (Rt = 12.96 min, C_48_H_74_O_19_) had [M+H]^+^ ion at *m/z* 955.4890. It produced *m/z* 779.4555 due to the elimination of C_6_H_8_O_6_. Then, the fragment could further eliminate C_6_H_8_O_6_ to produce *m/z* 603.4245, while the fragment ion at *m/z* 585.4128 was produced by the loss of H_2_O. Thus, compound 38 was characterized as uralsaponin T. Compound 43 (Rt = 14.17 min, C_42_H_60_O_17_) generated [M + H]^+^ ion at *m/z* 837.3823 and produced *m/z* 661.3575 fragment due to the cleavage of C_6_H_8_O_6_. While the fragment ions at *m/z* 643.3477 and 485.3245 by the elimination of H_2_O or C_6_H_8_O_6_, suggested that it was tentatively characterized as 3-O-[β-D-glu-curonopyranosyl-(1→2)-β-D-glucuronopyra-nosyl]-24-hydroxy-glabrolide. Compound 45 (Rt = 14.19 min, C_44_H_64_O_18_) had [M-H]^-^ ion at *m/z* 879.4096, which produced *m/z* 703.3753 by eliminating the moiety of C_6_H_8_O_6_. Then, the fragment could further wipe off C_2_H_4_O_2_ (60 Da) to yield *m/z* 643.3522, indicating the presence of an acetyl group. Thus, compound 45 was deduced to be 22β-acetoxyl-glycyrrhizin (Wang et al., 2015). Compound 69 (Rt = 17.77 min, C_41_H_62_O_14_) showed [M + H]^+^ at *m/z* 779.3824. It produced [M + H-C_11_H_16_O_10_]^+^ ion at *m/z* 471.3460 due to the elimination of gluA and arabinose. With fragment ions at *m/z* 471.3460 and 217.1573, which were consistent with those of glycyrrhetinic acid, compound 69 was thus inferred as araboglycyrrhizin.

#### 3.2.3 Coumarins

In this study, a total of 6 coumarins including compounds 1, 5, 48, 78, 98, and 106 were detected from ZGC aqueous extract.

Compound 78 (Rt = 19.56 min, C_21_H_20_O_6_) presented an [M-H]^-^ ion at *m/z* 367.1182. Its typical fragment ion at *m/z* 337.0745 was originated from the loss of CH_2_O, while the fragment ion at *m/z* 309.0405 was produced by the loss of C_3_H_5_ and OH. Hence, compound 78 was identified as glycycoumarin (Qiao et al., 2015). Compound 98 (Rt = 21.79 min, C_21_H_18_O_6_) exhibited the precursor ion [M-H]^-^ ion at *m/z* 365.1040. It had the characteristic dominant fragment ion at *m/z* 335.0556 and 307.0247, and was thus identified as glycyrol (Qiao et al., 2015).

#### 3.2.4 Other Compounds

The [M-H]^-^ ion at *m/z* 339.1261 of compound 91 (Rt = 21.09 min, C_20_H_20_O_5_) produced the fragments ions of [M-H-CH_3_]^-^ and [M-H-2CH_3_]^-^ at *m/z* 324.1014 and 309.0763, respectively, which indicated continuous loss of two molecules of CH_3_. Then, the remaining residue lost two carbonic oxides. It was determined as glycybridin A.

### 3.3 Identification of Prototypes *In Vivo*

**FIGURE 2:**
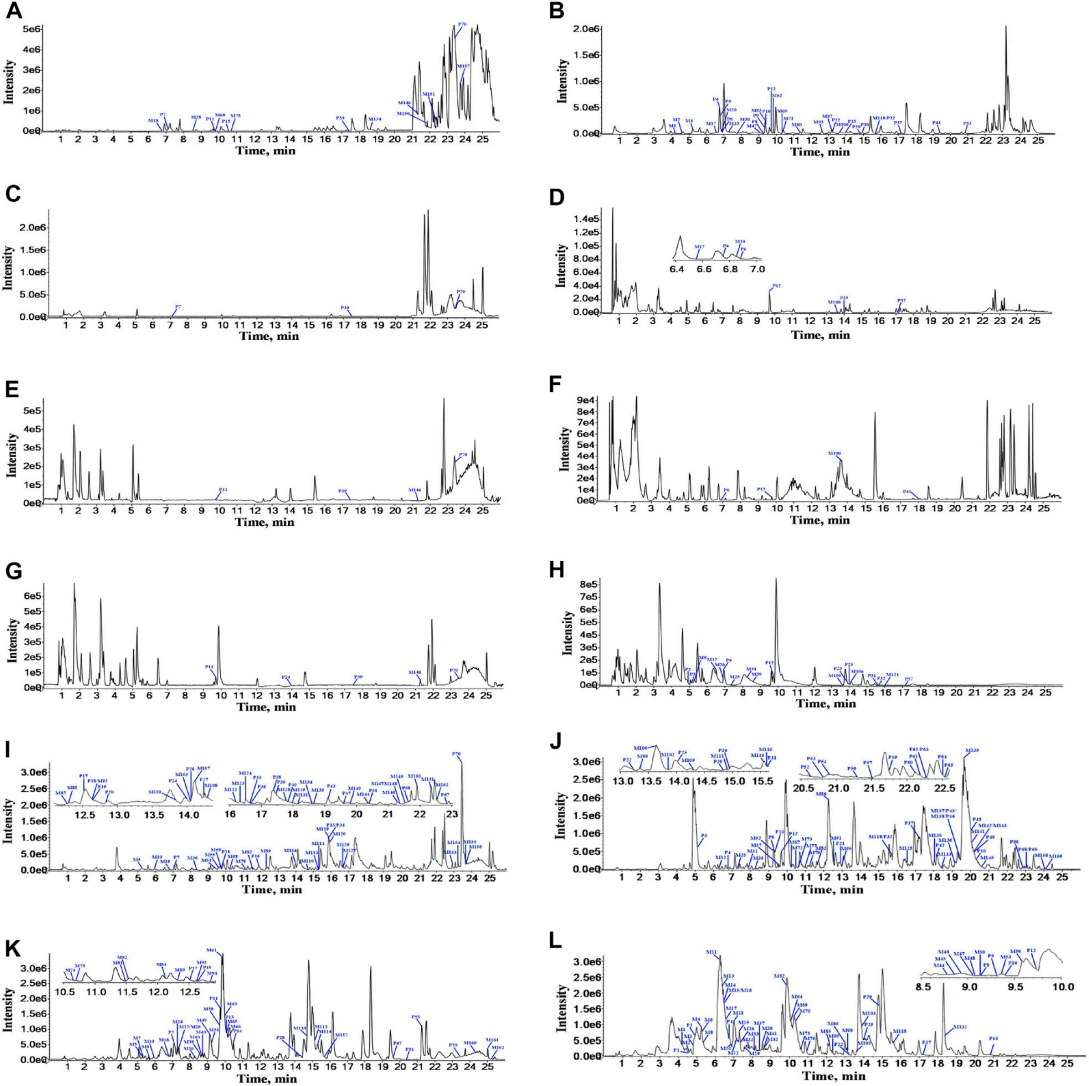
The BPCs of prototypes and metabolites after oral administration of ZGC in positive and negative ion modes. **(A)** serum in positive ion mode. **(B)** serum in negative ion mode. **(C)** heart in positive ion mode. **(D)** heart in negative ion mode. **(E)** liver in positive ion mode. **(F)** liver in negative ion mode. **(G)** kidneys in positive ion mode. **(H)** kidneys in negative ion mode. **(Ⅰ)** feces in positive ion mode. **(J)** feces in negative ion mode. **(K)** urine in positive ion mode. **(L)** urine in negative ion mode.

According to the retention time and MS fragments of chemical compounds in ZGC aqueous extract, a total of 70 prototypes were identified or tentatively characterized after oral administration of ZGC aqueous extract. Of these, 20 prototypes were identified in serum, 8 in heart, 6 in liver, 13 in kidneys, 57 in feces, and 23 in urine. The structures of these prototypes were mainly flavonoids and pentacyclic triterpenoid saponins. The data information of prototypes in rat biosamples after oral administration of ZGC by UPLC-Q-TOF-MS was listed in Supplementary Table S3. The BPCs of prototypes and metabolites after oral administration of ZGC in positive (A, C, E, G, I, K) and negative (B, D, F, H, J, L) ion mode were depicted in Figure 2.

### 3.4 Identification of ZGC-related Metabolites *In Vivo*

**FIGURE 3:**
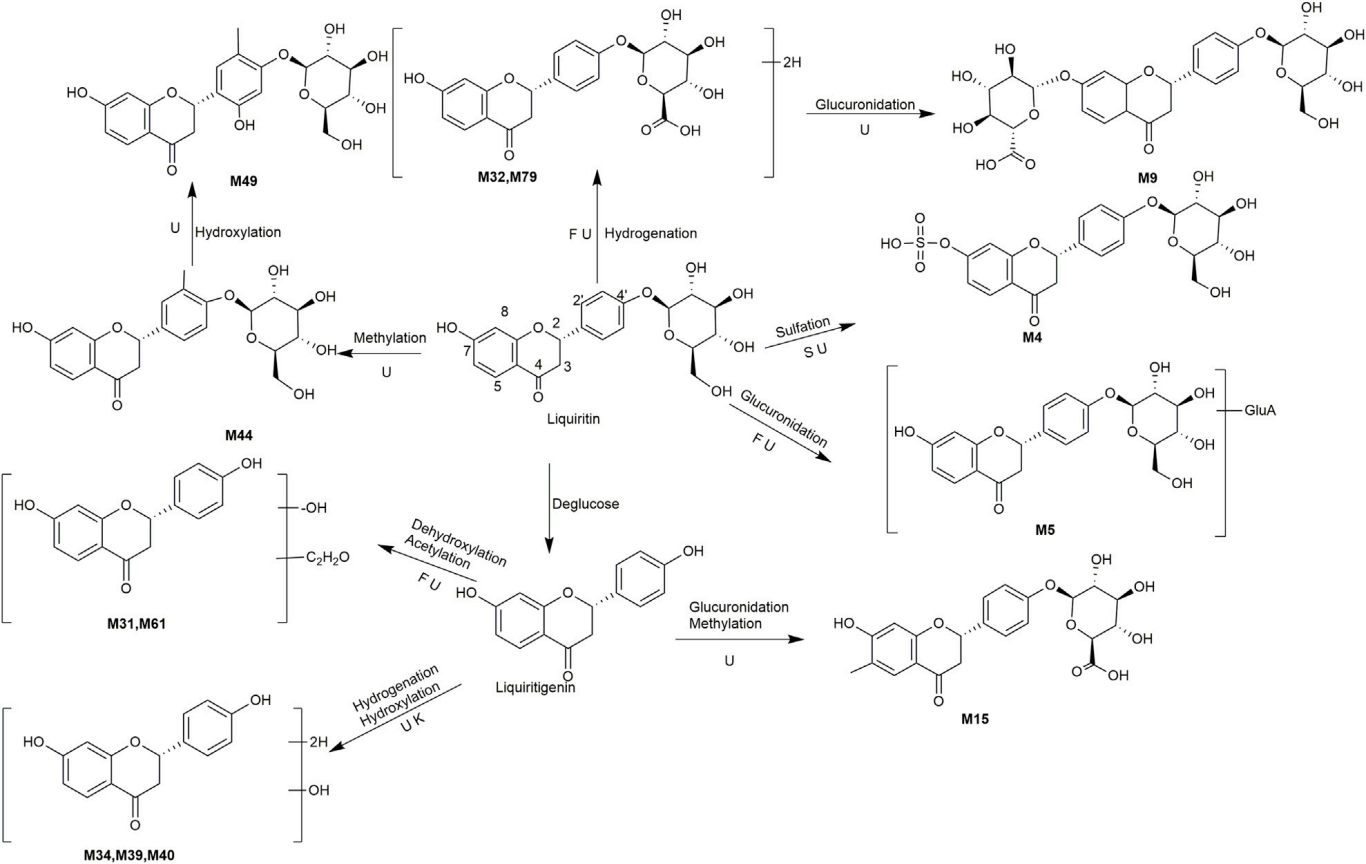
The proposed metabolic pathway of liquiritin-related metabolites in rats biosamples. (S, H, F and U represented serum, heart, feces and urine samples, respectively).

**FIGURE 4:**
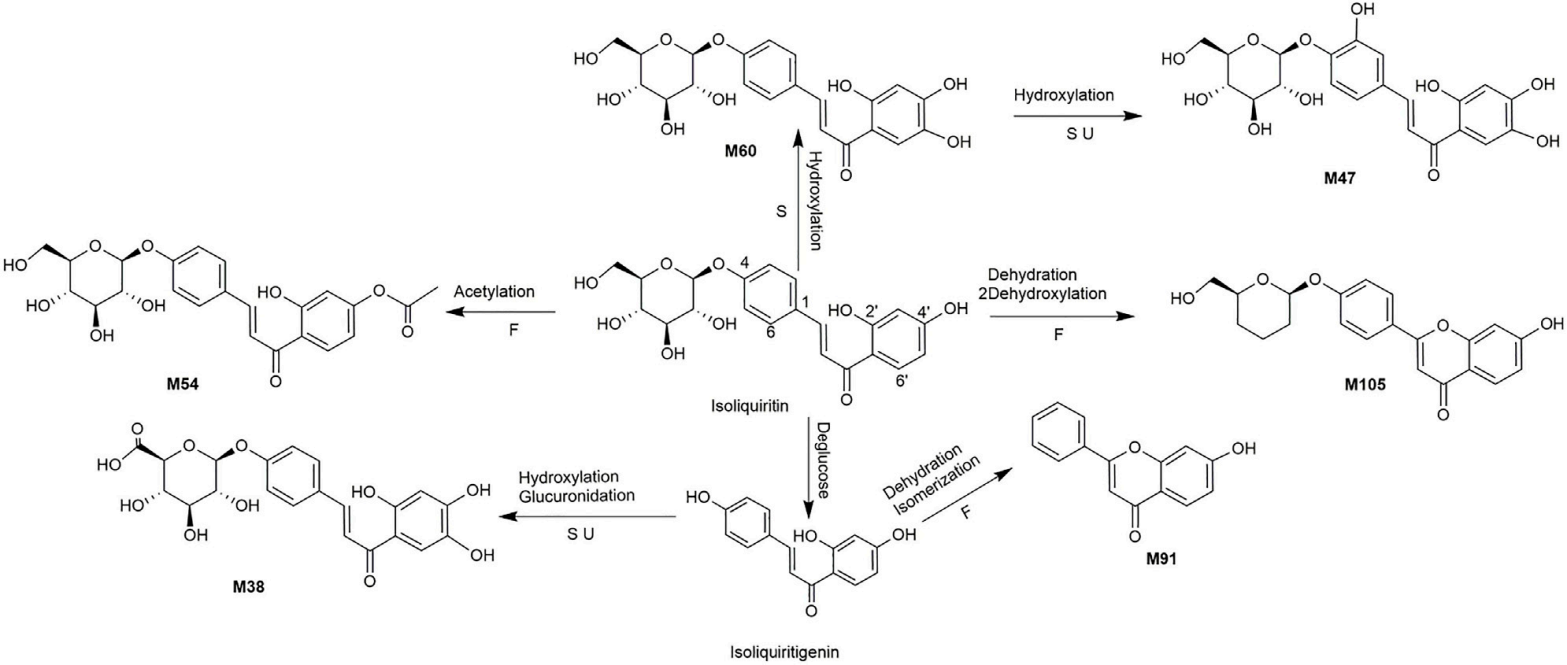
The proposed metabolic pathway of isoliquiritin-related metabolites in rats biosamples. (S, H, F and U represented serum, heart, feces and urine samples, respectively).

**FIGURE 5:**
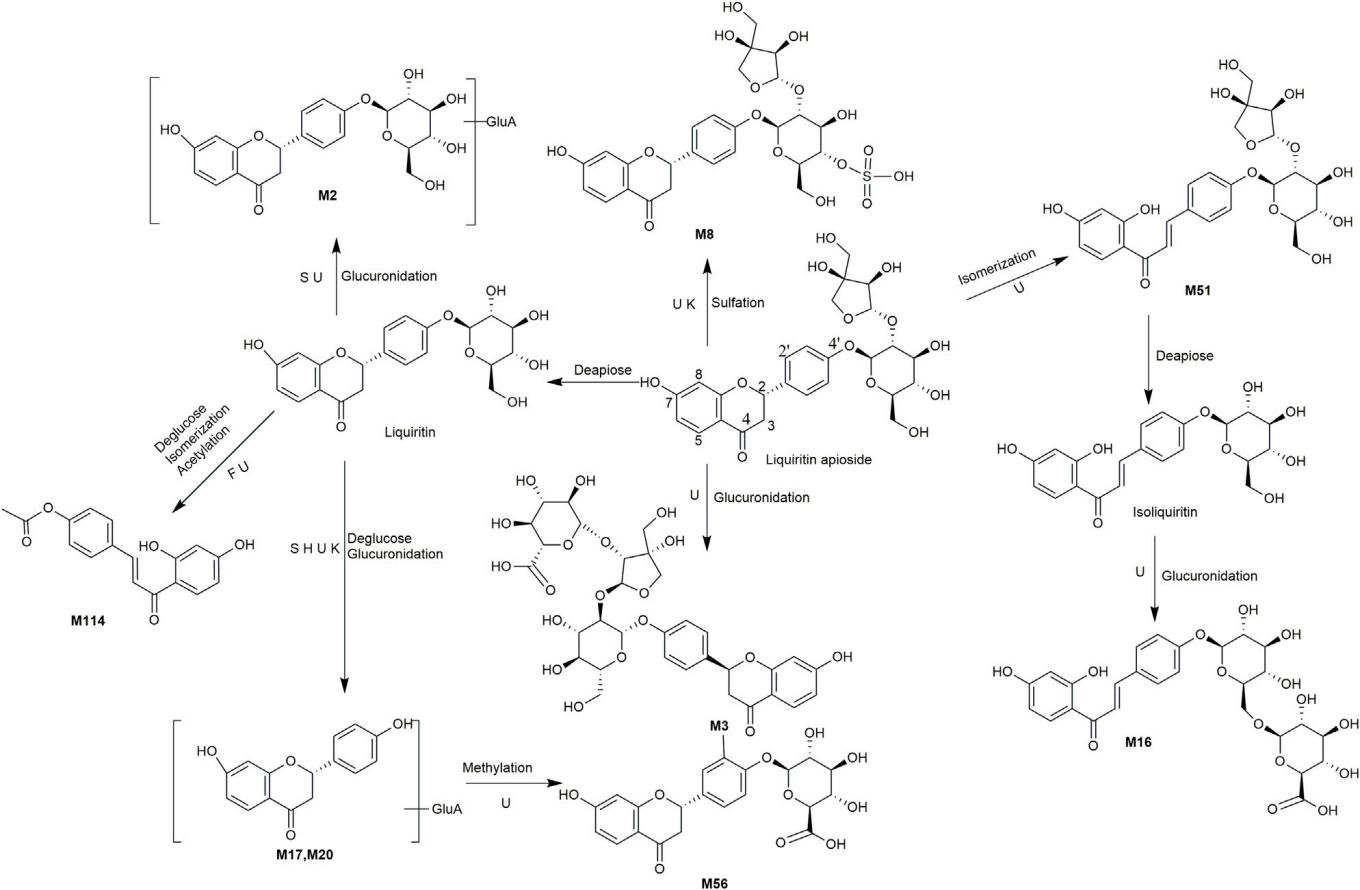
The proposed metabolic pathways of liquiritin apioside-related metabolites in rats biosamples. (S, H, F and U represented serum, heart, feces and urine samples, respectively).

**FIGURE 6:**
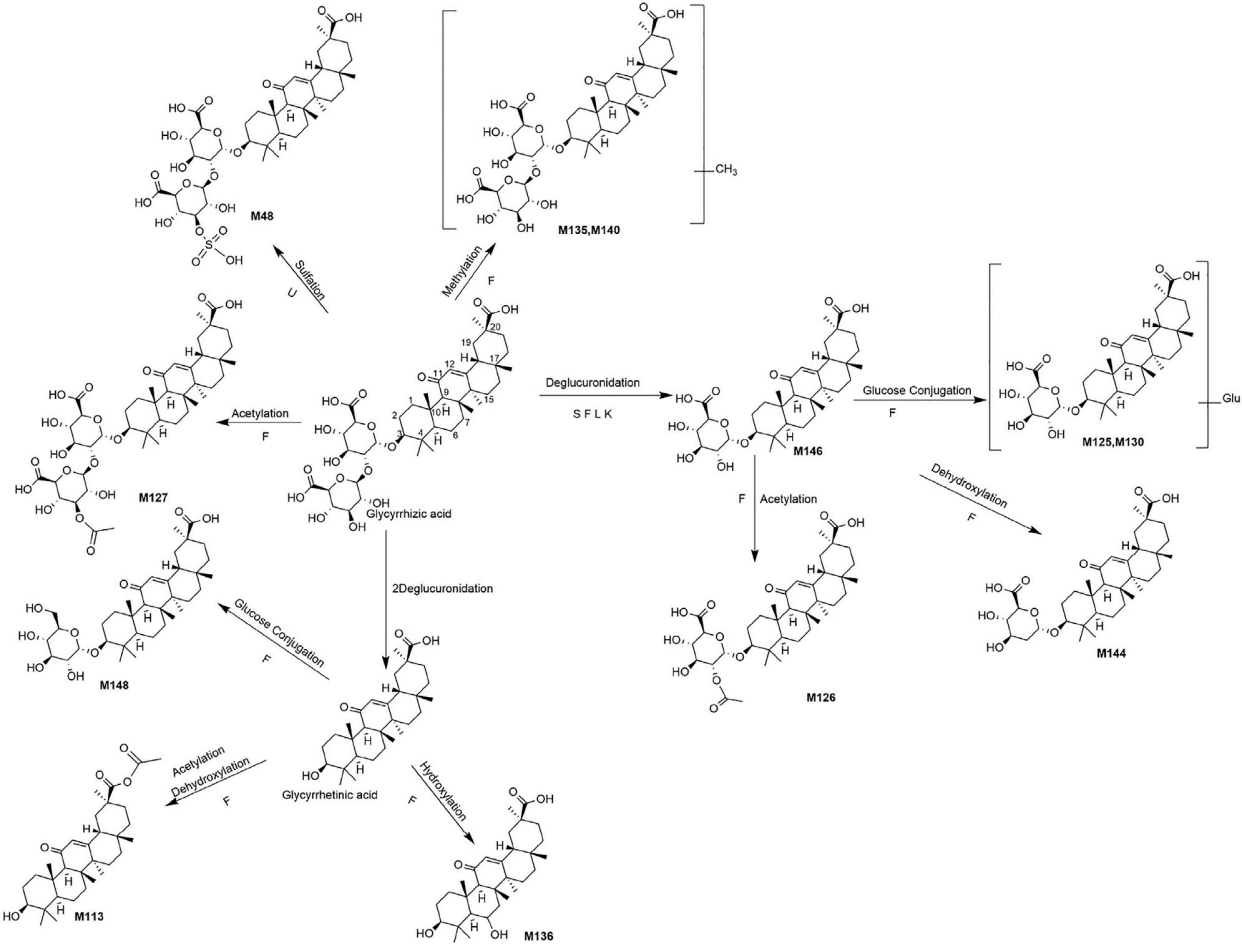
The proposed metabolic pathways of glycyrrihizic acid-related metabolites in rats biosamples. (S, H, F and U represented serum, heart, feces and urine samples, respectively).

A total of 162 metabolites (Supplementary Table S4) were identified or tentatively characterized after oral administration of ZGC aqueous extract. Most of them were flavonoid-related and saponin-related constituents, with 26 in serum, 3 in heart, 2 in liver, 10 in kidneys, 83 in feces and 80 in urine. These metabolites included the products of phase Ⅰ reduction and oxidation, and phase Ⅱ glycosylation, methylation, acetylation, glucuronidation, sulfation, hydrolysis, isomerization, and hydrogenation. The BPCs were depicted in Figure 2. The proposed metabolic pathways of ZGC in rats were shown in Figures 3–6. The typical metabolic reactions and the corresponding offsets of formula and mass data were proposed in Supplementary Table S5.

### 3.5 Identification of Flavonoid-Related Metabolites

M4 (Rt = 5.22 min, C_21_H_22_O_12_S) and M5 (Rt = 5.24 min, C_27_H_30_O_15_) were identified as the sulfated product and glucuronidated product of liquirtin, with the higher weight 80 Da (SO_3_) and 176 Da (gluA) than liquiritin (Qiao et al., 2012), correspondingly. M44 (Rt = 8.77 min, C_22_H_24_O_9_) showed an adduct [M + COOH]^-^ ion at *m/z* 477.1401, which was 14 Da (CH_2_) higher than that of liquiritin, giving rise to product ions at *m/z* 253, 135 and 117. It was supposed as the methylated product. Likewise, M49 (Rt = 9.14 min, C_22_H_24_O_10_) was defined as the methylated product of hydroxylated liquiritin. M32 (Rt = 7.85 min, C_21_H_24_O_9_) was supposed as the hydrogenated product of liquiritin because the molecular weight was 2 Da (2H) higher than that of liquiritin. Similarly, M79 (Rt = 11.24 min, C_21_H_24_O_9_) presented the same molecular formula as M32 and was thus assumed to be isomer. M9 (Rt = 5.69 min, C_27_H_32_O_15_) showed the [M + H]^+^ ions at *m/z* 597.2146, which was 176 Da (gluA) higher than that of M32 and M79. It is suggested as a hydrogenated product of glucuronidated liquiritin.

M31 (Rt = 7.83 min) and M61 (Rt = 9.79 min) were the acetylated and dehydroxylated metabolites of liquiritigenin, whose molecular formula was C_17_H_14_O_4_, with successive loss of O (16 Da) and conjugation of C_2_H_2_O (42 Da), and coinciding with a mass weight of 26 Da more than that of liquiritigenin. The clogP values of M31 and M61 were 2.82 and 2.93, relatively. Therefore, M31 and M61 were conjecturally characterized as liquiritin-Glu + C_2_H_2_O-O and its isomer, respectively. M34 (Rt = 8.25 min, C_15_H_14_O_5_), M39 (Rt = 8.56 min, C_15_H_14_O_5_) and M40 (Rt = 8.66 min, C_15_H_14_O_5_) were all identified as hydroxylation and hydrogenated product of liquiritigenin, respectively, because they were one more O (16 Da) and 2H (2 Da) than liquiritigenin. M15 (Rt = 6.49 min, C_22_H_22_O_10_) was speculated to be methylated product of glucuronidated liquiritigenin based on the characteristic fragment ion of [M + COOH-GluA-CO-CH_2_]^-^ at *m/z* 273.0757.

Our study demonstrated that liquiritin and liquiritigenin mainly experienced glucuronidation, sulfation, hydroxylation, hydrogenation, and methylation *in vivo*.

M54 (Rt = 9.46 min, C_23_H_24_O_10_) was generated by acetylation of isoliquiritin because the metabolite had an additional acetyl group of isoliquiritin. M105 (Rt = 14.07 min, C_21_H_20_O_6_) displayed [M + H]^+^ ion at *m/z* 369.1317 due to the loss of H_2_O and 2O from isoliquiritin, Thus, M105 was supposed as dehydroxylated product of dehydrated isoliquiritin. M60 (Rt = 9.76 min, C_21_H_22_O_10_) showed [M + H]^+^ at *m/z* 435.1266, which was 16 Da higher than isoliquiritin. Thus, M60 was characterized as the hydroxylated product of isoliquiritin. M47 (Rt = 9.05 min, C_21_H_22_O_11_) was characterized as the hydroxylated product of M60. M38 (Rt = 8.51 min, C_21_H_20_O_11_) provided [M + H]^+^ ion at *m/z* 449.2785, which was 192 Da higher than isoliquiritigenin, and was supposed as glucuronidated product of hydroxylated isoliquiritigenin (Huang et al., 2018). While ion at m/z 273 was originated from fragment ion at *m/z* 449 after the loss of gluA. M91 (Rt = 12.51 min, C_15_H_10_O_3_) gave an adduct [M + COOH]^-^ ion at *m/z* 283.0635, which was 18 Da (H_2_O) lower than isoliquiritigenin. M91 was identified as dehydrated product of isomerized isoliquiritigenin.

M3 (Rt = 4.80 min, C_32_H_38_O_19_) and M8 (Rt = 5.33 min, C_26_H_30_O_16_S) were identified as the glucuronidated and sulfated product of liquiritin apioside (Huang et al., 2018), respectively, because their molecular weights were 176 Da (gluA) and 80 Da (SO_3_) higher than liquiritin apioside. M51 (Rt = 9.23 min, C_26_H_30_O_13_) presented the same molecular formular as liquiritin apioside, and thus was characterized as its isomer. M16 (Rt = 6.49 min, C_27_H_30_O_15_) produced a deprotonated molecular [M-H]^-^ at *m/z* 593.1522, which was 176 Da (gluA) higher than liquiritin. The product ion at *m/z* 255.0665 [M-H-gluA-C_6_H_11_O_5_]^-^ suggested that it might be the glucuronidated product of liquiritin and further underwent isomerization. M2 (Rt = 4.56 min, C_27_H_30_O_15_) showed a deprotonated molecular [M-H]^-^ ion at *m/z* 593.1515, which was 176 Da (gluA) higher than liquiritin suggesting it as a glucuronidated product of liquiritin. M114 (Rt = 15.30 min, C_17_H_14_O_5_) provided a protonated molecular [M + H]^+^ ion at *m/z* 299.0911, with successive loss of glu (162 Da) and conjugation of C_2_H_2_O (42 Da), coinciding with a mass weight of 120 Da less than liquiritin. It was thus supposed as acetylated product of deglucosed liquiritin with further undergoing isomerization. M17 (Rt = 6.58 min, C_21_H_20_O_10_) and M20 (Rt = 6.85 min, C_21_H_20_O_10_) gave a deprotonated molecular [M-H]^-^ at *m/z* 431.0994 and 431.0993, which were produced from liquiritin by deglycosylation and glucuronidation, and this coincided with a mass weight of 14 Da lower than liquiritin. The characteristic fragment ions at *m/z* 255, 135, and 119 hinted that they might be glucuronidated product of deglucosed liquiritin. In addition, the clogP values of M17 and M20 were 0.27 and 0.56, respectively. Therefore, M17 was characterized as liquiritin apioside-apiose-Glu + gluA, and M20 as its isomer.

### 3.6 Identification of Saponin-Related Metabolites

M48 (Rt = 9.07 min, C_42_H_62_O_19_S) and M127 (Rt = 16.64 min, C_44_H_64_O_17_) were identified as the sulfation product and acetylation product of glycyrrhizic acid, separately, because the molecular weights were 80 Da (SO_3_) and 42 Da (C_2_H_2_O) higher than glycyrrhizic acid. Besides, M135 (Rt = 18.62 min, C_43_H_64_O_16_) and M140 (Rt = 19.78 min, C_43_H_64_O_16_) gave the [M + H]^+^ at *m/z* 837.3865 and 837.4226, and the molecular weight was 14 Da (CH_2_) higher than glycyrrhizic acid. Thus, they might be the methylated metabolites of glycyrrhizic acid. The glycyrrhizic acid could lose one or two molecules of glucuronic acid to produce glycyrrhizic acid-GluA (M146, Rt = 21.29 min, C_36_H_54_O_10_) (Huang et al., 2018) and glycyrrhetinic acid.

The MS/MS spectra of M125 (Rt = 16.51 min, C_42_H_64_O_15_) and M130 (Rt = 18.03 min, C_42_H_64_O_15_) exhibited similar fragment ions. In addition, M125 and M130 had the deprotonated molecule ions at *m/z* 807.4224 and 807.4217, respectively, which both weighed 162 Da (C_6_H_10_O_5_) higher than M146. So, they were considered to be the glucose conjugates of M146. The clogP values of M125 and M130 were 3.51 and 3.67, respectively. Therefore, M125 and M130 were tentatively characterized as glycyrrhizic acid-GluA + Glu and its isomer, respectively. Likewise, M148 (Rt = 21.35 min, C_36_H_56_O_9_) was characterized as glucose conjugates of glycyrrhetinic acid. M126 (Rt = 16.63 min) showed the [M-H]^-^ at *m/z* 689.3887, equalling C_38_H_56_O_11_ with a weight of 42 Da (C_2_H_2_O) greater than M146. Thus, M126 was characterized as the acetylated product of M146. Besides, M144 (Rt = 20.31 min, C_36_H_54_O_9_) was defined as dehydroxylated product of M146 due to a weight of 16 Da (O) less than M146. Similarly, M136 (Rt = 19.15 min, C_30_H_46_O_5_) weighing 16 Da (O) more than glycyrrhetinic acid, was defined as hydroxylated product of glycyrrhetinic (Lin et al., 2018), M113 (Rt = 15.29 min, C_32_H_48_O_4_) was deemed to be acetylated product of dehydroxylated glycyrrhetinic acid.

## 4 Network Pharmacology Study

### 4.1 Prediction of Compounds and Targets of Detoxification in ZGC

The network pharmacology had been used to construct the compound-target-disease network for exploring potential bioactive components. The active ingredients in TCM, including the prototypes and their metabolites, can be detected *in vivo.* A total of 26 prototypes and 15 phase Ⅰ metabolites identified in serum, heart, liver and kidneys after oral administration of ZGC (Supplementary Excel Sheet S1) were applied to the network pharmacology analysis.

As a result, 421 and 338 overlapping targets related to the 26 prototypes and 15 phase Ⅰ metabolites were obtained respectively (Supplementary Excel Sheets S2,S3), while 1345 detoxification-associated targets were obtained from GeneCards database (Supplementary Excel Sheet S4). In the topological analysis of PPI network, 388 and 377 hub nodes were selected (Supplementary Excel Sheet S5) by NetworkAnalyzer. Eventually, 13 active compounds, including 5 prototypes (compounds P5, P24, P30, P41 and P44) and 8 phase Ⅰ metabolites (M23, M47, M53, M93, M100, M106, M118, and M134) were speculated to be concerned with 111 (98 and 74 targets of prototypes and phase Ⅰ metabolites, respectively) targets that played roles in detoxification (Supplementary Excel Sheets S6–S8).

### 4.2 The Analysis Results of GO and KEGG

**FIGURE 7:**
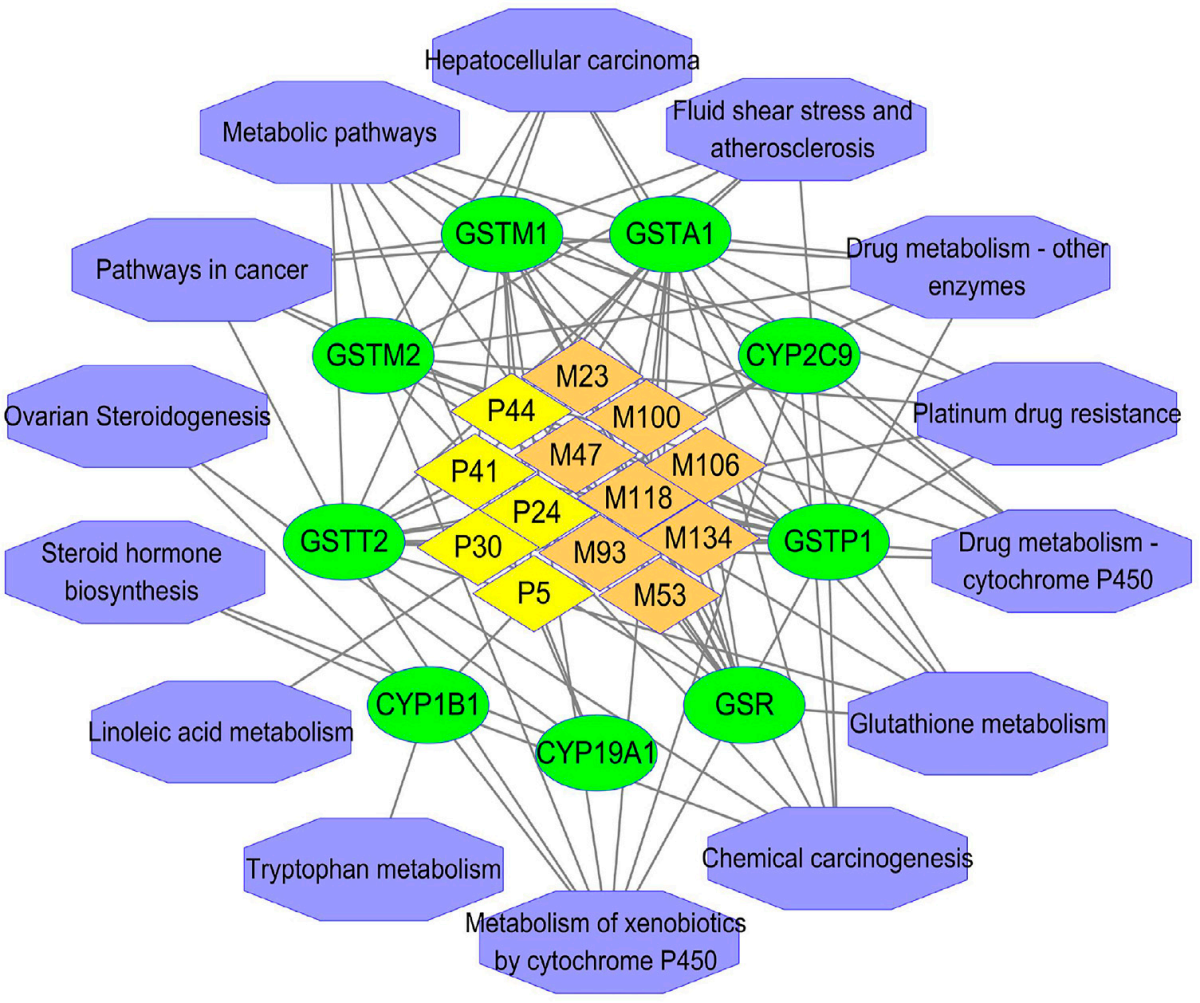
The “compound-target-signaling pathway” network. The yellow, orange, green and purple represented prototypes, phase Ⅰ metabolites, the corresponding key targets and signaling pathways, respectively.

To further clarify the mechanism of detoxification of ZGC, targets were utilized for GO and KEGG enrichment analysis. Based on existing literature, nine putative detoxification targets were screened out of 111 targets, which were also intersected with 13 active compounds (Supplementary Excel Sheet S9). These nine targets were mainly CYP450-related ones including CYP19A1, CYP1B1, CYP2C9, and GSH-related ones including Glutathione reductase, mitochondrial (GSR), Glutathione S-transferase A1 (GSTA1), Glutathione S-transferase Mu 1 (GSTM1), Glutathione S-transferase Mu 2 (GSTM2), Glutathione S-transferase P (GSTP1) and Glutathione S-transferase theta-2 (GSTT2). The GO analysis results indicated that nine putative detoxification targets were engaged in 272 BP, 65 MF, and 19 CC (Supplementary Excel Sheet S10). As for the KEGG enrichment outcomes, it turned out that nine putative detoxification targets might contribute to 14 pathways (Supplementary Excel Sheets S12,S13). Among those pathways, metabolism of xenobiotics by cytochrome P450, glutathione metabolism, drug metabolism-cytochrome P450 were entwined with more targets and closely correlated with detoxification. As shown in Supplementary Figure S22, the top 10 enrichment terms in BP, MF and CC were drawn by GO secondary classification histogram based on GraphPad Prism 7. The pathways depicted by a bubble chart were listed in Supplementary Figure S23. In Figure 7, the entangled reactions among the 13 active compounds, nine putative detoxification targets, and 14 detoxification-related pathways were comprehensively illustrated by Cytoscape 3.7.1.

## 5 Discussion

In this study, UPLC-Q-TOF-MS was used to probe the chemical profile and metabolic profile *in vivo* of ZGC aqueous extract. Eventually, 115 chemical compounds were characterized. For flavonoid glycosides, it mainly underwent deglycosylation, yielding fragment ions of flavonoids with higher abundance (Xu et al., 2013). We also found that dihydroflavones and chalcones missed fragments (C_8_H_8_O and C_7_H_4_O_3_) by RDA. Furthermore, segments of CO and H_2_O were usually featured by a neutral loss of 28 and 18 Da, correspondingly. Similarly, pentacyclic triterpenoid saponins experienced deglycosylation easily. The cleavage of allyl (between C-7 and C-8) along with characteristic fragment ion at *m/z* 317 caused by McLafferty rearrangement were prone to occur under positive ion mode. Additionally, RDA was occurred to yield characteristic fragment ion at *m/z* 263 (Shi et al., 2012).

As for metabolites, we found that the products related with liquiritin, isoliquiritin, liquiritin apiose, and glycyrrhizic acid were the major metabolites of ZGC. Flavonoid was undoubtedly one of the most prevalent metabolites in rat biosamples (Xiang et al., 2011). A total of 144 flavonoids including 35 prototype compounds and 109 metabolites were identified in rats. Triterpene saponins were another major group of bioactive compounds, including 25 prototype compounds and 53 metabolites. Based on the identified metabolites, the possible major metabolic pathways in ZGC were proposed. The primary *in vivo* biotransformation of ZGC included phase Ⅰ reduction oxidation and phase Ⅱ methylation, acetylation, glucosylation, glucuronidation and sulfation. Thus, the metabolite whose molecular weight showed a difference value of 16, 14, 2, 42, 80, 162, or 176 Da with the parent compounds might usually be detected. The biosamples with the most amounts of prototypes and metabolites were urine and feces, which suggested that most of the compounds experienced the biotransformation of phase Ⅰ and phase Ⅱ and were excreted out of the body eventually. Among them, flavonoids exposed more frequently had more in urine due to the increased polarity after conjugation with glucuronidation, sulfate conjugation, et al, which were apt to be excreted. There was more triterpene in feces since it was probably excreted through intestinal metabolism (Zhao et al., 2012). Furthermore, 26 metabolites containing 20 flavonoids and 6 triterpenes were detected in the serum, rendering potential active compounds. All these identified metabolites in this work could be used to elucidate the material basis of ZGC.

To conduct further research, a network pharmacology method had been developed to reveal potential targets of the prototypes and phase Ⅰ metabolites separately absorbed in serum, heart, liver, and kidneys after oral administration of ZGC. That is due to the consideration of the strong detoxification of the liver and the excretion of the kidneys, as well as the metabolites of ZGC indicating that they could be absorbed into the circulation and might be responsible for detoxification accordingly. Through a series of screening, we discovered 13 potentially active compounds and nine corresponding targets that regulating 14 signaling pathways on detoxification of ZGC. Among them, 1 flavonoid and 4 triterpene saponins in the prototypes, 6 flavonoids and 2 triterpene saponins in the metabolites were involved in screening. Co-related targets of absorbed constituents were CYP450-related (CYP19A1, CYP1B1, and CYP2C9) and GSH-related (GSR, GSTA1, GSTM1, GSTM2, GSTP1, and GSTT2). CYP450s are important phase Ⅰ DMEs (drug-metabolizing enzymes) in detoxification and exist a large amount in the gastrointestinal tract, liver, and kidneys (Wu et al., 2018). CYP1, 2 and 3 families are considered responsible for about 80% metabolism of clinical drugs (Zhao et al., 2021). Furthermore, CYP2C9 had greater effectiveness than others when it came to detoxification (Schleiff et al., 2021). In humans and mammals, the conjugates of GSH and reactive compounds are considered one of the most important pathways of detoxification. Prior studies have reported (Kim and Lee, 2007) that GSTs were responsible for the cellular metabolism correlated with detoxification. In addition, GST and GR were involved in GSH metabolism and as part of detoxification mechanism.

This analytical strategy systematically investigated the potential compounds associated with detoxification based on metabolic analysis and network pharmacology analysis. However, it is a pity that the active compounds and targets predicted by network pharmacology have not been confirmed and validated by experiments *in vitro* and *in vivo*.

## 6 Conclusion

In this work, UPLC-Q-TOF-MS was used to analyze the chemical and metabolic profiles of ZGC. 115 chemical compounds of ZGC aqueous extract and 232 xenobiotics (70 prototypes and 162 metabolites) in rats were identified. Furthermore, 26 prototypes and 15 metabolites in serum, heart, liver, and kidneys were used to construct network pharmacology explaining the detoxification of ZGC. The results have shown that the effects of 13 potential active compounds (5 prototypes and 8 metabolites) on the nine potential targets (CYP19A1, CYP1B1, CYP2C9, GSR, GSTA1, GSTM1, GSTM2, GSTP1, and GSTT2) were associated with CYP450s and GSH for detoxification of ZGC. Based on it, the potential active compounds, 5 prototypes (P5, P24, P30, P41 and P44) and 8 phase Ⅰ metabolites (M23, M47, M53, M93, M100, M106, M118, and M134), could be detoxification essence of ZGC. Together, this study tried to clarify the potential active ingredients of detoxification and the possible mechanism of detoxification on ZGC.

## Data Availability

The original contributions presented in the study are included in the article/Supplementary Material, and further inquiries can be directed to the corresponding author.

## Abbreviations

ZGC, honey-fired licorice; UPLC-Q-TOF-MS, ultra-performance liquid chromatography quadrupole time-of-flight mass spectrometry; TCM, traditional Chinese medicine; GO, Gene Ontology; KEGG, Kyto Encyclopedia of Genes and Genomes; ESI, electrospray ionization; BC, betweenness centrality; CC, closeness centrality/cellular component; DC, degree centrality; BP, biological process; MF, molecular function; EICs, extracted ion chromatograms; BPCs, base peak chromatograms; RDA, Retro-Diels-Alder; CYP450, cytochrome P450; GSH, glutathione; GSR, glutathione reductase, mitochondrial; GSTA1, glutathione S-transferase A1; GSTM1, glutathione S-transferase Mu 1; GSTM2, glutathione S-transferase Mu 2; GSTP1, Glutathione S-transferase P; PPI, Protein-Protein Interaction; GSTT2, glutathione S-transferase theta-2.
