# Supplementary material for: Explore the Potential Ingredients for Detoxification of Honey-Fired Licorice (ZGC) Based on the Metabolic Profile by UPLC-Q-TOF-MS
Source: Front Chem. 2022 Jul 13;10:924685. doi: 10.3389/fchem.2022.924685 (PMC9335949; doi:10.3389/fchem.2022.924685)
Supplement: Supplementary file 1 [file DataSheet1.docx]

**Supplementary information**

**Explore the Potential Ingredients for Detoxification of Honey-Fired Licorice (ZGC) Based on the Metabolic Profile by UPLC-Q-TOF-MS**

**Yinjie Wang^1†^, Yu Ning^2†^, Ting He^3^, Yingtong Chen^1^, Wenhui Han^1^, Yinping Yang^1^, Cui-xian Zhang^1^***

^†^These authors share first authorship.

^1^School of Pharmaceutical sciences, Guangzhou University of Chinese Medicine, Guangzhou, China

^2^Ningxia Chinese Medicine Research Center, Yinchuan, China

^3^Ningxia Hui Medicine Research Institute, Yinchuan, China

*** Correspondence:**Cuixian Zhang
[zhangcuixian@gzucm.edu.cn](mailto:zhangcuixian@gzucm.edu.cn).

**Contents：**

**Table S1** Identification of 9 reference standards of ZGC by UPLC-Q-TOF-MS.

**Table S2** Identification of ZGC aqueous extract chemical compounds by UPLC-Q-TOF-MS.

**Table S3** Identification of prototypes in rat biosamples after oral administration of ZGC by UPLC-Q-TOF-MS.

**Table S4** Identification of metabolites in rat biosamples after oral administration of ZGC by UPLC-Q-TOF-MS.

**Table S5** Typical metabolic reaction and the corresponding offsets of formula and mass.

**Figure S1** The chemical structures of 9 reference standards of ZGC.

**Figure S2** The EICs of 9 reference standards of ZGC by UPLC-Q-TOF-MS in positive (A) and negative ion mode (B).

**Figure S3** The spectrum of liquiritin in negative ion mode.

**Figure S4** The proposed fragmentation pathway of liquiritin in negative ion mode.

**Figure S5** The spectrum of liquiritin apioside in negative ion mode.

**Figure S6** The proposed fragmentation pathway of liquiritin apioside in negative ion mode.

**Figure S7** The spectrum of ononin in negative ion mode.

**Figure S8** The proposed fragmentation pathway of ononin in negative ion mode.

**Figure S9** The spectrum of isoliquiritin in negative ion mode.

**Figure S10** The proposed fragmentation pathway of isoliquiritin in negative ion mode.

**Figure S11** The spectrum of liquiritigenin in negative ion mode.

**Figure S12** The proposed fragmentation pathway of liquiritigenin in negative ion mode.

**Figure S13** The spectrum of isoliquiritigenin in negative ion mode.

**Figure S14** The proposed fragmentation pathway of isoliquiritigenin in negative ion mode.

**Figure S15** The spectrum of glycyrrhizic acid in negative ion mode.

**Figure S16** The proposed fragmentation pathway of glycyrrhizic acid in negative ion mode.

**Figure S17** The spectrum of glycyrrhizic acid ammonium salt in positive ion mode.

**Figure S18** The proposed fragmentation pathway of glycyrrhizic acid ammonium salt in positive ion mode.

**Figure S19** The spectrum of glycyrrhetinic acid in positive ion mode.

**Figure S20** The proposed fragmentation pathway of glycyrrhetinic acid in positive ion mode.

**Figure S21** The base peak chromatograms (BPCs) of the chemical compounds of ZGC aqueous extract by UPLC-Q-TOF-MS in positive ion mode (A) and in negative ion mode (B).

**Figure S22** The GO secondary classification histogram of the top 10 of biological process, cellular component and molecular function terms.

**Figure S23** The bubble of top 14 pathways of key targets from prototypes and phase Ⅰ metabolites against detoxification.

**Table S1** Identification of 9 reference standards of ZGC by UPLC-Q-TOF-MS.

| **No.** | **RT**  (min) | **Selected ion** | **Formula** | **Measured**  **ion** | **Mass (Da)** | **Error**  **(ppm)** | **Identification** | **MS /MS fragmentation** |
| --- | --- | --- | --- | --- | --- | --- | --- | --- |
| 1 | 6.86 | [M-H]^-^ | C_21_H_22_O_9_ | 417.1189 | 418.1264 | -0.59 | Liquiritin | 417.1174[M-H]-;255.0640[M-H-C6H10O5]-;135.0076[M-H-C6H10O5-C8H8O]-;119.0503[M-H-C6H10O5-C8H8O-O]-;213.0577[M-H-C6H10O5-C2H2O]-;119.0503[M-H-C6H10O5-C7H4O3]- |
| 2 | 6.90 | [M-H]^-^ | C_26_H_30_O_13_ | 549.1609 | 550.1686 | -0.80 | Liquiritin apioside | 549.1610[M-H]-;417.1182[M-H-C5H8O4]-;255.0645[M-H-C5H8O4-C6H10O5]-;135.0076[M-H-C5H8O4-C6H10O5-C8H8O]-;119.0500[M-H-C5H8O4-C6H10O5-C8H8O-O]-;429.1182[M-H-C7H4O2]-;387.1068[M-H-C7H4O2-C2H2O]-;297.0757[M-H-C5H8O4-C7H4O2]-;119.0500[M-H-C5H8O4-C6H10O5-C7H4O3]- |
| 3 | 9.39 | [M+COOH]^-^ | C_22_H_22_O_9_ | 475.1235 | 430.1264 | -0.29 | Ononin | 475.1237[M+COOH]-;267.0637[M+COOH-HCOOH-C6H10O5]-;252.0407[M+COOH-HCOOH-C6H10O5-CH3]-;223.0389[M+COOH-HCOOH-C6H10O5-CO-O]- |
| 4 | 9.52 | [M-H]^-^ | C_21_H_22_O_9_ | 417.1186 | 418.1264 | -1.16 | Isoliquiritin | 417.1207[M-H]-;255.0654[M-H-C6H10O5]-;135.0086[M-H-C6H10O5-C8H8O]-;119.0505[M-H-C6H10O5-C7H4O3]-;213.0552[M-H-C6H10O5-C2H2O]-;148.0158[M-H-C6H10O5-C6H3O2]- |
| 5 | 9.89 | [M-H]^-^ | C_15_H_12_O_4_ | 255.0663 | 256.0736 | -0.10 | Liquiritigenin | 255.0642[M-H]-;213.0543[M-H-C2H2O]-;135.0075[M-H-C8H8O]-;119.0504[M-H-C8H8O-O]-;119.0504[M-H-C7H4O3]- |
| 6 | 15.04 | [M-H]^-^ | C_15_H_12_O_4_ | 255.0664 | 256.0736 | -0.32 | Isoliquiritigenin | 255.0643[M-H]-;213.0542[M-H-C2H2O]-;135.0085[M-H-C8H8O]-;119.0504[M-H-C8H8O-O]-;119.0504[M-H-C7H4O3]- |
| 7 | 17.42 | [M-H]^-^ | C_42_H_62_O_16_ | 821.3983 | 822.4038 | 2.23 | Glycyrrhizic acid | 821.4009[M-H]-;645.3682[M-H-C6H8O6]-;469.3349[M-H-2C6H8O6]-;351.0547[M-H-C30H46O4]-;193.0338[M-H-C30H46O4-C6H8O5]-;113.0248[M-H-C30H46O4-C6H8O5-2H2O-CO2]- |
| 8 | 17.62 | [M+H]^+^ | C_42_H_62_O_16_ | 840.4323 | 839.4303 | -6.37 | Glycyrrhizic acid ammonium salt | 840.4323[M+H]+;823.3969[M+H-NH3]+;647.3724[M+H-C6H8O6]+;471.3428[M+H-2C6H8O6]+;453.3311[M+H-2C6H8O6-H2O]+;217.1579[M+H-2C6H8O6-2H2O-CO-C14H22]+ |
| 9 | 23.50 | [M+H]^+^ | C_30_H_46_O_4_ | 471.3439 | 470.3396 | -6.28 | Glycyrrhetinic acid | 471.3399[M+H]+;453.3306[M+H-H2O]+;425.3356[M+H-H2O-CO]+;317.2070[M+H-C10H17O]+;217.1562[M+H-C14H24O-CO-H2O]+;189.1605[M+H-C16H23O3-H-H2O]+;175.1446[M+H-C16H23O3-H2O-CH3]+ |

**Table S2** Identification of ZGC aqueous extract chemical compounds by UPLC-Q-TOF-MS.

| **No.** | **RT**  **(min)** | **Selected ion** | **Formula** | **Measured**  **mass** | **Mass**  **(Da)** | **Error**  **(ppm)** | **Identification** | **MS /MS fragmentation** |
| --- | --- | --- | --- | --- | --- | --- | --- | --- |
| 1 | 3.25 | [M-H]- | C12H14O8 | 285.0618 | 286.0689 | 0.90 | Uralenneoside | 285.0582[M-H]^-^;203.8335[M-H-C_4_H_2_O_2_]^-；^153.0193[M-H-C_5_H_8_O_4_]^-^;135.9145[M-H-C_5_H_8_O_4_-H_2_O]^-^;108.0222[M-H-C_6_H_10_O_6_-CO]^-^ |
| 2 | 4.43 | [M-H]- | C28H32O16 | 623.1627 | 624.1690 | 1.57 | Isorhamnetin-3-O-neohespeidoside | 623.1627[M-H]^-^;577.1457[M-H-CH_2_O_2_]^-^；415.1022[M-H-CH_2_O_2_-C_6_H_10_O_5_]^-^；295.0635[M-H-C_7_H_12_O_6_-C_7_H_4_O_3_]^-^;267.0621[M-H-CH_2_O_2_-C_6_H_10_O_5_-C_6_H_12_O_4_]^-^ |
| 3 | 4.43 | [M+COOH]- | C27H30O14 | 623.1627 | 578.1636 | 3.33 | Isoviolanthin | 623.1627[M+COOH]^-^;415.1022[M+COOH-HCOOH-C_6_H_10_O_5_]^-^;295.0635[M+COOH-HCOOH-C_6_H_10_O_5_-C_8_H_8_O]^-^;267.0621[M+COOH-HCOOH-C_6_H_10_O_5_-C_8_H_8_O-CO]^-^;253.0489[M+COOH-HCOOH-C_6_H_10_O_5_-C_9_H_7_O_3_]^-^ |
| 4 | 4.70 | [M+COOH]- | C27H32O14 | 625.1783 | 580.1792 | 3.12 | Liquiritigenin-7,4′-diglucoside | 625.1783[M+COOH]^-^;417.1193[M+COOH-HCOOH-C_6_H_10_O_5_]^-^;255.0660[M+COOH-HCOOH-2C_6_H_10_O_5_]^-^;135.0088[M+COOH-HCOOH-2C_6_H_10_O_5_-C_8_H_8_O]^-^ |
| 5 | 5.04 | [M-H]- | C9H6O3 | 161.0245 | 162.0317 | 0.32 | Umbelliferone | 161.0232[M-H]^-^;144.8907[M-H-OH]^-^;117.0348[M-H-CO-OH]^-^；133.0268[M-H-CO]^-^ |
| 6 | 5.24 | [M+COOH]- | C20H18O7 | 415.1030 | 370.1053 | 1.53 | Neouralenol | 415.1080[M+COOH]^-^;295.0624[M-H-C_3_H_4_-2OH]^-^;267.0659[M-H-C_5_H_8_-2OH]^-^;234.9258[M-H-C_7_H_3_O_3_]^-^ |
| 7 | 5.27 | [M-H]- | C27H30O15 | 593.1519 | 594.1585 | 1.23 | Vicenin Ⅱ | 593.1552[M-H]^-^;503.1214[M-H-C_6_H_2_O]^-^;473.1114[M-H-C_7_H_4_O_2_]^-^;413.0891[M-H-C_6_H_10_O_5_-H_2_O]^-^;383.0783[M-H-C_6_H_10_O_5_-CH_3_O-H_2_O]^-^;353.0676[M-H-C_6_H_10_O_5_-CH_3_O-H_2_O-CH_2_O]^-^ |
| 8 | 6.36 | [M+COOH]- | C20H18O7 | 415.1035 | 370.1053 | 2.75 | Gancaonin P | 415.1059[M+COOH]^-^;267.0711[M+COOH-C_5_H_8_-2OH]^-^;252.0421[M+COOH-HCOOH-C_2_H_2_O_2_-C_2_H_2_O-OH]^-^;196.0517[M+COOH-HCOOH-C_8_H_11_O_2_-2OH]^-^;135.0075[M+COOH-HCOOH-C_13_H_13_O_4_-C_7_H_5_O_3_]^-^ |
| 9 | 6.54 | [M+COOH]- | C20H18O7 | 415.1036 | 370.1053 | 2.92 | Uralenol | 415.0985[M+COOH]^-^;252.0423[M+COOH-HCOOH-C_4_H_5_O_4_]^-^;196.0508[M+COOH-HCOOH-C_7_H_9_-2HO]^-^;167.0528[M+COOH-HCOOH-C_7_H_10_-C_6_H_4_O_2_]^-^135.0071[M+COOH-HCOOH-C_12_H_11_O_2_-C_7_H_5_O_3_]^-^ |
| 10 | 6.69 | [M-H]- | C26H30O13 | 549.1618 | 550.1686 | 0.87 | Liguiritigenin-7-O-D-apiosyl-4'-O-D-glucoside | 549.1626[M-H]^-^;429.1050[M-H-C_4_H_8_O_4_]^-^;255.0654[M-H-C_4_H_8_O_4_-C_7_H_10_O_5_]^-^;135.0080[M-H-C_4_H_8_O_4_-C_7_H_10_O_5_-C_8_H_8_O]^-^ |
| 11 | 6.90 | [M+H]+ | C15H12O4 | 257.0806 | 256.0736 | -0.94 | Pinocembrin | 257.0803[M+H]^+^;165.0701[M+H-C_7_H_9_]^+^;137.0235[M+H-C_8_H_8_O]^+^;119.0498[M+H-C_7_H_6_O_3_]^+^;109.0293[M+H-C_8_H_8_O-CO]^+^ |
| 12* | 6.91 | [M-H]- | C21H22O9 | 417.1189 | 418.1264 | -0.48 | Liquiritin | 417.1246[M-H]^-^;255.0677[M-H-C_6_H_10_O_5_]^-^;135.0098[M-H-C_6_H_10_O_5_-C_8_H_8_O]^-^;119.0516[M-H-C_6_H_10_O_5_-C_7_H_4_O_3_]^-^ |
| 13* | 6.95 | [M-H]- | C26H30O13 | 549.1619 | 550.1686 | 0.92 | Liquiritin apioside | 549.1654[M-H]^-^;429.1206[M-H-C_7_H_4_O_2_]^-^;417.1215[M-H-C_5_H_8_O_4_]^-^;399.1103[M-H-C_5_H_8_O_4_-H_2_O]^-^;255.0664[M-H-C_5_H_8_O_4_-C_6_H_10_O_5_]^-^;135.0088[M-H-C_5_H_8_O_4_-C_6_H_10_O_5_-C_8_H_8_O]^-^ |
| 14 | 6.95 | [M+COOH]- | C27H30O15 | 639.1564 | 594.1585 | 1.34 | Biorobin | 639.0573[M+COOH]^-^;507.0143[M+COOH-HCOOH-C_4_H_6_O_2_]^-^;471.0380[M+COOH-HCOOH-C_6_H_2_O_3_]^-^;417.1197[M+COOH-HCOOH-C_7_H_12_O_5_]^-^;309.9566[M+COOH-HCOOH-C_15_H_8_O_6_]^-^ |
| 15 | 9.04 | [M+COOH]- | C27H30O13 | 607.1677 | 562.1686 | 3.27 | Glycyroside | 607.1677[M+COOH]^-^;561.1626[M+COOH-HCOOH]^-^;309.0761[M+COOH-HCOOH-C_16_H_12_O_3_]^-^;267.0662[M+COOH-HCOOH-C_11_H_18_O_9_]^-^;251.0349[M+COOH-HCOOH-C_11_H_18_O_9_-CH_3_]^-^ |
| 16 | 9.23 | [M-H]- | C26H30O13 | 549.1622 | 550.1686 | 1.51 | Isoliquiritin apioside | 549.1641[M-H]^-^;417.1191[M-H-C_5_H_8_O_4_]^-^;255.0663[M-H-C_5_H_8_O_4_-C_6_H_10_O_5_]^-^;135.0085[M-H-C_5_H_8_O_4_-C_6_H_10_O_5_-C_8_H_8_O]^-^;119.0499[M-H-C_5_H_8_O_4_-C_6_H_10_O_5_-C_7_H_4_O_3_]^-^ |
| 17 | 9.30 | [M-H]- | C23H24O10 | 459.1300 | 460.1369 | 0.70 | 6''-O-acetylliquiritin | 459.1323[M-H]^-^;417.1160[M-H-CH_3_-CO]^-^;255.0663[M-H-CH_3_-CO-C_6_H_10_O_5_]^-^;135.0089[M-H-C_2_H_3_O-C_6_H_10_O_5_-C_8_H_8_O]^-^；119.0509[M-H-C_2_H_3_O-C_6_H_10_O_5_-C_8_H_8_O-O]^-^/[M-H-C_2_H_3_O-C_6_H_10_O_5_-C_7_H_4_O_3_]^-^ |
| 18 | 9.31 | [M-H]- | C16H12O4 | 267.0665 | 268.0736 | 0.98 | Formononetin | 267.0691[M-H]^-^;252.0453[M-H-CH_3_]^-^;223.0401[M-H-CH_3_-CHO]^-^;195.0446[M-H-CH_3_-CHO-CO]^-^;135.0103[M-H-CH_3_-CHO-C_7_H_4_]^-^;104.0330[M-H-C_9_H_7_O_3_]^-^ |
| 19* | 9.31 | [M+COOH]- | C22H22O9 | 475.1248 | 430.1264 | 2.73 | Ononin | 475.1248[M+COOH]^-^;267.0671[M+COOH-HCOOH-C_6_H_10_O_5_]^-^;252.0438[M+COOH-HCOOH-C_6_H_10_O_5_-CH_3_]^-^;223.0403[M+COOH-HCOOH-C_6_H_10_O_5_-CO-O]^-^ |
| 20 | 9.49 | [M+H]+ | C17H14O5 | 299.0912 | 298.0841 | -0.53 | Afromosin | 299.0902[M+H]^+^;284.0668[M+H-CH_3_]^+^;267.0647[M+H-CH_4_O]^;^256.0731[M+H-CH_3_-CO]^+^ |
| 21* | 9.52 | [M-H]- | C21H22O9 | 417.1195 | 418.1264 | 0.89 | Isoliquiritin | 417.1189[M-H]^-^;255.0665[M-H-C_6_H_10_O_5_]^-^;148.0168[M-H-C_6_H_10_O_5_-C_6_H_3_O_2_]^-^;135.0092[M-H-C_6_H_10_O_5_-C_8_H_8_O]^-^;119.0511[M-H-C_6_H_10_O_5_-C_7_H_4_O_3_]^-^/[M-H-C_6_H_10_O_5_-C_8_H_8_O-O]^-^ |
| 22 | 9.71 | [M+H]+ | C15H10O4 | 255.0649 | 254.0579 | -1.03 | 7,4'-Dihydroxyflavone | 255.0642[M+H]^+^;237.0528[M+H-CH_4_]^+^;227.0698[M+H-CO]^+^;209.0591[M+H-C_2_H_4_O]^+^;145.0283[M+H-C_6_H_4_O_2_]^+^;137.0234[M+H-C_8_H_6_O]^+^;119.0492[M+H-C_6_H_4_O_2_-CO]^+^;109.0288[M+H-C_8_H_6_O-CO]^+^ |
| 23* | 9.91 | [M-H]- | C15H12O4 | 255.0665 | 256.0736 | 0.96 | Liquiritigenin | 255.0664[M-H]^-^;201.8345[M-H-C_3_H_2_O]^-^;135.0077[M-H-C_8_H_8_O]^-^;119.0497[M-H-C_7_H_4_O_3_]^-^/[M-H-C_8_H_8_O-O]^-^ |
| 24 | 9.99 | [M-H]- | C36H38O16 | 725.2109 | 726.216 | 2.96 | Licorice glycoside A | 725.2167[M-H]^-^;549.1658[M-H-C_10_H_8_O_3_]^-^;417.1206[M-H-C_10_H_8_O_3_-C_5_H_8_O_4_]^-^;255.0665[M-H-C_10_H_8_O_3_-C_5_H_8_O_4_-C_6_H_10_O_5_]^-^ |
| 25 | 10.05 | [M+H]+ | C16H12O5 | 285.0755 | 284.0685 | -0.73 | Calycosin | 285.0742[M+H]^+^;253.0497[M+H-CH_4_O]^+^;242.0576[M+H-C_2_H_3_O]^+^;137.0211[M+H-CH_4_O-C_8_H_3_O]^+^ |
| 26 | 10.15 | [M+H]+ | C16H16O4 | 273.112 | 272.1049 | -0.54 | Vestitol | 273.1120[M+H]^+^;163.0763[M+H-C_6_H_6_O_2_]^+^;148.0535[M+H-C_7_H_9_O_2_]^+^;137.0606[M+H-C_8_H_8_O_2_]^+^;123.0446[M+H-C_9_H_10_O_2_]^+^ |
| 27 | 10.46 | [M+H]+ | C16H12O5 | 285.0755 | 284.0685 | -0.89 | Prunetin | 285.0748[M+H]^+^;270.0523[M+H-CH_3_]^+^;253.0483[M+H-CH_4_O]^+^;225.0538[M+H-CH_4_O-CO]^+^ |
| 28 | 10.54 | [M+H]+ | C16H14O4 | 271.0962 | 270.0892 | -1.17 | Echinatin | 271.0962[M+H]^+^;186.0591[M+H-C_4_H_5_O_2_]^+^;146.0376[M+H-CH_4_O-C_6_H_5_O]^+^;123.0451[M+H-CH_4_O-C_8_H_4_O]^+^ |
| 29 | 11.11 | [M+H]+ | C16H14O4 | 271.0966 | 270.0892 | 0.35 | Medicarpin | 271.0966[M+H]^+^;213.0556[M+H-C_3_H_6_O]^+^;161.0646[M+H-CH_3_O-C_6_H_6_]^+^;147.0399[M+H-CH_3_O-C_6_H_5_O]^+^;137.0591[M+H-C_7_H_6_O-C_2_H_3_]^+^;123.0448[M+H-C_9_H_9_O_2_]^;^109.0643[M+H-C_7_H_6_O-C_2_H_3_-CO]^+^ |
| 30 | 11.42 | [M-H]- | C42H62O17 | 837.3939 | 838.3987 | 3.00 | Macedenosin A | 837.3988[M-H]^-^;661.3639[M-H-C_6_H_8_O_6_]^-^；485.3227[M-H-2C_6_H_8_O_6_]^-^;351.0564[M-H-C_30_H_46_O_5_]^-^;193.0338[M-H-C_30_H_46_O_5_-C_6_H_8_O_5_]^-^;113.0240[M-H-C_30_H_46_O_5_-C_6_H_8_O_5_-CO_2_-2H_2_O]^-^ |
| 31 | 12.05 | [M+H]+ | C30H48O4 | 473.3622 | 472.3553 | -0.72 | 24-hydroxy-11-deoxo-glycyrrhetinic acid methyl ester | 473.3628[M+H]^+^;455.3519[M+H-H_2_O]^+^337.2458[M+H-C_5_H_12_O_4_]^+^;247.1692[M+H-C_14_H_26_O_2_]^+^;201.1617[M+H-C_15_H_28_O_4_]^+^;135.1173[M+H-C_20_H_34_O_4_]^+^ |
| 32 | 12.06 | [M+H]+ | C30H46O3 | 455.3517 | 454.3447 | -0.56 | Glypallidifloric acid | 455.3536[M+H]^+^;437.3418[M+H-H_2_O]^+^;325.2538[M+H-H_2_O-C_7_H_10_O]^+^;301.2161[M+H-C_9_H_14_O_2_]^+^;217.1586[M+H-H_2_O-C_7_H_10_O-C_7_H_12_O]^+^;175.1483[M+H-H_2_O-C_7_H_10_O-C_7_H_12_O-C_3_H_6_]^+^ |
| 33 | 12.26 | [M+H]+ | C21H20O7 | 385.1283 | 384.1209 | 0.41 | Uralenol-3-methylether | 385.1284[M+H]^+^;329.1346[M+H-C_4_H_8_]^+^;243.0658[M+H-C_7_H_11_O_2_-CH_3_O]^+^;229.0849[M+H-C_6_H_5_O_3_-CH_3_O]^+^;137.0257[M+H-C_14_H_17_O_4_]^+^ |
| 34 | 12.47 | [M+H]+ | C16H12O5 | 285.0755 | 284.0685 | -0.92 | Genkwanin | 285.0770[M+H]^+^;253.0500[M+H-CH_4_O]^+^;242.0555[M+H-C_2_H_3_O]^+^;137.0237[M+H-CH_4_O-C_8_H_3_O]^+^ |
| 35 | 12.49 | [M+H]+ | C42H62O18 | 855.4003 | 854.3936 | -0.75 | Dihydroxyglycyrrhetinic acid | 855.3907[M+H]^+^;679.3681[M+H-C_6_H_8_O_6_]^+^;661.3595[M+H-C_6_H_8_O_6_-H_2_O]^+^;643.3466[M+H-C_6_H_8_O_6_-2H_2_O]^+^;485.3249[M+H-C_12_H_18_O_13_]^+^;467.3149[M+H-C_12_H_18_O_13_-H_2_O]^+^;455.3160[M+H-C_12_H_18_O_13_-CH_2_O]^+^ |
| 36 | 12.63 | [M+H]+ | C16H10O6 | 299.0555 | 298.0477 | 1.53 | Glyzaglabrin | 299.0555[M+H]^+^;284.0320[M+H-CH_3_]^+^;271.0590[M+H-CO]^+^;243.0647[M+H-2CO]^+^ |
| 37 | 12.66 | [M+H]+ | C16H14O4 | 271.0965 | 270.0892 | -0.05 | Bolusanthin III | 271.0965[M+H]^+^;239.0738[M+H-CH_4_O]^+^;177.0559[M+H-C_6_H_6_O]^+^;162.0272[M+H-C_6_H_5_O_2_]^+^;121.0300[M+H-CH_4_O-C_8_H_6_O]^+^ |
| 38 | 12.96 | [M+H]+ | C48H74O19 | 955.489 | 954.4824 | -0.74 | Uralsaponin T | 955.4890[M+H]^+^;779.4555[M+H-C_6_H_8_O_6_]^+^;603.4245[M+H-2C_6_H_8_O_6_]^+^;585.4128[M+H-2C_6_H_8_O_6_-H_2_O]^+^；421.3453[M+H-2C_6_H_8_O_6_-H_2_O-C_6_H_12_O_5_]^+^ |
| 39 | 13.06 | [M-H]- | C42H62O16 | 821.3996 | 822.4038 | 3.76 | Glycyrrhizin | 821.4031[M-H]^-^;351.0581[M-H-C_30_H_46_O_4_]^-^；193.0366[M-H-C_30_H_46_O_4_-C_6_H_8_O_5_]^-^ |
| 40 | 13.21 | [M-H]- | C48H72O21 | 983.4532 | 984.4566 | 3.96 | Licorice saponin A3 | 983.4598[M-H]^-^;821.4048[M-H-C_6_H_10_O_5_]^-^;803.3953[M-H-C_6_H_10_O_5_-H_2_O]^-^；351.0581[M-H-C_36_H_56_O_9_]^-^ |
| 41 | 13.25 | [M+H] | C30H46O4 | 471.3463 | 470.3396 | -1.30 | Glycyrrolide Ⅱ | 471.3473[M+H]^+^;453.3380[M+H-H_2_O]^+^;317.2104[M+H-C_9_H_14_O_2_]^+^;299.1977[M+H-C_10_H_20_O_2_]^+^;135.1161[M+H-C_9_H_14_O_2_-C_11_H_18_O_2_]^+^ |
| 42 | 13.69 | [M-H]- | C50H74O22 | 1025.4644 | 1026.4672 | 4.37 | Uralsaponin X | 1025.4690[M-H]^-^;497.1166[M-H-C_32_H_48_O_6_]^-^ |
| 43 | 14.17 | [M+H]+ | C42H60O17 | 837.3901 | 836.3831 | -0.22 | 3-O-[β-D-glu-curonopyranosyl-(1→2)-β-D-glucuronopyranosyl]-24-hydroxy-glabrolide | 837.3823[M+H]^+^;661.3575[M+H-C_6_H_8_O_6_]^+^;643.3477[M+H-C_6_H_8_O_6_-H_2_O]^+^;485.3245[M+H-2C_6_H_8_O_6_]^+^;467.3136[M+H-2C_6_H_8_O_6_-H_2_O_6_]^+^;449.3044[M+H-2C_6_H_8_O_6_-2H_2_O]^+^; 177.0385[M+H-C_36_H_52_O_11_]^+^ |
| 44 | 14.17 | [M+H]+ | C30H44O5 | 485.3258 | 484.3189 | -0.67 | 24-Hydroxyglabrolide | 485.3264[M+H]^+^;409.2729[M+H-CH_4_O-CO_2_]^+^;315.1965[M+H-C_10_H_19_O_2_]^+^;261.1521[M+H-C_14_H_24_O_2_]^+^;187.1471[M+H-CH_4_O-CO_2_-C_14_H_21_O_2_]^+^;187.1471[M+H-C_15_H_23_O_3_]^+^;173.1344[M+H-C_18_H_31_O_4_]^+^ |
| 45 | 14.19 | [M-H]- | C44H64O18 | 879.4049 | 880.4093 | 3.27 | 22β-Acetoxyl-glycyrrhizin | 879.4096[M-H]^-^;703.3753[M-H-C_6_H_8_O_6_]^-^;643.3522[M-H-C_6_H_8_O_6_-C_2_H_4_O_2_]^-^;351.0574[M-H-C_32_H_48_O_6_]^-^;193.0352[M-H-C_32_H_48_O_6_-C_6_H_8_O_5_]^-^;113.0256[M-H-C_32_H_48_O_6_-C_6_H_8_O_5_-CO_2_-2H_2_O]^-^ |
| 46 | 14.23 | [M+H]+ | C30H46O5 | 487.3415 | 486.3345 | -0.63 | 24-Hydroxyglycyrrhetic acid | 487.3400[M+H]^+^;469.3340[M+H-H_2_O]^+^;441.3429[M+H-H_2_O-CO]^+^;423.3233[M+H-2H_2_O-CO]^+^;315.1897[M+H-C_10_H_19_O_2_]^+^;261.1486[M+H-C_14_H_26_O_2_]^+^ |
| 47 | 14.24 | [M+H]+ | C30H44O4 | 469.3306 | 468.324 | -1.27 | Glabrolide | 469.3303[M+H]^+^;451.3204[M+H-H_2_O]^+^;285.1973[M+H-C_9_H_16_O-CO_2_]^+^;261.1494[M+H-C_14_H_24_O]^+^;237.1503[M+H-C_15_H_20_O_2_]^+^;233.1544[M+H-C_15_H_24_O_2_]^+^ |
| 48 | 14.28 | [M+H]+ | C16H10O5 | 283.0598 | 282.0528 | -0.90 | 4'-O-methylcoumestrol | 283.0638[M+H]^+^;253.0495[M+H-CH_2_O]^+^;225.0530[M+H-CH_2_O-CO]^+^;141.0702[M+H-CH_2_O-CO-C_4_H_6_O_2_]^+^ |
| 49 | 14.35 | [M+H]+ | C32H46O5 | 511.3412 | 510.3345 | -1.15 | 3β-Acetyoxyglabrolide | 511.3421[M+H]^+^;451.3214[M+H-C_2_H_4_O_2_]^+^;433.3118[M+H-C_2_H_4_O_3_-H_2_O]^+^;405.3164[M+H-C_3_H_5_O_4_]^+^;189.1640[M+H-C_3_H_5_O_4_-C_16_H_25_]^+^;173.1330[M+H-C_3_H_5_O_4_-C_16_H_25_-CH_4_]^+^ |
| 50 | 14.35 | [M+H]+ | C32H48O6 | 529.3518 | 528.3451 | -1.07 | 22β-Acethylglabric acid | 529.3515[M+H]^+^;511.3421[M+H-H_2_O]^+^;469.3313[M+H-H_2_O-C_2_H_2_O]^+^;451.3207[M+H-H_2_O-C_2_H_2_O-OH]^+^;405.3187[M+H-H_2_O-C_2_H_2_O-2H_2_O-CO]^+^ |
| 51 | 14.77 | [M+H]+ | C30H44O4 | 469.3309 | 468.324 | -0.65 | Isoglabrolide | 469.3304[M+H]^+^;423.3286[M+H-H_2_O-CO]^+^;405.3124[M+H-2H_2_O-CO]^+^317.2090[M+H-C_9_H_13_O_2_]^+^;315.2005[M+H-C_10_H_8_O]^+^;243.1359[M+H-C_9_H_13_O_2_-C_4_H_9_O]^2^37.1470[M+H-C_10_H_8_O-C_5_H_12_O]^+^;233.1560[M+H-C_15_H_24_O_2_]^+^;217.1576[M+H-C_15_H_25_O_2_-CH_3_]^+^ |
| 52 | 14.96 | [M+H]+ | C30H44O5 | 485.3258 | 484.3189 | -0.81 | 21-Hydroxyisoglabrolide | 485.3262[M+H]^+^;409.2668[M+H-CO_2_-CH_3_-OH]^+^;315.1959[M+H-C_9_H_14_O_3_]^+^;301.1787[M+H-C_10_H_16_O_3_]^+^;233.1584[M+H-C_14_H_24_O-CO_2_]^+^ |
| 53 | 14.99 | [M-H]- | C47H72O19 | 939.4627 | 940.4668 | 3.43 | Uralsaponin Q | 939.4695[M-H]^-^;777.4139[M-H-C_6_H_10_O_5_]^-^;627.3515[M-H-C_6_H_10_O_5_-C_5_H_10_O_5_]^-^;485.3255[M-H-C_6_H_10_O_5_-C_5_H_10_O_5_-C_6_H_6_O_4_]^-^ |
| 54* | 15.05 | [M-H]- | C15H12O4 | 255.0663 | 256.0736 | 0.18 | Isoliquiritigenin | 255.0645[M-H]^-^;201.8347[M-H-C_3_H_2_O]^-^;135.0084[M-H-C_8_H_8_O]^-^;119.0505[M-H-C_8_H_8_O-O]^-^/[M-H-C_7_H_4_O_3_]^-^ |
| 55 | 15.31 | [M+COOH]- | C48H72O19 | 997.4683 | 952.4668 | 4.38 | Licorice saponin F3 | 951.4694[M+COOH]^-^;743.4068[M+COOH-HCOOH-C_7_H_13_O_7_]^-^;625.3437[M+COOH-HCOOH-C_7_H_13_O_7_-C_9_H_12_]^-^;247.0773[M+COOH-HCOOH-C_7_H_13_O_7_-C_9_H_12_-C_26_H_24_O_10_]^-^ |
| 56 | 15.45 | [M-H]- | C48H70O20 | 965.4421 | 966.446 | 3.43 | Uralsaponin Y | 965.4485[M-H]^-^;643.3592[M-H-C_12_H_18_O_10_]^-^;497.1149[M-H-C_30_H_44_O_4_]^-^;381.1007[M-H-C_12_H_18_O_10_-C_6_H_8_O_6_-C_5_H_6_O]^-^;321.0823[M-H-C_30_H_44_O_4_-C_6_H_8_O_6_]^-^;175.0240[M-H-C_12_H_18_O_10_-C_30_H_44_O_4_]^-^ |
| 57 | 15.84 | [M-H]- | C42H60O16 | 819.3839 | 820.3881 | 3.69 | Licorice saponin E2 | 819.3878[M-H]^-^;643.3546[M-H-C_6_H_8_O_6_]^-^351.0587[M-H-C_30_H_44_O_4_]^-^;193.0366[M-H-C_30_H_44_O_4_-C_6_H_8_O_5_]^-^;113.0256[M-H-C_30_H_44_O_4_-C_6_H_8_O_5_-CO_2_-2H_2_O]^-^ |
| 58 | 15.89 | [M-H]- | C42H62O17 | 837.3943 | 838.3987 | 3.49 | Licoricesaponin G2 | 837.3989[M-H]^-^;661.3646[M-H-C_6_H_8_O_6_]^-^；351.0577[M-H-C_30_H_46_O_5_]^-^;193.0356[M-H-C_30_H_46_O_5_-C_6_H_8_O_5_]^-^;113.0253[M-H-C_30_H_46_O_5_-C_6_H_8_O_5_-2H_2_O-CO_2_]^-^ |
| 59 | 16.04 | [M+H]+ | C30H44O4 | 469.3307 | 468.324 | -1.25 | Uralenolide | 469.3299[M+H]^+^;423.3258[M+H-H_2_O-CO]^+^;355.2194[M+H-C_6_H_10_O]^+^;311.2005[M+H-C_6_H_10_O-C_3_H_7_O]^;^217.1571[M+H-H_2_O-CO-C_15_H_16_]^+^ |
| 60 | 16.06 | [M+H]+ | C30H46O5 | 487.3413 | 486.3345 | -1.07 | 18-Hydroxyglycyrrhetic acid | 487.3480[M+H]^+^;441.3374[M+H-H_2_O-CO]^+^;423.3268[M+H-2H_2_O-CO]^+^;235.1688[M+H-2H_2_O-CO-C_14_H_20_]^+^;217.1589[M+H-3H_2_O-CO-C_14_H_20_]^+^;189.1640[M+H-3H_2_O-2CO-C_14_H_20_]^+^ |
| 61 | 16.55 | [M+H]+ | C21H20O5 | 353.1381 | 352.1311 | -0.83 | Gancaonin A | 353.1346[M+H]^+^;323.0957[M+H-CO]^+^;297.0701[M+H-C_4_H_6_]^+^;282.0535[M+H-C_5_H_7_]^+^;267.0648[M+H-C_4_H_6_-CH_3_O]^+^;181.0667[M+H-CH_3_O-C_8_H_10_O_2_]^+^;147.0419[M+H-C_12_H_12_O_3_]^+^;107.0494[M+H-C_14_H_12_O_4_]^+^ |
| 62 | 16.69 | [M+H]+ | C30H46O5 | 487.3413 | 486.3345 | -0.95 | Echinatic acid | 487.3400[M+H]^+^;469.3320[M+H-H_2_O]^+^;423.3216[M+H-2H_2_O-CO]^+^;317.2110[M+H-C_9_H_14_O_3_]^+^;251.1643[M+H-C_16_H_28_O]^+^;235.1712[M+H-2H_2_O-CO-C_8_H_10_-C_5_H_7_O]^+^ |
| 63 | 16.80 | [M+H]+ | C32H48O5 | 513.3571 | 512.3502 | -0.62 | Acetylglycyrrhetinic acid | 513.3592[M+H]^+^;453.3423[M+H-C_2_H_4_O_2_]^+^;299.2014[M+H-C_2_H_4_O_2_-C_9_H_14_O_2_]^+^;245.1530[M+H-C_2_H_4_O_2_-C_9_H_14_O_2_-C_4_H_6_]^+^;217.1576[M+H-C_2_H_4_O_2_-C_9_H_14_O_2_-C_4_H_6_-C_2_H_4_]^+^ |
| 64 | 17.07 | [M+H]+ | C30H46O5 | 487.3414 | 486.3345 | -0.76 | Melilotigenin | 487.3395[M+H]^+^;469.3245[M+H-H_2_O]^+^;423.3263[M+H-2H_2_O-CO]^+^;387.3051[M+H-H_2_O-CO-C_4_H_7_]^+^;317.2050[M+H-H_2_O-CO-C_8_H_12_O]^+^;271.2041[M+H-H_2_O-CO-C_8_H_12_O-C_2_H_6_O]^+^ |
| 65 | 17.07 | [M+H]+ | C30H44O4 | 469.3306 | 468.324 | -1.39 | 3-Oxoglycyrrhetinic acid | 469.3265[M+H]^+^;423.3415[M+H-H_2_O-CO]^+^;313.2234[M+H-H_2_O-CO-C_8_H_12_]^+^;299.2006[M+H-H_2_O-CO-C_8_H_12_-CH_4_]^+^;235.1660[M+H-H_2_O-CO-C_14_H_21_]^+^;217.1598[M+H-H_2_O-CO-C_14_H_22_O]^+^ |
| 66* | 17.38 | [M-H]- | C42H62O16 | 821.3994 | 822.4038 | 3.50 | Glycyrrhizic acid | 821.4038[M-H]^-^;645.3691[M-H-C_6_H_8_O_6_]^-^; 627.3572[M-H-C_6_H_8_O_6_-H_2_O]^-^;469.3334[M-H-C_6_H_8_O_6_-C_6_H_8_O_6_]^-^;193.0361[M-H-C_30_H_46_O_4_-C_6_H_8_O_5_]^-^;113.0258[M-H-C_30_H_46_O_4_-C_6_H_8_O_5_-H_2_O-CO_2_-H_2_O]^-^ |
| 67* | 17.53 | [M+H]+ | C42H65NO16 | 840.4362 | 839.4303 | -1.65 | Glycyrrhizic acid ammonium salt | 840.4362[M+H]^+^;647.3775[M+H-C_6_H_8_O_6_-H_3_N]^+^;471.3460[M+H-2C_6_H_8_O_6_-H_3_N]^+^;453.3347[M+H-2C_6_H_8_O_6_-H_3_N-H_2_O]^+^ |
| 68 | 17.53 | [M+H]+ | C30H46O4 | 471.346 | 470.3396 | -1.98 | Methyl 3b,24-dihydroxy-11,13(18)-oleanadien-30-oate | 471.3465[M+H]^+^;453.3386[M+H-H_2_O]^+^;425.3424[M+H-H_2_O-CO]^+^;407.3310[M+H-2H_2_O-CO]^+^；317.2118[M+H-C_9_H_14_O_2_]^+^;313.2162[M+H-C_9_H_17_O_2_]^+^;271.2054[M+H-C_9_H_14_O_2_-H_2_O-CO]^+^ |
| 69 | 17.77 | [M+H]+ | C41H62O14 | 779.4195 | 778.414 | -2.17 | Araboglycyrrhizin | 779.3824[M+H]^+^;471.3460[M+H-C_11_H_16_O_10_]^+^;453.3351[M+H-C_11_H_16_O_10_-H_2_O]^+^;407.3313[M+H-C_11_H_16_O_10_-2H_2_O-CO]^+^;263.1653[M+H-C_11_H_16_O_10_-H_2_O-C_14_H_23_]^+^;217.1573[M+H-C_11_H_16_O_10_-H_2_O-CO-C_14_H_24_O]^+^ |
| 70 | 17.80 | [M+H]+ | C21H20O5 | 353.1385 | 352.1311 | 0.44 | Gancaonin M | 353.1364[M+H]^+^;321.1124[M+H-CH_2_O]^+^;283.0591[M+H-C_5_H_8_]^+^;267.0635[M+H-C_4_H_6_-CH_3_O]^+^;199.0754[M+H-CH_3_O-C_8_H_10_O]^+^;181.0645[M+H-CH_3_O-C_8_H_10_O_2_]^+^;147.0435[M+H-C_12_H_12_O_3_]^+^;134.0329[M+H-C_9_H_7_O_2_-C_5_H_9_]^+^ |
| 71 | 18.20 | [M-H]- | C42H64O15 | 807.4196 | 808.4245 | 2.89 | Licorice saponin B2 | 807.4231[M-H]^-^;789.4133[M-H-H_2_O]^-^;745.4215[M-H-CO_2_-H_2_O]^-^;631.3899[M-H-C_6_H_8_O_6_]^-^;351.0572[M-H-C_30_H_48_O_3_]^-^;193.0357[M-H-C_30_H_48_O_3_-C_6_H_8_O_5_]^-^;113.0259[M-H-C_30_H_48_O_3_-C_6_H_8_O_5_-CO_2_-2H_2_O]^-^ |
| 72 | 18.36 | [M-H]- | C42H62O16 | 821.3997 | 822.4038 | 3.87 | Licorice saponin K2 | 821.4031[M-H]^-^;351.0586[M-H-C_30_H_46_O_4_]^-^;193.0350[M-H-C_30_H_46_O_4_-C_6_H_8_O_5_]^-^ |
| 73 | 18.54 | [M+H]+ | C30H46O4 | 471.346 | 470.3396 | -1.88 | Macedonic acid | 471.3473[M+H]^+^;453.3430[M+H-H_2_O]^+^;425.3362[M+H-H_2_O-CO]^+^;407.3234[M+H-2H_2_O-CO]^+^;329.2181[M+H-C_9_H_18_O]^+^;235.1692[M+H-2H_2_O-CO-C_13_H_17_]^+^ |
| 74 | 19.10 | [M+H]+ | C50H76O21 | 1013.4933 | 1012.4879 | -1.88 | Licorice saponin D3 | 1013.3107[M+H]^+^;867.4397[M+H-C_6_H_10_O_4_]^+^;849.4096[M+H-C_6_H_10_O_4_-H_2_O]^+^;497.3588[M+H-C_18_H_28_O_17_]^+^;437.3406[M-H-C_18_H_28_O_17_-C_2_H_4_O_2_]^+^; |
| 75 | 19.23 | [M+COOH]- | C42H62O16 | 867.4042 | 822.4038 | 3.78 | Uralsaponin B | 821.4021[M+COOH]^-^;645.3664[M+COOH-HCOOH-C_6_H_8_O_6_]^-^;351.0567[M+COOH-HCOOH-C_30_H_44_O_4_]^-^;235.0480[M+COOH-HCOOH-2C_6_H_8_O_6_-C_15_H_22_O_2_]^-^;193.0355[M+COOH-HCOOH-C_30_H_44_O_4_-C_6_H_8_O_5_]^-^;113.0247[M+COOH-HCOOH-C_30_H_44_O_4_-C_6_H_8_O_5_-CO_2_-2H_2_O]^-^ |
| 76 | 19.31 | [M+H]+ | C41H62O14 | 779.4199 | 778.414 | -1.72 | Apioglycyrrhizin | 779.4199[M+H]^+^;471.3431[M+H-C_11_H_16_O_10_]^+^;453.3348[M+H-C_11_H_16_O_10_-H_2_O]^+^;407.3291[M+H-C_11_H_16_O_10_-2H_2_O-CO]^+^;357.2381[M+H-C_11_H_16_O_10_-H_2_O-C_7_H_11_]^+^;217.1567[M+H-C_11_H_16_O_10_-H_2_O-CO-C_14_H_23_O] |
| 77 | 19.56 | [M+COOH]- | C42H64O16 | 869.42 | 824.4194 | 3.93 | Licorice saponin J2 | 823.4206[M+COOH]^-^;647.3875[M+COOH-HCOOH-C_6_H_8_O_6_]^-^;471.3439[M+COOH-HCOOH-2C_6_H_8_O_6_]^-^;351.0584[M+COOH-HCOOH-C_30_H_48_O_2_]^-^;193.0323[M+COOH-HCOOH-C_30_H_48_O_2_-C_6_H_8_O_5_]^-^ |
| 78 | 19.56 | [M-H]- | C21H20O6 | 367.1188 | 368.126 | 0.16 | Glycycoumarin | 367.1182[M-H]^-^;337.0745[M-H-CH_2_O]^-^;309.0405[M-H-C_3_H_5_-OH]^-^;281.0444[M-H-C_5_H_8_-H_2_O]^-^;173.0310[M-H-C_9_H_5_O_4_-OH]^-^;161.0243[M-H-C_12_H_14_O_3_]^-^ |
| 79 | 19.56 | [M+COOH]- | C20H18O4 | 367.1184 | 322.1205 | 2.25 | Licoflavone A | 367.1182[M+COOH]^-^;309.0405[M+COOH-HCOOH-CH]^-^;161.0243[M+COOH-HCOOH-C_11_H_12_O]^-^ |
| 80 | 19.67 | [M+H]+ | C20H16O6 | 353.1017 | 352.0947 | -0.79 | Glycybridin I | 353.1042[M+H]^+^;335.0917[M+H-H_2_O]^+^;311.0543[M+H-C_3_H_6_]^+^;269.0411[M+H-C_5_H_8_O]^+^;243.0639[M+H-C_6_H_6_O_2_]^+^;227.0660[M+H-C_6_H_6_O_3_]^+^;187.0776[M+H-C_8_H_6_O_2_]^+^;165.0194[M+H-C_12_H_12_O_2_]^+^ |
| 81 | 19.95 | [M-H]- | C21H24O5 | 355.1552 | 356.1624 | 0.42 | Medicarpene | 355.1987[M-H]^-^；233.1141[M-H-C_7_H_6_O_2_]^-^;209.0663[M-H-C_6_H_3_O-C_4_H_7_]^-^;195.1076[M-H-C_6_H_3_O-C_4_H_7_-CH_2_]^-^;133.0300[M-H-C_7_H_6_O_2_-C_5_H_9_-CH_3_O]^-^ |
| 82 | 20.18 | [M-H]- | C20H20O5 | 339.1243 | 340.1311 | 1.42 | Glepidotin B | 339.1271[M-H]^-^;245.0853[M-H-C_6_H_4_-H_2_O]^-^;236.1101[M-H-C_5_H_9_-2OH]^-^;219.0660[M-H-C_8_H_8_O]^-^;151.0758[M-H-C_8_H_8_O-C_5_H_8_]^-^ |
| 83 | 20.21 | [M+H]+ | C20H18O6 | 355.1171 | 354.1103 | -1.36 | Gancaonin C | 355.1171[M+H]^+^;299.0538[M+H-C_3_H_2_O]^+^;281.0456[M+H-C_3_H_2_O-H_2_O]^+^;243.0620[M+H-C_3_H_2_O-C_3_H_3_O]^+^;165.0160[M+H-C_8_H_7_O-C_4_H_7_O]^+^ |
| 84 | 20.33 | [M-H]- | C42H62O15 | 805.4042 | 806.4089 | 3.19 | Uralsaponin W | 805.4087[M-H]^-^;629.3728[M-H-C_6_H_8_O_6_]^-^;351.0572[M-H-C_30_H_46_O_3_]^-^;193.0353[M-H-C_30_H_46_O_3_-C_6_H_8_O_5_]^-^;113.0255[M-H-C_30_H_46_O_3_-C_6_H_8_O_5_-CO_2_-2H_2_O]^-^ |
| 85 | 20.35 | [M+COOH]- | C19H16O4 | 353.1023 | 308.1049 | 0.87 | Glycybridin F | 353.1029[M+COOH]^-^;253.0513[M+COOH-HCOOH-C_4_H_6_]^-^;241.0503[M+COOH-HCOOH-C_5_H_6_]^-^;227.0327[M+COOH-HCOOH-C_5_H_4_-O]^-^;107.086[M+COOH-HCOOH-C_5_H_6_O_2_-O-C_8_H_6_O]^-^ |
| 86 | 20.48 | [M+H]+ | C20H18O6 | 355.1173 | 354.1103 | -0.97 | Licoflavonol | 355.1173[M+H]^+^;299.0559[M+H-C_4_H_8_]^+^;269.0406[M+H-C_4_H_8_-CH_2_O]^+^;243.0659[M+H-C_6_H_6_O-H_2_O]^+^;231.0632[M+H-C_8_H_12_O]^+^ |
| 87 | 20.55 | [M+COOH]- | C20H20O4 | 369.1333 | 324.1362 | 0.60 | (E)-1-[2,4-dihydroxy-3-[(2R)-2-hydroxy-3-methylbut-3-enyl]phenyl]-3-phenylprop-2-en-1-one | 369.1326[M+COOH]^-^;309.1133[M+COOH-HCOOH-CH_2_]^-^;267.1008[M+COOH-HCOOH-C_3_H_4_O]^-^;251.0336[M+COOH-HCOOH-C_4_H_8_O]^-^;239.0361[M+COOH-HCOOH-C_5_H_8_O]^-^;149.0584[M+COOH-HCOOH-C_4_H_7_O-C_8_H_7_]^-^ |
| 88 | 20.77 | [M+H]+ | C20H18O6 | 355.1177 | 354.1103 | 0.16 | Licoisoflavanone | 355.1266[M+H]^+^;299.0435[M+H-C_4_H_8_]^+^;271.0571[M+H-C_4_H_8_-CO]^+^;187.0811[M+H-C_6_H_5_O_3_-C_3_H_7_]^+^;179.0327[M+H-C_11_H_12_O_2_]^+^;151.0383[M+H-C_11_H_12_O_2_-C_2_H_4_]^+^ |
| 89 | 20.78 | [M-H]- | C21H22O5 | 353.1387 | 354.1467 | -2.17 | Glycyuralin B | 353.0960[M-H]^-^;295.0639[M-H-C_2_H_3_-CH_3_O]^-^;284.0285[M-H-C_5_H_8_]^-^;254.8531[M-H-C_5_H_8_-CH_2_O]^-^ |
| 90 | 20.97 | [M-H]- | C22H22O6 | 381.1341 | 382.1416 | -0.70 | Licoricone | 381.1327[M-H]^-^;351.0871[M-H-CH_2_O]^-^;323.0560[M-H-CH_2_O-CO]^-^;308.0337[M-H-CH_2_O-CO-CH_3_]^-^;201.0193[M-H-2CH_3_O-C_7_H_10_O_2_]^-^ |
| 91 | 21.09 | [M-H]- | C20H20O5 | 339.1239 | 340.1311 | 0.36 | Glycybridin A | 339.1261[M-H]^-^;324.1014[M-H-CH_3_]^-^;309.0763[M-H-2CH_3_]^-^；281.0446[M-H-2CH_3_-CO]^-^;269.0480[M-H-C_4_H_7_O]^-^;253.0550[M-H-2CH_3_-2CO]^-^;241.0522[M-H-C_4_H_7_O-CO]^-^ |
| 92 | 21.28 | [M+H]+ | C20H18O6 | 355.1173 | 354.1103 | -0.98 | Gancaonin O | 355.1173[M+H]^+^;299.0547[M+H-C_4_H_8_]^+^;281.0426[M+H-C_4_H_8_-H_2_O]^+^;245.0517[M+H-C_6_H_6_O_2_]^+^;153.0173[M+H-C_6_H_6_O_2_-C_7_H_7_]^+^ |
| 93 | 21.41 | [M-H]- | C20H20O5 | 339.1242 | 340.1311 | 1.15 | Cyclolicoflavanone | 339.1222[M-H]^-^;205.1272[M-H-C_7_H_2_O_3_]^-^;187.1128[M-H-C_7_H_2_O_3_-H_2_O]^-^;151.0014[M-H-C_13_H_14_O]^-^;132.0572[M-H-C_7_H_2_O_3_-H_2_O-C_9_H_7_]^-^ |
| 94 | 21.48 | [M-H]- | C21H22O4 | 337.1444 | 338.1518 | -0.26 | 4'-O-methylglabridin | 337.1438[M-H]^-^;305.1150[M-H-CH_4_O]^-^;281.0853[M-H-C_3_H_4_O]^-^;254.8564[M-H-C_5_H_7_O]^-^;237.0577[M-H-C_5_H_7_O-OH]^-^;201.0920[M-H-C_8_H_8_O_2_]^-^;187.0760[M-H-C_8_H_8_O_2_-CH_2_]^-^ |
| 95 | 21.57 | [M-H]- | C42H62O15 | 805.4038 | 806.4089 | 2.79 | Licorice saponin C2 | 805.4069[M-H]^-^;351.0569[M-H-C_30_H_46_O_3_]^-^;193.0347[M-H-C_30_H_46_O_3_-C_6_H_8_O_5_]^-^ |
| 96 | 21.69 | [M+H]+ | C20H18O6 | 355.1173 | 354.1103 | -0.96 | Isolicoflavonol | 355.1159[M+H]^+^;299.0554[M+H-C_4_H_8_]^+^;229.0485[M+H-C_6_H_6_O_3_]^+^;215.0739[M+H-C_6_H_5_O_2_-2CH_3_]^+^;153.0168[M+H-C_13_H_15_O_2_]^+^ |
| 97 | 21.72 | [M-H]- | C20H18O5 | 337.1081 | 338.1154 | -0.10 | Glepidotin A | 337.1094[M-H]^-^;282.0522[M-H-C_4_H_7_]^-^;253.0540[M-H-C_5_H_9_-O]^-^;201.0891[M-H-C_8_H_8_O_2_]^-^;145.0247[M-H-C_8_H_8_O_2_-C_3_H_4_O]^-^;107.0165[M-H-C_8_H_8_O_2_-C_3_H_4_O-C_3_H_2_]^-^ |
| 98 | 21.79 | [M-H]- | C21H18O6 | 365.1032 | 366.1103 | 0.27 | Glycyrol | 365.1040[M-H]^-^;335.0556[M-H-CH_2_O]^-^;307.0247[M-H-CH_2_O-CO]^-^;295.0246[M-H-C_5_H_10_]^-^;282.0164[M-H-CH_2_O-C_4_H_5_]^-^;267.0279[M-H-CH_2_O-C_5_H_9_]^-^ |
| 99 | 21.93 | [M-H]- | C20H18O5 | 337.1084 | 338.1154 | 0.75 | Licoflavone C | 337.1077[M-H]^-^;321.0769[M-H-O]^-^;253.0492[M-H-C_5_H_8_O]^-^;161.0251[M-H-C_11_H_12_O_2_]^-^;133.0661[M-H-C_11_H_12_O_2_-CO]^-^;117.0344[M-H-C_11_H_12_O_2_-CO-O]^-^ |
| 100 | 21.97 | [M-H]- | C21H20O6 | 367.1192 | 368.126 | 1.45 | Gancaonin N | 367.1200[M-H]^-^;309.0435[M-H-C_3_H_5_-HO]^-^;295.0970[M-H-CH_2_O-C_2_H_2_O]^-^;281.0454[M-H-C_5_H_8_-H_2_O]^-^;269.0463[M-H-C_5_H_8_-CH_2_O]^-^ |
| 101 | 22.02 | [M-H]- | C20H20O5 | 339.1238 | 340.1311 | -0.12 | Licoflavanone | 339.2046[M-H]^-^;266.1549[M-H-C_4_H_7_-H_2_O]^-^;205.1409[M-H-C_7_H_2_O_3_]^-^;151.0769[M-H-C_13_H_16_O]^-^;136.0140[M-H-C_13_H_15_O_2_]^-^;133.0664[M-H-C_4_H_7_-H_2_O-C_7_H_2_O_3_]^-^ |
| 102 | 22.17 | [M+H]+ | C20H18O6 | 355.1175 | 354.1103 | -0.42 | Uralenin | 355.1175[M+H]^+^;299.0552[M+H-C_4_H_8_]^+^;254.8677[M+H-C_4_H_8_-H_2_O-CO]^+^;243.0708[M+H-C_7_H_10_O]^+^;215.0773[M+H-C_7_H_10_O-CO]^+^ |
| 103 | 22.23 | [M-H]- | C20H16O6 | 351.0873 | 352.0947 | -0.23 | Licoisoflavone B | 351.0869[M-H]^-^;335.0544[M-H-O]^-^;283.0971[M-H-C_5_H_8_]^-^;175.0388[M-H-C_9_H_4_O_4_-C_9_H_4_O_3_]^-^;159.0421[M-H-C_9_H_6_O_5_-H_2_O]^-^ |
| 104 | 22.31 | [M+COOH]- | C20H18O4 | 367.1186 | 322.1205 | 2.71 | Glabrene | 367.1187[M+COOH]^-^;352.0957[M+COOH-CH_3_]^-^;309.0413[M+COOH-HCOOH-CH]^-^;175.0061[M+COOH-HCOOH-C_9_H_6_O_2_]^-^;134.8950[M+COOH-HCOOH-C_9_H_6_O_2_-C_3_H_5_]^-^ |
| 105 | 22.31 | [M-H]- | C21H20O6 | 367.119 | 368.126 | 0.88 | Gancaonin B | 367.1187[M-H]^-^;337.0689[M-H-CH_2_O]^-^;309.0413[M-H-C_3_H_5_-OH]^-^;281.0463[M-H-C_5_H_8_-H_2_O]^-^;269.0457[M-H-C_5_H_8_-CH_2_O]^-^;175.0061[M-H-C_5_H_8_-CH_2_O-C_5_HO_2_]^-^ |
| 106 | 22.56 | [M-H]- | C20H16O5 | 335.0931 | 336.0998 | 1.85 | Psoralidin | 335.0936[M-H]^-^;319.0614[M-H-O]^-^;305.0450[M-H-O-CH_2_]^-^;245.0937[M-H-O-C_6_H_2_]^-^;205.1613[M-H-O-C_6_H_2_-C_2_HO]^-^;107.0178[M-H-O-C_5_H_10_-C_8_H_2_O-CO]^-^ |
| 107 | 22.56 | [M+COOH]- | C20H20O4 | 369.1341 | 324.1362 | 2.26 | Phaseollinisoflavan | 369.1341[M+COOH]^-^;247.0954[M+COOH-HCOOH-C_3_H_6_O-H_2_O]^-^;232.0765[M+COOH-HCOOH-C_6_H_3_O]^-^;137.0219[M+COOH-HCOOH-C_3_H_6_O-H_2_O-C_9_H_3_]^-^;121.0247[M+COOH-HCOOH-C_6_H_3_O-CH_2_-C_4_H_5_O-C_2_H_4_]^-^ |
| 108 | 22.66 | [M-H]- | C25H28O4 | 391.1921 | 392.1988 | 1.49 | Glabrol | 391.1896[M-H]^-^;345.2058[M-H-2CH_3_-O]^-^;203.0680[M-H-C_13_H_16_O]^-^;187.1147[M-H-C_12_H_12_O_3_]^-^;132.0572[M-H-C_12_H_12_O_3_-C_4_H_7_]^-^ |
| 109 | 22.84 | [M-H]- | C25H28O6 | 423.1811 | 424.1886 | -0.41 | 2-(2-acetoxy-3-methylbut-3-en-1-yl)-5-phenethyl-1,3-phenylenediacetate | 423.1820[M-H]^-^;233.0798[M-H-C_7_H_10_O_6_]^-^;193.0858[M-H-C_7_H_10_O_6_-C_3_H_4_]^-^;149.0960[M-H-C_7_H_10_O_6_-C_3_H_4_-COOH]^-^ |
| 110 | 22.86 | [M+H]+ | C26H32O5 | 425.2312 | 424.225 | -2.47 | Licoricidin | 425.3980[M+H]^+^;313.0689[M+H-C_8_H_16_]^+^;221.1159[M+H-C_8_H_16_-C_3_H_8_O_3_]^+^;135.0440[M+H-C_8_H_16_-C_3_H_8_O_3_-C_7_H_2_]^+^ |
| 111 | 22.87 | [M-H]- | C22H24O6 | 383.15 | 384.1573 | -0.06 | Glycyuralin D | 383.2276[M-H]^-^;355.1533[M-H-C_2_H_4_]^-^;340.1320[M-H-C_2_H_4_-CH_3_]^-^;281.1182[M-H-C_2_H_4_-CH_3_-C_2_H_3_O_2_]^-^ |
| 112 | 23.03 | [M-H]- | C21H22O5 | 353.1397 | 354.1467 | 0.62 | Licobenzofuran | 353.1026[M-H]^-^;323.0917[M-H-CH_2_O]^-^;268.0372[M-H-C_5_H_8_-OH]^-^;192.8936[M-H-CH_2_O-C_10_H_10_]^-^;160.8407[M-H-C_10_H_8_O_4_]^-^ |
| 113 | 23.04 | [M+COOH]- | C20H20O3 | 353.1396 | 308.1412 | 3.55 | Isocordoin | 353.1026[M+COOH]^-^-;338.1168[M+COOH-CH_3_]^-^;268.0372[M+COOH-HCOOH-C_3_H_3_]^-^;173.0244[M+COOH-HCOOH-C_3_H_3_-C_5_H_3_O_2_]^-^;116.9245[M+COOH-HCOOH-C_3_H_3_-C_5_H_3_O_2_-C_4_H_9_]^-^ |
| 114* | 23.49 | [M+H]+ | C30H46O4 | 471.3459 | 470.3396 | -2.18 | Glycyrrhetinic acid | 471.3440[M+H]^+^;425.3359[M+H-H_2_O-CO]^+^;407.3328[M+H-2H_2_O-CO]^+^;317.2091[M+H-C_10_H_17_O]^+^;217.1503[M+H-H_2_O-CO-C_14_H_24_O]^+^;135.1154[M+H-C_10_H_17_O-C_11_H_18_O_2_]^+^ |
| 115 | 24.55 | [M-H]- | C30H48O3 | 455.352 | 456.3603 | -2.36 | Oleanolicacid | 455.3565[M-H]^-^;409.2464[M-H-H_2_O-CO]^-^;391.2343[M-H-2H_2_O-CO]^-^;373.2236[M-H-C_6_H_10_]^-^;355.2097[M-H-C_6_H_10_-H_2_O]^-^;273.7719[M-H-C_6_H_10_-C_5_H_7_O_2_]^-^ |

Note: * Meant that the compounds are identified with reference standards.

| **Table S3** Identification of prototypes in rat biosamples after oral administration of ZGC by UPLC-Q-TOF-MS. | | | | | | | | | | | | | | | | | | |  |
| --- | --- | --- | --- | --- | --- | --- | --- | --- | --- | --- | --- | --- | --- | --- | --- | --- | --- | --- | --- |
| **NO.** | **RT**  **(min)** | **Selected ion** | **Formula** | **Mass (Da)** | **Measured ion** | **Error (ppm)** | **Identification** | **MS/MS fragmentation** | **Source** | | | | | | | | | |  |
|  |  |  |  |  |  |  |  |  | **S** | **H** | **L** | | **K** | | **F** | | **U** | |  |
| P1 | 4.50 | [M+COOH]^-^ | C_27_H_32_O_14_ | 580.1792 | 625.1767 | 0.59 | Liquiritigenin-7,4'-diglucoside | 625.2786[M+COOH]^-^;  417.1196[M+COOH-HCOOH-C_6_H_10_O_5_]^-^;  255.0659[M+COOH-HCOOH-2C_6_H_10_O_5_]^-^;  135.0093[M+COOH-HCOOH-2C_6_H_10_O_5_-C_8_H_8_O]^-^ |  |  | |  | |  | |  | | √ | |
| P2 | 4.88 | [M-H]^-^ | C_9_H_6_O_3_ | 162.0317 | 161.0245 | 0.25 | Umbelliferone | 161.0276[M-H]^-^;  144.8955[M-H-OH]^-^;  133.0320[M-H-CO]^-^;  117.0360[M-H-CO-OH]^-^;  105.0370[M-H-2CO]^-^ |  |  | |  | | √ | |  | | √ | |
| P3 | 5.16 | [M-H]^-^ | C_27_H_30_O_15_ | 594.1585 | 593.1497 | -2.49 | Vicenin Ⅱ | 593.1552[M-H]^-^;  503.1193[M-H-C_6_H_2_O]^-^;  473.1093[M-H-C_7_H_4_O_2_]^-^;  413.0924[M-H-C_6_H_10_O_5_-H_2_O]^-^;  383.0777[M-H-C_6_H_10_O_5_-CH_3_O-H_2_O]^-^;  353.0664[M-H-C_6_H_10_O_5_-CH_3_O-H_2_O-CH_2_O]^-^ |  |  | |  | | √ | | √ | |  | |
| P4* | 6.75 | [M-H]^-^ | C_21_H_22_O_9_ | 418.1264 | 417.1193 | 0.40 | Liquiritin | 417.1203[M-H]^-^;  255.0664[M-H-C_6_H_10_O_5_]^-^;  135.0096[M-H-C_6_H_10_O_5_-C_8_H_8_O]^-^;  119.0516[M-H-C_6_H_10_O_5_-C_7_H_4_O_3_]^-^; 119.0516[M-H-C_6_H_10_O_5_-C_8_H_8_O-O]^-^ | √ | √ | |  | |  | | √ | | √ | |
| P5 | 6.77 | [M-H]^-^ | C_26_H_30_O_13_ | 550.1686 | 549.1613 | -0.08 | Liguiritigenin-7-O-D-apiosyl-4'-O-D-glucoside | 549.1626[M-H]^-^;  429.1196[M-H-C_7_H_4_O_2_]^-^;  255.0664[M-H-C_5_H_8_O_4_-C_6_H_10_O_5_]^-^;  135.0089[M-H-C_5_H_8_O_4_-C_6_H_10_O_5_-C_8_H_8_O]^-^ | √ |  | |  | |  | |  | |  | |
| P6* | 6.88 | [M-H]^-^ | C_26_H_30_O_13_ | 550.1686 | 549.1617 | 0.56 | Liquiritin apioside | 549.1600[M-H]^-^;  417.1198[M-H-C_5_H_8_O_4_]^-^;  255.0648[M-H-C_5_H_8_O_4_-C_6_H_10_O_5_]^-^;  135.0077[M-H-C_5_H_8_O_4_-C_14_H_17_O_6_]^-^ | √ | √ | | √ | | √ | |  | |  | |
| P7 | 7.10 | [M+H]^+^ | C_15_H_12_O_4_ | 256.0736 | 257.0801 | -3.01 | Pinocembrin | 257.0803[M+H]^+^;  242.0553[M+H-O]^+^;  137.0234[M+H-C_8_H_8_O]^＋^;  119.0494[M+H-C_8_H_8_O-H_2_O]^＋^ | √ | √ | |  | |  | | √ | | √ | |
| P8 | 9.23 | [M-H]^-^ | C_26_H_30_O_13_ | 550.1686 | 549.1633 | 3.56 | Isoliquiritin apioside | 549.1631[M-H]^-^;  503.2688[M-H-H_2_O-CO]^-^;429.2037[M-H-C_7_H_4_O_2_]^-^;413.2190[M-H-C_7_H_4_O_3_]^-^;  255.0644[M-H-C_5_H_8_O_4_-C_6_H_10_O_5_]^-^;  119.0506[M-H-C_5_H_8_O_4_-C_6_H_10_O_5_-C_7_H_4_O_3_]^-^ | √ |  | |  | |  | |  | | √ | |
| P9* | 9.31 | [M+COOH]^-^ | C_22_H_22_O_9_ | 430.1264 | 475.1248 | 2.73 | Ononin | 475.1538[M+COOH]^+^;  267.0671[M+COOH-HCOOH-C_6_H_10_O_5_]^-^;  252.0429[M+COOH-HCOOH-C_6_H_10_O_5_-CH_3_]^-^ | √ |  | |  | |  | | √ | | √ | |
| P10* | 9.39 | [M-H]^-^ | C_21_H_22_O_9_ | 418.1264 | 417.1188 | -0.68 | Isoliquiritin | 417.1955[M-H]^-^;  255.071[M-H-C_6_H_10_O_5_]^-^;  148.0167[M-H-C_6_H_10_O_5_-C_6_H_3_O_2_]^-^;  135.0093[M-H-C_6_H_10_O_5_-C_8_H_8_O]^-^;  119.0528[M-H-C_6_H_10_O_5_-C_7_H_4_O_3_]^-^ | √ |  | |  | |  | | √ | | √ | |
| P11 | 9.73 | [M+H]^+^ | C_15_H_10_O_4_ | 254.0579 | 255.0644 | -2.92 | 7,4'-Dihydroxyflavone | 255.0642[M+H]^+^;  237.0545[M+H-CH_4_]^＋^;  227.0700[M+H-CO]^+^;  209.0605[M+H-C_2_H_4_O]^+^;  145.0278[M+H-C_6_H_4_O_2_]^+^;  137.0230[M+H-C_8_H_6_O]^+^;  119.0494[M+H-C_6_H_4_O_2_-CO]^+^;  109.0293[M+H-C_8_H_6_O-CO]^+^ | √ |  | | √ | | √ | | √ | | √ | |
| P12* | 9.73 | [M-H]^-^ | C_15_H_12_O_4_ | 256.0736 | 255.0667 | 1.52 | Liquiritigenin | 255.0693[M-H]^-^;  135.0107[M-H-C_8_H_8_O]^-^;  119.0526[M-H-C_7_H_4_O_3_]^-^ | √ | √ | | √ | | √ | | √ | | √ | |
| P13 | 10.09 | [M+H]^+^ | C_16_H_12_O_5_ | 284.0685 | 285.0752 | -1.84 | Calycosin | 285.0755[M+H]^+^;  253.0496[M+H-CH_4_O]^+^;  242.0572[M+H-C_2_H_3_O]^+^;  137.0240[M+H-CH_4_O-C_8_H_3_O]^+^;  107.0505[M+H-C_10_H_8_O_3_]^+^ |  |  | |  | |  | |  | | √ | |
| P14 | 10.48 | [M+H]^+^ | C_16_H_12_O_5_ | 284.0685 | 285.0751 | -2.34 | Prunetin | 285.0748[M+H]^+^;  270.0512[M+H-CH_3_]^+^;  253.0489[M+H-CH_4_O]^+^;  225.0538[M+H-CH_4_O-CO]^＋^ |  |  | |  | |  | |  | | √ | |
| P15 | 10.54 | [M+H]^+^ | C_16_H_16_O_4_ | 272.1049 | 273.1107 | -5.30 | Vestitol | 273.1107[M+H]^+^;  163.0739[M+H-C_6_H_6_O_2_]^＋^;  123.0435[M+H-C_9_H_10_O_2_]^＋^;  103.0537[M+H-C_8_H_10_O_4_]^＋^ | √ |  | |  | |  | |  | |  | |
| P16 | 11.91 | [M+H]^+^ | C_30_H_46_O_3_ | 454.3447 | 455.3524 | 0.96 | Glypallidifloric acid | 455.3531[M+H]^+^;  437.3423[M+H-H_2_O]^＋^;  325.2534[M+H-H_2_O-C_7_H_10_O]^＋^;  217.1589[M+H-H_2_O-C_7_H_10_O-C_7_H_12_O]^＋^;  175.1481[M+H-H_2_O-C_7_H_10_O-C_7_H_12_O-C_3_H_6_]^＋^ |  |  | |  | |  | | √ | |  | |
| P17 | 12.51 | [M+H]^+^ | C_16_H_12_O_5_ | 284.0685 | 285.0752 | -1.90 | Genkwanin | 285.0758[M+H]^+^;  267.0651[M+H-H_2_O]^+^;  253.0494[M+H-CH_4_O]^+^;  242.0569[M+H-C_2_H_3_O]^+^;  121.0648[M+H-C_9_H_8_O_3_]^+^ |  |  | |  | |  | | √ | | √ | |
| P18 | 12.62 | [M+H]^+^ | C_16_H_14_O_4_ | 270.0892 | 271.0966 | 0.31 | Bolusanthin III | 271.0622[M+H]^+^;  229.0926[M+H-C_2_H_2_O]^+^;  197.0606[M+H-C_2_H_2_O-CH_3_O]^+^;  177.0503[M+H-C_6_H_6_O]^+^;  162.0203[M+H-C_6_H_5_O_2_]^+^;  121.0302[M+H-CH_4_O-C_8_H_6_O]^+^;  107.0506[M+H-CH_3_O-C_9_H_8_O]^+^ |  |  | |  | |  | | √ | |  | |
| P19 | 12.63 | [M+H]^+^ | C_16_H_10_O_6_ | 298.0477 | 299.0550 | -0.19 | Glyzaglabrin | 299.0546[M+H]^+^;  284.0319[M+H-CH_3_]^＋^;  271.0601[M+H-CO]^＋^;  243.0652[M+H-2CO]^＋^ |  |  | |  | |  | | √ | | √ | |
| P20 | 12.83 | [M+H]^+^ | C_48_H_74_O_19_ | 954.4824 | 955.4897 | -0.02 | Uralsaponin T | 955.4769[M+H]^+^;  779.4573[M+H-C_6_H_8_O_6_]^＋^;  603.4258[M+H-2C_6_H_8_O_6_]^＋^;  585.4135[M+H-2C_6_H_8_O_6_-H_2_O]^＋^ |  |  | |  | |  | | √ | |  | |
| P21 | 12.89 | [M-H]^-^ | C_42_H_62_O_16_ | 822.4038 | 821.3971 | 0.72 | Glycyrrhizin | 821.4005[M-H]^-^;  351.0571[M-H-C_30_H_46_O_4_]^-^;  193.0359[M-H-C_30_H_46_O_4_-C_6_H_8_O_5_]^-^ |  |  | |  | |  | | √ | |  | |
| P22 | 13.23 | [M-H]^-^ | C_48_H_72_O_21_ | 984.4566 | 983.4535 | 4.23 | Licorice saponin A3 | 983.4586[M-H]^-^;  821.4054[M-H-C_6_H_10_O_5_]^-^;  803.3912[M-H-C_6_H_10_O_5_-H_2_O]^-^ ;  351.0570[M-H-C_36_H_56_O_9_]^-^ | √ |  | |  | |  | | √ | | √ | |
| P23 | 13.87 | [M-H]^-^ | C_50_H_74_O_22_ | 1026.4672 | 1025.4638 | 3.85 | Uralsaponins X | 1025.4660[M-H]^-^;  497.1199[M-H-C_32_H_48_O_6_]^-^;  435.1200[M-H-C_18_H_27_O_7_-C_2_H_3_O_2_-H_2_O]^-^;  321.0944[M-H-C_32_H_48_O_6_-C_6_H_10_O_6_]^-^ |  |  | |  | | √ | |  | |  | |
| P24 | 13.98 | [M+H]^+^ | C_42_H_60_O_17_ | 836.3831 | 837.3910 | 0.75 | 3-O-[β-D-glu-curonopyranosyl-(1→2)-β-D-glucuronopyranosyl]^-^24-hydroxy-glabrolide | 837.3910[M+H]^+^;  467.3131[M+H-2C_6_H_8_O_6_-H_2_O]^+^;  449.3038[M+H-2C_6_H_8_O_6_-2H_2_O]^+^ |  |  | |  | | √ | | √ | |  | |
| P25 | 14.00 | [M-H]^-^ | C_44_H_64_O_18_ | 880.4093 | 879.4021 | 0.15 | 22β-Acetoxyl-glycyrrhizin | 879.4047[M-H]^-^;  819.3849[M-H-C_2_H_4_O_2_]^-^;  703.3724[M-H-C_6_H_8_O_6_]^-^;  643.3500[M-H-C_6_H_8_O_6_-C_2_H_4_O_2_]^-^;  351.0561[M-H-C_32_H_48_O_6_]^-^;  193.0353[M-H-C_32_H_48_O_6_-C_6_H_8_O_5_]^-^;  113.0257[M-H-C_32_H_48_O_6_-C_6_H_8_O_5_-2H_2_O-CO_2_]^-^ | √ | √ | |  | | √ | | √ | | √ | |
| P26 | 14.07 | [M+H]^+^ | C_30_H_44_O_4_ | 468.3240 | 469.3311 | -0.21 | Glabrolide | 469.3321[M+H]^+^;  451.3211[M+H-H_2_O]^+^;  237.1511[M+H-C_15_H_20_O_2_]^+^;  233.1537[M+H-C_15_H_24_O_2_]^+^;  187.1484[M+H-C_15_H_26_O_2_-CO_2_]^+^ |  |  | |  | |  | | √ | |  | |
| P27 | 14.22 | [M+H]^+^ | C_32_H_46_O_5_ | 510.3345 | 511.3422 | 0.76 | 3β-Acetyoxyglabrolide | 511.3429[M+H]^+^;  405.3166[M+H-C_3_H_5_O_4_]^＋^;  189.1647[M+H-C_3_H_5_O_4_-C_16_H_25_]^＋^;  173.1333[M+H-C_3_H_5_O_4_-C_16_H_25_-CH_4_]^＋^ |  |  | |  | |  | | √ | |  | |
| P28 | 14.34 | [M+H]^+^ | C_16_H_10_O_5_ | 282.0528 | 283.0592 | -3.06 | 4'-O-methylcoumestrol | 283.0604[M+H]^+^;  253.0496[M+H-CH_2_O]^＋^;  225.0546[M+H-CH_2_O-CO]^＋^;  141.0702[M+H-CH_2_O-CO-C_4_H_6_O_2_]^＋^ |  |  | |  | |  | |  | | √ | |
| P29* | 14.83 | [M-H]^-^ | C_15_H_12_O_4_ | 256.0736 | 255.0671 | 3.26 | Isoliquiritigenin | 255.0668[M-H]^-^;  201.8355[M-H-C3H2O]^-^;  135.0103[M-H-C8H8O]^-^;  119.0523[M-H-C8H8O-O]^-^/[M-H-C7H4O3]^-^ | √ |  | |  | |  | | √ | | √ | |
| P30 | 14.95 | [M-H]^-^ | C_47_H_72_O_19_ | 940.4668 | 939.4601 | 0.68 | Uralsaponin Q | 939.3554[M-H]^-^;  777.4254[M-H-C_6_H_10_O_5_]^-^;  715.4058[M-H-C_7_H_12_O_8_]^-^;  627.3433[M-H-C_6_H_10_O_5_-C_5_H_10_O_5_]^-^ | √ |  | |  | |  | | √ | |  | |
| P31 | 15.60 | [M-H]^-^ | C_42_H_60_O_16_ | 820.3881 | 819.3817 | 0.99 | Licorice saponin E2 | 819.3866[M-H]^-^;  643.3536[M-H- C_6_H_8_O_6_]^-^;  351.0578[M-H-C_30_H_44_O_4_]^-^;  193.0360[M-H-C_30_H_44_O_4_-C_6_H_8_O_5_]^-^;  113.0261[M-H-C_30_H_44_O_4_-C_6_H_8_O_5_-CO_2_-2H_2_O]^-^ |  |  | |  | | √ | | √ | |  | |
| P32 | 15.67 | [M-H]^-^ | C_42_H_62_O_17_ | 838.3987 | 837.3923 | 1.05 | Licoricesaponin G2 | 837.3923[M-H]^-^;  661.3630[M-H-C_6_H_8_O_6_]^-^;  351.0577[M-H-C_30_H_46_O_5_]^-^;  193.0363[M-H-C_30_H_46_O_5_-C_6_H_8_O_5_]^-^;  113.0263[M-H-C_30_H_46_O_5_-C_6_H_8_O_5_-H_2_O-CO_2_-H_2_O]^-^ | √ |  | |  | | √ | | √ | |  | |
| P33 | 15.85 | [M+H]^+^ | C_30_H_44_O_4_ | 468.3240 | 469.3312 | -0.08 | Uralenolide | 469.3304[M+H]^+^;  451.3200[M+H-H_2_O]^+^;  423.3251[M+H-H_2_O-CO]^＋^;  217.1582[M+H-H_2_O-CO-C_15_H_16_]^＋^;  189.1626[MH-H_2_O-CO-C_15_H_16_-CO]^＋^ |  |  | |  | |  | | √ | |  | |
| P34 | 15.85 | [M+H]^+^ | C_30_H_46_O_5_ | 486.3345 | 487.3415 | -0.62 | 18-Hydroxyglycyrrhetic acid | 487.3422[M+H]^+^;  441.3365[M+H-H_2_O-CO]^＋^;  423.3267[M+H-H_2_O-CO-H_2_O]^＋^;  235.1700[M+H-H_2_O-CO-H_2_O-C_14_H_20_]^＋^;  217.1592[M+H-H_2_O-CO-H_2_O-C_14_H_20_-H_2_O]^＋^;  189.1648[M+H-H_2_O-CO-H_2_O-C_14_H_20_-H_2_O-CO]^＋^ |  |  | |  | |  | | √ | |  | |
| P35 | 16.60 | [M+H]^+^ | C_21_H_20_O_5_ | 352.1311 | 353.1382 | -0.42 | Gancaonin A | 353.1048[M+H]^+^;  320.0681[M+H-CH_3_O]^+^;  283.0572[M+H-C_5_H_8_]^+^;  199.0822[M+H-CH_3_O-C_8_H_10_O]^+^;  147.0453[M+H-C_12_H_12_O_3_]^+^;  135.0435[M+H-C_9_H_7_O_2_-C_5_H_9_]^+^ |  |  | |  | |  | | √ | |  | |
| P36 | 16.63 | [M+H]^+^ | C_32_H_48_O_5_ | 512.3502 | 513.3570 | -0.85 | Acetylglycyrrhetinic acid | 513.3597[M+H]^+^;453.3378[M+H-C_2_H_4_O_2_]^＋^;  299.2014[M+H-C_2_H_4_O_2_-C_9_H_14_O_2_]^＋^;  245.1547[M+H-C_2_H_4_O_2_-C_9_H_14_O_2_-C_4_H_6_] ^＋^;  217.1591[M+H-C_2_H_4_O_2_-C_9_H_14_O_2_-C_4_H_6_-C_2_H_4_]^＋^ |  |  | |  | |  | | √ | |  | |
| P37* | 17.19 | [M-H]^-^ | C_42_H_62_O_16_ | 822.4038 | 821.3971 | 0.68 | Glycyrrhizic acid | 821.3971[M-H]^-^;  645.3651[M-H-C_6_H_8_O_6_]^-^;  627.3550[M-H-C_6_H_8_O_6_-H2O]^-^;  351.0551[M-H-C_30_H_46_O_4_]^-^;  193.0350[M-H-C_30_H_46_O_4_-C_6_H_8_O_5_]^-^;  113.0254[M-H-C_30_H_46_O_4_-C_6_H_8_O_5_-H_2_O-CO_2_-H_2_O]^-^ | √ | √ | |  | | √ | | √ | | √ | |
| P38 | 17.38 | [M+H]^+^ | C_30_H_46_O_4_ | 470.3396 | 471.3466 | -0.55 | Methyl 3b,24-dihydroxy-11,13(18)-  oleanadien-30-oate | 471.3465[M+H]^+^;  453.3358[M+H-H_2_O]^＋^;  425.3417[M+H-H_2_O-CO]^＋^;  407.3319[M+H-2H_2_O-CO]^＋^;  317.2121[M+H-H_2_O-CO-C_10_H_17_O]^＋^;  313.2177[M+H-C_9_H_14_O_2_ H_2_O-CO]^＋^ |  |  | |  | |  | | √ | |  | |
| P39* | 17.46 | [M+H]^+^ | C_42_H_65_NO_16_ | 839.4303 | 840.4369 | -0.89 | Glycyrrhizic acid ammonium salt | 840.4369[M+H]^+^;  647.3780[M+H-C_6_H_8_O_6_ -H_3_N]^+^;  471.3462[M+H-2C_6_H_8_O_6_-H_3_N]^+^;  453.3348[M+H-2C_6_H_8_O_6_-H_3_N-H_2_O]^+^ | √ | √ | | √ | | √ | | √ | |  | |
| P40 | 17.79 | [M+H]^+^ | C_21_H_20_O_5_ | 352.1311 | 353.1380 | -0.92 | Gancaonin M | 353.1382[M+H]^+^;  297.0763[M+H-C_4_H_8_]^+^;  283.0606[M+H-C_5_H_8_]^＋^;  226.0640[M+H-C_5_H_10_-C_2_HO_2_]^＋^;  147.0448[M+H-C_12_H_12_O_3_]^＋^ |  |  | |  | |  | | √ | |  | |
| P41 | 18.03 | [M-H]^-^ | C_42_H_64_O_15_ | 808.4245 | 807.4176 | 0.42 | Licorice saponin B2 | 807.4217[M-H]^-^;  631.3888[M-H- C_6_H_8_O_6_]^-^;  455.3562[M-H-C_12_H_16_O_12_]^-^;  351.0582[M-H-C_30_H_48_O_3_]^-^;  193.0362[M-H-C_30_H_48_O_3_-C_6_H_8_O_5_]^-^;  113.0261[M-H-C_30_H_48_O_3_-C_6_H_8_O_5_-CO_2_-2H_2_O]^-^ |  |  | | √ | |  | |  | |  | |
| P42 | 18.18 | [M-H]^-^ | C_42_H_62_O_16_ | 822.4038 | 821.3973 | 0.99 | Licorice saponin K2 | 821.3973[M-H]^-^;  351.0569[M-H-C_30_H_46_O_4_]^-^;  193.0366[M-H-C_30_H_46_O_4_-C_6_H_8_O_5_]^-^ |  |  | |  | |  | | √ | |  | |
| P43 | 19.19 | [M+H]^+^ | C_41_H_62_O_14_ | 778.4140 | 779.4205 | -0.96 | Apioglycyrrhizin | 779.4205[M+H]^+^;  471.3491[M+H-C_11_H_16_O_10_]^+^;  453.3379[M+H-C_11_H_16_O_10_-H_2_O]^+^ |  |  | |  | |  | | √ | |  | |
| P44 | 19.39 | [M+COOH]^-^ | C_42_H_64_O_16_ | 824.4194 | 869.4170 | 0.56 | Licorice saponin J2 | 869.4199[M+COOH]^-^;  823.4153[M+COOH-HCOOH]^-^;  351.0573[M+COOH-HCOOH-C_30_H_48_O_2_]^-^ | √ |  | |  | |  | |  | |  | |
| P45 | 19.39 | [M-H]^-^ | C_21_H_20_O_6_ | 368.1260 | 367.1180 | -1.84 | Glycycoumarin | 367.1199[M-H]^-^;  337.0726[M-H-CH_2_O]^-^;  351.0878[M-H-CH_3_]^-^;  309.0409[M-H-CH_3_-C_3_H_7_]^-^;  297.0413[M-H-CH_3_-C_4_H_7_]^-^ |  |  | |  | |  | | √ | |  | |
| P46 | 19.39 | [M+COOH]^-^ | C_20_H_18_O_4_ | 322.1205 | 367.1179 | 0.67 | Licoflavone A | 367.1199[M+COOH]^-^;  309.0409[M+COOH-HCOOH-CH]^-^;  161.0245[M+COOH-HCOOH-CH-C_10_H_11_O]^-^ |  |  | |  | |  | | √ | |  | |
| P47 | 19.59 | [M+H]^+^ | C_20_H_16_O_6_ | 352.0947 | 353.1018 | -0.56 | Glycybridin Ⅰ | 353.1019[M+H]^+^;  335.0910[M+H-H_2_O]^＋^;  269.0433[M+H-C_5_H_8_O]^＋^;  243.0659[M+H-C_6_H_6_O_2_]^＋^;  227.0703[M+H-C_7_H_10_O_2_]^＋^;  155.0843[M+H-C_9_H_10_O_5_]^＋^ |  |  | |  | |  | | √ | | √ | |
| P48 | 19.99 | [M-H]^-^ | C_20_H_20_O_5_ | 340.1311 | 339.1235 | -0.77 | Glepidotin B | 339.1260[M-H]^-^;  297.1161[M-H-C_3_H_6_]^-^;  245.0806[M-H-C_6_H_4_-H_2_O]^-^;  233.0856[M-H-C_7_H_6_O]^-^;  219.0672[M-H-C_8_H_8_O]^-^;  176.0103[M-H-C_9_H_7_O_3_]^-^;  163.0031[M-H-C_11_H_11_O_2_]^-^;  151.0789[M-H-C_8_H_8_O-C_5_H_8_]^-^ |  |  | |  | |  | | √ | |  | |
| P49 | 20.21 | [M-H]^-^ | C_42_H_62_O_15_ | 806.4089 | 805.4021 | 0.60 | Uralsaponin W | 805.4043[M-H]^-^;  629.3720[M-H-C_6_H_8_O_6_]^-^;  453.3387[M-H-C_6_H_8_O_6_-C_6_H_7_O_6_]^-^;  351.0570[M-H-C_30_H_46_O_3_]^-^;  193.0357[M-H-C_30_H_46_O_3_-C_6_H_8_O_5_]^-^ |  |  | |  | |  | | √ | |  | |
| P50 | 20.28 | [M+COOH]^-^ | C_19_H_16_O_4_ | 308.1049 | 353.1022 | 0.78 | Glycybridin F | 353.1049[M+COOH]^-^;  241.0504[M+COOH-HCOOH-C_5_H_6_]^-^;  227.0346[M+COOH-HCOOH-C_5_H_4_-O]^-^;  107.0141[M+COOH-HCOOH-C_5_H_6_O_2_-O-C_8_H_6_O]^-^ |  |  | |  | |  | | √ | |  | |
| P51 | 20.37 | [M+H]^+^ | C_20_H_18_O_6_ | 354.1103 | 355.1177 | 0.25 | Licoflavonol | 355.2651[M+H]^+^;  299.0559[M+H-C_4_H_8_]^+^;  243.0656[M+H-C_6_H_6_O-H_2_O]^＋^;  231.0653[M+H-C_8_H_12_O]^＋^ |  |  | |  | |  | | √ | | √ | |
| P52 | 20.63 | [M+COOH]^-^ | C_20_H_20_O_4_ | 324.1362 | 369.1330 | -0.69 | Glepidotin CⅡ | 369.1367[M+COOH]^-^;  309.1158[M+COOH-HCOOH-CH_2_]^-^;  251.0355[M+COOH-HCOOH-C_4_H_8_O]^-^;  149.0222[M+COOH-HCOOH-C_4_H_7_O-C_8_H_7_]^-^ |  |  | |  | |  | | √ | |  | |
| P53 | 20.81 | [M-H]^-^ | C_22_H_22_O_6_ | 382.1416 | 381.1342 | -0.39 | Licoricone | 381.1348[M-H]^-^;  351.0871[M-H-CH_2_O]^-^;  323.0565[M-H-CH_2_O-CO]^-^;  308.0328[M-H-CH_2_O-CO-CH_3_]^-^;  201.0183[M-H-2CH_3_O-C_7_H_10_O_2_]^-^ | √ |  | |  | |  | | √ | | √ | |
| P54 | 20.90 | [M-H]^-^ | C_20_H_20_O_5_ | 340.1311 | 339.1231 | -2.10 | Glycybridin A | 339.1247[M-H]^-^;  324.1014[M-H-CH_3_]^-^;  309.0779[M-H-2CH_3_]^-^;  281.0461[M-H-2CH_3_-CO]^-^;  269.0462[M-H-C_4_H_7_O]^-^;  253.0507[M-H-2CH_3_-2CO]^-^;  241.0494[M-H-C_4_H_7_O-CO]^-^ |  |  | |  | |  | | √ | |  | |
| P55 | 21.20 | [M+H]^+^ | C_20_H_18_O_6_ | 354.1103 | 355.1173 | -0.75 | Gancaonin O | 355.1173[M+H]^+^;  299.0556[M+H-C_4_H_8_]^+^;  281.0455[M+H-C_4_H_8_-H_2_O]^+^;  245.0449[M+H-C_6_H_6_O_2_]^+^;  153.0194[M+H-C_6_H_6_O_2_-C_7_H_7_]^+^ |  |  | |  | |  | |  | | √ | |
| P56 | 21.29 | [M-H]^-^ | C_20_H_20_O_5_ | 340.1311 | 339.1232 | -1.67 | Cyclolicoflavanone | 339.1240[M-H]^-^;  281.0473[M-H-C_3_H_6_O]^-^;  187.1135[M-H-C_7_H_2_O_3_-H_2_O]^-^;  151.0053[M-H-C_13_H_14_O]^-^;  132.0584[M-H-C_7_H_2_O_3_-H_2_O-C_9_H_7_]^-^ |  |  | |  | |  | | √ | |  | |
| P57 | 21.50 | [M-H]^-^ | C_42_H_62_O_15_ | 806.4089 | 805.4019 | 0.33 | Licorice saponin C2 | 805.4045[M-H]^-^;  629.3734[M-H-C_6_H_8_O_6_]^-^;  351.0557[M-H-C_30_H_46_O_3_]^-^;  193.0326[M-H-C_30_H_46_O_3_-C_6_H_8_O_5_]^-^ |  |  | |  | |  | | √ | |  | |
| P58 | 21.62 | [M+H]^+^ | C_20_H_18_O_6_ | 354.1103 | 355.1177 | 0.18 | Isolicoflavonol | 355.1208[M+H]^+^;  299.0563[M+H-C_4_H_8_]^+^;  229.0504[M+H-C_6_H_6_O_3_]^+^;  215.0695[M+H-C_6_H_5_O_2_-2CH_3_]^+^;  153.0173[M+H-C_13_H_15_O_2_]^+^ |  |  | |  | |  | | √ | |  | |
| P59 | 21.73 | [M-H]^-^ | C_21_H_18_O_6_ | 366.1103 | 365.1019 | -3.10 | Glycyrol | 365.1045[M-H]^-^;  335.0573[M-H-CH_2_O]^-^;  307.0256[M-H-CH_3_-C_3_H_7_]^-^;  295.0253[M-H-CH_3_-C_4_H_7_]^-^ |  |  | |  | |  | | √ | |  | |
| P60 | 22.11 | [M-H]^-^ | C_20_H_18_O_6_ | 354.1103 | 355.1173 | -0.86 | Uralenin | 355.2697[M+H]^+^;  299.0573[M+H-C_4_H_8_]^+^;  243.0667[M+H-C_7_H_10_O]^+^;  215.0691[M+H-C_7_H_10_O-CO]^+^ |  |  | |  | |  | | √ | |  | |
| P61 | 22.17 | [M-H]^-^ | C_20_H_16_O_6_ | 352.0947 | 351.0866 | -2.44 | Licoisoflavone B | 351.0894[M-H]^-^;  335.0571[M-H-O]^-^;  283.0988[M-H-C_5_H_8_]^-^;  175.0411[M-H-C_9_H_4_O_4_-C_9_H_4_O_3_]^-^ |  |  | |  | |  | | √ | |  | |
| P62 | 22.27 | [M-H]^-^ | C_21_H_20_O_6_ | 368.1260 | 367.1179 | -2.18 | Gancaonin B | 367.1186[M-H]^-^;  309.0401[M-H-C_3_H_5_-HO]^-^;  281.0444[M-H-C_5_H_8_-H_2_O]^-^;  269.0447[M-H-C_5_H_8_-CH_2_O]^-^;  175.0047[M-H-C_5_H_8_-CH_2_O-C_5_HO_2_]^-^ |  |  | |  | |  | | √ | |  | |
| P63 | 22.27 | [M+COOH]^-^ | C_20_H_18_O_4_ | 322.1205 | 367.1175 | -0.27 | Glabrene | 367.1186[M+COOH]^-^;  309.0401[M+COOH-HCOOH-CH]^-^;  175.0047[M+COOH-HCOOH-C_9_H_6_O_2_]^-^ |  |  | |  | |  | | √ | |  | |
| P64 | 22.50 | [M+COOH]^-^ | C_20_H_20_O_4_ | 324.1362 | 369.1329 | -0.93 | Phaseollinisoflavan | 369.0909[M+COOH]^-^;  247.1014[M+COOH-HCOOH-C_3_H_6_O-H_2_O]^-^;  232.0754[M+COOH-HCOOH-C_6_H_3_O]^-^;  137.0235[M+COOH-HCOOH-C_3_H_6_O-H_2_O-C_9_H_3_]^-^;  121.0287[M+COOH-HCOOH-C_6_H_3_O-CH_2_-C_4_H_5_O-C_2_H_4_]^-^ |  |  | |  | |  | | √ | |  | |
| P65 | 22.52 | [M-H]^-^ | C_20_H_16_O_5_ | 336.0998 | 335.0918 | -2.18 | Psoralidin | 335.0941[M-H]^-^;  319.0626[M-H-O]^-^;  305.0467[M-H-O-CH_2_]^-^;  135.0081[M-H-O-C_5_H_10_-C_8_H_2_O]^-^;  107.0143[M-H-O-C_5_H_10_-C_8_H_2_O-CO]^-^ |  |  | |  | |  | | √ | |  | |
| P66 | 22.82 | [M-H]^-^ | C_25_H_28_O_6_ | 424.1886 | 423.1803 | -2.46 | 2-(2-acetoxy-3-methylbut-3-en-1-yl)-5-phenethyl-1,3-phenylene diacetate | 423.1804[M-H]^-^;  339.0867[M-H-2C_2_H_3_O]^-^;  233.0812[M-H-C_7_H_10_O_6_]^-^;  193.0862[M-H-C_7_H_10_O_6_-C_3_H_4_]^-^;  149.0966[M-H-C_7_H_10_O_6_-C_3_H_4_-COOH]^-^ |  |  | |  | |  | | √ | |  | |
| P67 | 22.82 | [M+H]^+^ | C_26_H_32_O_5_ | 424.2250 | 425.2322 | -0.11 | Licoricidin | 425.3147[M+H]^+^;  313.1080[M+H-C_8_H_16_]^＋^;  221.1176[M+H-C_8_H_16_-C_3_H_8_O_3_]^＋^;  135.0455[M+H-C_8_H_16_-C_3_H_8_O_3_-C_7_H_2_]^＋^ |  |  | |  | |  | | √ | |  | |
| P68 | 23.01 | [M+COOH]^-^ | C_20_H_20_O_3_ | 308.1412 | 353.1387 | 0.93 | Glepidotin CⅠ | 353.1416[M+COOH]^-^;  268.0385[M+COOH-HCOOH-C_3_H_3_]^-^;  173.0242[M+COOH-HCOOH-C_3_H_3_-C_5_H_3_O_2_]^-^ |  |  | |  | |  | | √ | |  | |
| P69 | 23.01 | [M-H]^-^ | C_21_H_22_O_5_ | 354.1467 | 353.1387 | -2.09 | Licobenzofuran | 353.1416[M-H]^-^;  323.0944[M-H-CH_2_O]^-^;  268.0385[M-H-C_5_H_8_-HO]^-^;  159.0449[M-H-C_10_H_9_O_4_]^-^ |  |  | |  | |  | | √ | |  | |
| P70* | 23.45 | [M+H]^+^ | C_30_H_46_O_4_ | 470.3396 | 471.3466 | -0.69 | Glycyrrhetinic acid | 471.3459[M+H]^+^;  425.3419[M+H-H_2_O-CO]^＋^;  407.3315[M+H-H_2_O-H_2_O-CO]^＋^;  317.2101[M+H-C_10_H_17_O]^+^;  217.1587[M+H-H_2_O-CO-C_14_H_24_O]^＋^;  135.1158[M+H-C_10_H_17_O-C_11_H_18_O_2_]^+^ | √ | √ | | √ | | √ | | √ | | √ | |

**Table S4** Identification of metabolites in rat biosamples after oral administration of ZGC by UPLC-Q-TOF-MS.

| **NO.** | **RT**  **(min)** | **Selected ion** | **Formula** | **Mass (Da)** | **Measured**  **ion** | **Error (ppm)** | **Identification** | **MS/MS fragmentation** | **Source** | | | | | |
| --- | --- | --- | --- | --- | --- | --- | --- | --- | --- | --- | --- | --- | --- | --- |
|  |  |  |  |  |  |  |  |  | **S** | **H** | **L** | **K** | **F** | **U** |
| M1 | 4.19 | [M-H]^-^ | C_27_H_28_O_16_ | 608.1377 | 607.1309 | 0.75 | Liquiritigenin+2GluA | 607.1336[M-H]^-^;  431.0995[M-H-GluA]^-^;  413.0892[M-H-GluA-H_2_O]^-^;  255.0657[M-H-2GluA]^-^;  135.0089[M-H-GluA-C_8_H_8_O]^-^ | √ |  |  |  |  | √ |
| M2 | 4.56 | [M-H]^-^ | C_27_H_30_O_15_ | 594.1588 | 593.1515 | 0.60 | Liquiritin apioside-apiose+GluA | 593.1515[M-H]^-^;  432.1003[M-H-C_6_H_9_O_5_]^-^;  417.1184[M-H-GluA]^-^;  255.0665[M-H-GluA-C_6_H_10_O_5_]^-^;  176.0268[M-H-C_21_H_21_O_9_]^-^;  135.0096[M-H-GluA-C_6_H_10_O_5_-C_8_H_8_O]^-^ | √ |  |  |  |  | √ |
| M3 | 4.80 | [M-H]^-^ | C_32_H_38_O_19_ | 726.2007 | 725.1941 | 0.82 | Liquiritin apioside+GluA | 725.1992[M-H]^-^;  549.1634[M-H-GluA]^-^;  417.1187[M-H-GluA-C_5_H_8_O_4_]^-^;  297.0788[M-H-GluA-C_5_H_8_O_4_-C_7_H_4_O_2_]^-^;  255.0663[M-H-GluA-C_5_H_8_O_4_-C_6_H_10_O_5_]^-^;  135.0109[M-H-GluA-C_5_H_8_O_4_-C_6_H_10_O_5_-C_8_H_8_O]^-^ |  |  |  |  |  | √ |
| M4 | 5.22 | [M-H]^-^ | C_21_H_22_O_12_S | 498.0834 | 497.0761 | 0.30 | Liquiritin+Sul | 497.0768[M-H]^-^;  417.1199[M-H-SO_3_]^-^;  255.0663[M-H-SO_3_-C_6_H_10_O_5_]^-^;  135.0095[M-H-SO_3_-C_6_H_10_O_5_-C_8_H_8_O]^-^;  119.0513[M-H-SO_3_-C_6_H_10_O_5_-C_7_H_4_O_3_]^-^ | √ |  |  |  |  | √ |
| M5 | 5.24 | [M+H]^+^ | C_27_H_30_O_15_ | 594.1585 | 595.1651 | -1.16 | Liquiritin+GluA | 595.2582[M+H]^+^;  457.1095[M+H-C_7_H_4_O_2_-H_2_O]^+^;  325.0718[M+H-GluA-C_6_H_5_O]^+^;  294.0495[M+H-C_6_H_5_O-GluA-CH_3_O]^+^ |  |  |  |  | √ | √ |
| M6 | 5.26 | [M-H]^-^ | C_21_H_22_O_11_ | 450.1159 | 449.1086 | -0.70 | Liquiritin apioside-apiose+2O | 449.1879[M-H]^-^;327.0748[M-H-C_6_H_2_O_3_]^-^;  315.0841[M-H-C_7_H_2_O_3_]^-^;  255.0657[M-H-C_6_H_8_O_5_-2H_2_O]^-^;  135.0087[M-H-C_6_H_8_O_5_-2H_2_O-C_8_H_8_O]^-^;  119.0515[M-H-C_6_H_8_O_5_-2H_2_O-C_7_H_4_O_3_]^-^ |  |  |  |  |  | √ |
| M7 | 5.27 | [M+H]^+^ | C_21_H_20_O_9_ | 416.1107 | 417.1177 | -0.69 | Liquiritin-2H | 417.2554[M+H]^+^;  147.0439[M+H-GluA-C_6_H_6_O]^+^;  131.0490[M+H-GluA-C_6_H_4_O_2_]^+^;  107.0496[M+H-GluA-C_9_H_8_O]^+^ |  |  |  |  |  | √ |
| M8 | 5.33 | [M-H]^-^ | C_26_H_30_O_16_S | 630.1255 | 629.1188 | 0.92 | Liquiritin apioside+Sul | 629.1203[M-H]^-^;  549.1627[M-H-SO_3_]^-^;  417.1199[M-H-SO_3_-C_5_H_8_O_4_]^-^ ;  297.0777[M-H-SO_3_-C_5_H_8_O_4_-C_7_H_4_O_2_]^-^;  255.0664[M-H-SO_3_-C_5_H_8_O_4_-C_6_H_9_O_5_]^-^;  135.0094[M-H-SO_3_-C_5_H_8_O_4_-C_6_H_9_O_5_-C_8_H_8_O]^-^ |  |  |  | √ |  | √ |
| M9 | 5.69 | [M+H]^+^ | C_27_H_32_O_15_ | 596.1738 | 597.1811 | -0.60 | Liquiritin+2H+GluA | 597.2146[M+H]^+^;  579.2281[M+H-H_2_O]^+^;  268.0919[M+H-GluA-C_8_H_9_O_3_]^+^;  259.0941[M+H-GluA-C_6_H_10_O_5_]^+^;  153.0553[M+H-GluA-C_6_H_10_O_5_-C_7_H_6_O]^+^ |  |  |  |  |  | √ |
| M10 | 5.92 | [M+H]^+^ | C_26_H_28_O_14_ | 564.1479 | 565.1547 | -0.87 | Liquiritin apioside+O-2H | 565.2249[M+H]^+^;  427.1043[M+H-C_7_H_6_O_3_]^+^;  409.0927[M+H-C_7_H_6_O_3_-H_2_O]^+^;  295.0605[M+H-C_15_H_10_O_5_]^+^ |  |  |  |  | √ | √ |
| M11 | 6.21 | [M-H]^-^ | C_26_H_32_O_12_ | 536.1893 | 535.1820 | -0.10 | Liquiritin apioside-O+2H | 535.1838[M-H]^-^;  505.1702[M-H-CH_2_O]^-^;  487.1603[M-H-CH_2_O-H_2_O]^-^;  443.1725[M-H-C_6_H_4_O]^-^;  427.1793[M-H-CH_2_O-C_6_H_6_]^-^;  298.0480[M-H-C_5_H_8_O_4_-C_7_H_5_O]^-^ |  |  |  |  |  | √ |
| M12 | 6.27 | [M-H]^-^ | C_27_H_32_O_14_ | 580.1792 | 579.1714 | -0.93 | Liquiritin apioside+O+CH_2_ | 579.1704[M-H]^-^;  441.1213[M-H-C_6_H_4_O_2_-CH_3_O]^-^;  255.0669[M-H-C_11_H_18_O_10_-CH_2_]^-^;  135.0081[M-H-C_11_H_18_O_9_-C_9_H_10_O_2_]^-^ |  |  |  |  | √ |  |
| M13 | 6.41 | [M+COOH]^-^ | C_21_H_24_O_8_ | 404.1466 | 449.1448 | -1.10 | Liquiritin-O+2H | 449.1442[M+COOH]^-^;  255.1027[M+COOH-HCOOH-C_9_H_8_O_2_]^-^;  240.0791[M+COOH-HCOOH-C_6_H_9_O_5_] ^-^;  223.0756[M+COOH-HCOOH-C_9_H_8_O_2_-CH_3_OH-]^-^;  133.0294[M+COOH-HCOOH-C_9_H_8_O_2_-CH_4_O-C_6_H_4_O]^-^ |  |  |  |  |  | √ |
| M14 | 6.48 | [M-H]^-^ | C_32_H_38_O_19_ | 726.2007 | 725.1947 | 1.70 | Liquiritin apioside+GluA+iso | 725.1962[M-H]^-^;  549.1621[M-H-GluA]^-^;  417.1191[M-H-GluA-C_5_H_8_O_4_]^-^;  297.0771[M-H-GluA-C_5_H_8_O_4_-C_7_H_4_O_2_]^-^;  255.0659[M-H-GluA-C_5_H_8_O_4_-C_6_H_10_O_5_]^-^ |  |  |  |  |  | √ |
| M15 | 6.49 | [M+COOH]^-^ | C_22_H_22_O_10_ | 446.1211 | 491.1193 | -0.30 | Liquiritin-Glu+CH_2_+GluA | 491.1193[M+COOH]^-^;  445.1889[M+COOH-HCOOH]^-^;  415.1842[M-H-CH_2_-O]^-^;  315.0881[M+COOH-GluA]^-^;  287.0923[M+COOH-GluA-CO]^-^;  273.0757[M+COOH-GluA-CO-CH_2_]^-^193.0129[M+COOH-HCOOH-C_16_H_12_O_3_]^-^ |  |  |  |  |  | √ |
| M16 | 6.49 | [M-H]^-^ | C_27_H_30_O_15_ | 594.1592 | 593.1519 | 1.20 | Liquiritin apioside-apiose+GluA+iso | 593.1522[M-H]^-^;  431.0969[M-H-C_9_H_6_O_3_]^-^;  417.1196[M-H-GluA]^-^;  255.0665[M-H-GluA-C_6_H_11_O_5_]^-^ |  |  |  |  |  | √ |
| M17 | 6.58 | [M-H]^-^ | C_21_H_20_O_10_ | 432.1056 | 431.0981 | -0.69 | Liquiritin apioside-apiose-Glu+Glu A | 431.0994[M-H]^-^;255.0664[M-H-GluA]^-^;  135.0094[M-H-GluA-C_8_H_8_O]^-^;  119.0513[M-H-GluA-C_7_H_4_O_3_]^-^ | √ | √ |  | √ |  | √ |
| M18 | 6.76 | [M+H]^+^ | C_27_H_30_O_15_ | 594.1585 | 595.1654 | -0.66 | Liquiritin+GluA+iso | 595.3146[M+H]^+^;  433.1106[M+H-C_6_H_10_O_5_]^+^;  256.2723[M+H-C_6_H_11_O_5_-GluA]^+^;  239.0699[M+H-C_6_H_10_O_5_-GluA-H_2_O]^+^;  137.0231[M+H-C_6_H_10_O_5_-GluA-C_8_H_8_O]^+^ | √ |  |  |  |  | √ |
| M19 | 6.78 | [M+H]^+^ | C_27_H_30_O_14_ | 578.1636 | 579.1705 | -0.52 | Liquiritin-O+Glu A | 579.2108[M+H]^+^;  441.1179[M+H-C_7_H_5_O_2_-HO]^+^;  325.0695[M+H-GluA-C_6_H_5_]^+^;  295.0597[M+H-GluA-C_7_H_8_O]^+^ |  |  |  |  | √ |  |
| M20 | 6.85 | [M-H]^-^ | C_21_H_20_O_10_ | 432.1056 | 431.0980 | -0.94 | Liquiritin apioside-apiose-Glu+Glu A | 431.0993[M-H]^-^;  297.0777[M-H-C_7_H_2_O_3_]^-^;  268.0735[M-H-C_9_H_7_O_3_]^-^;  255.0663[M-H-GluA]^-^;  213.0554[M-H-GluA-C_2_H_2_O]^-^;  135.0097[M-H-GluA-C_8_H_8_O]^-^;  119.0517[M-H-GluA-C_7_H_4_O_3_]^-^ | √ | √ |  | √ |  | √ |
| M21 | 7.01 | [M-H]^-^ | C_15_H_12_O_8_S | 352.0257 | 351.0184 | 1.10 | Isoliquiritigenin+O+Sul | 351.0192[M-H-SO_3_]^-^;  253.0510[M-H-SO_3_-H_2_O]^-^;  161.0251[M-H-SO_3_-C_6_H_6_O_2_]^-^;  135.0441[M-H-SO_3_-C_7_H_4_O_3_]^-^;  107.0516[M-H-SO_3_-C_9_H_8_O_3_]^-^ |  |  |  |  |  | √ |
| M22 | 7.13 | [M-H]^-^ | C_21_H_22_O_10_ | 434.1213 | 433.1139 | -0.35 | Liquiritin apioside-apiose+O | 433.1152[M-H]^-^;  299.0932[M-H-C_7_H_2_O_3_]^-^;  223.0614[M-H-C_9_H_7_O_4_-CH_3_O]^-^;  163.0415[M-H-C_15_H_10_O_15_]^-^;  151.0419[M-H-C_6_H_10_O_5_-C_8_H_8_O]^-^;  135.0110[M-H-C_6_H_10_O_5_-C_7_H_4_O_3_]^-^;  107.0532[M-H-C_6_H_10_O_5_-C_9_H_8_O_3_]^-^ |  |  |  |  |  | √ |
| M23 | 7.24 | [M+COOH]^-^ | C_21_H_20_O_9_ | 416.1111 | 461.1093 | 0.80 | Liquiritin-2H | 461.1864[M+COOH]^-^;  255.0297[M+COOH-HCOOH-C_9_H_4_O_3_]^-^;  135.0082[M+COOH-HCOOH-C_9_H_4_O_3_-C_8_H_8_O]^-^;  134.0356[M+COOH-HCOOH-C_7_H_2_O_2_-C_6_H_11_O_5_]^-^;  117.0191[M+COOH-HCOOH-C_7_H_2_O_2_-C_6_H_11_O_6_]^-^ | √ |  |  |  |  |  |
| M24 | 7.28 | [M+H]^+^ | C_15_H_12_O_3_ | 240.0786 | 241.0849 | -4.19 | Liquiritigenin-O | 241.0849[M+H]^+^;  223.0762[M+H-H_2_O]^+^;  198.0677[M+H-C_2_H_3_O]^+^;  147.0444[M+H-C_6_H_6_O]^+^;  131.0496[M+H-C_6_H_6_O_2_]^+^;  119.0499[M+H-C_7_H_6_O_2_]^+^;  107.0498[M+H-C_9_H_10_O]^+^ |  |  |  |  |  | √ |
| M25 | 7.28 | [M-H]^-^ | C_15_H_12_O_7_S | 336.0304 | 335.0232 | 0.18 | Liquiritigenin+Sul | 335.0240[M-H]^-^;  255.0662[M-H-SO_3_]^-^;  213.0560[M-H-SO_3_-C_2_H_2_O]^-^;  135.0099[M-H-SO_3_-C_8_H_8_O]^-^;  119.0520[M-H-SO_3_-C_8_H_8_O_2_]^-^/[M-H-SO_3_-C_7_H_4_O_3_]^-^ |  |  |  | √ | √ | √ |
| M26 | 7.33 | [M-H]^-^ | C_21_H_22_O_12_S | 498.0832 | 497.0759 | -0.07 | Liquiritin apioside-apiose+Sul+iso | 497.0731[M-H]^-^;  417.1182[M-H-SO_3_]^-^;  297.0758[M-H-SO_3_-C_7_H_4_O_2_]^-^;  255.0656[M-H-SO_3_-C_6_H_10_O_5_]^-^;  135.0081[M-H-SO_3_-C_6_H_10_O_5_-C_8_H_8_O]^-^;  119.0506[M-H-SO_3_-C_6_H_10_O_5_-C_7_H_4_O_3_]^-^ |  |  |  |  |  | √ |
| M27 | 7.36 | [M+H]^+^ | C_21_H_22_O_10_ | 434.1213 | 435.1282 | -0.82 | Liquiritin+O | 435.1249[M+H]^+^;  153.0546[M+H-C_6_H_11_O_6_-C_8_H_7_]^+^;  131.0491[M+H-C_6_H_11_O_6_-C_6_H_5_O_3_]^+^;  107.0498[M+H-C_6_H_11_O_6_-C_9_H_9_O_2_]^+^ |  |  |  |  |  | √ |
| M28 | 7.36 | [M+H]^+^ | C_15_H_14_O_4_ | 258.0892 | 259.0958 | -2.56 | Liquiritigenin+2H | 259.1384[M+H]^+^;  153.0553[M+H-C_7_H_6_O]^+^;  135.0447[M+H-C_7_H_8_O_2_]^+^;  107.0503[M+H-C_9_H_12_O_2_]^+^ |  |  |  |  |  | √ |
| M29 | 7.60 | [M-H]^-^ | C_26_H_30_O_12_ | 534.1737 | 533.1663 | -0.20 | Liquiritin apioside-O | 533.1656[M-H]^-^;  443.1715[M-H-C_6_H_2_O]^-^;  413.0904[M-H-C_7_H_4_O_2_]^-^;  357.1326[M-H-C_9_H_5_O_2_-CH_3_O]^-^ |  |  |  |  |  | √ |
| M30 | 7.70 | [M-H]^-^ | C_15_H_12_O_8_S | 352.0253 | 351.0179 | -0.25 | Liquiritigenin+O+Sul | 351.0169[M-H]^-^;  271.0585[M-H-SO_3_]^-^;  253.0475[M-H-SO_3_-H_2_O]^-^;  135.0441[M-H-SO_3_-C_7_H_4_O_3_]^-^;  121.0244[M-H-SO_3_-C_8_H_6_O_3_]^-^ | √ |  |  |  |  |  |
| M31 | 7.83 | [M+COOH]^-^ | C_17_H_14_O_4_ | 282.0892 | 327.0869 | 1.66 | Liquiritin-Glu+C_2_H_2_O-O | 327.0871[M+COOH]^-^;  239.1078[M+COOH-HCOOH-C_2_H_2_O]^-^;  130.0436[M+COOH-HCOOH-C_6_H_4_O-C_2_H_3_O_2_]^-^;  119.0511[M+COOH-HCOOH-C_7_H_3_O-C_2_H_3_O_2_]^-^ |  |  |  |  | √ |  |
| M32 | 7.85 | [M+COOH]^-^ | C_21_H_24_O_9_ | 420.1420 | 465.1392 | 0.12 | Liquiritin+2H | 465.1421[M+COOH]^-^;  389.2015[M+COOH-HCOOH-CH_2_O]^-^;  256.0740[M+COOH-HCOOH-CH_2_O-C_5_H_9_O_4_]^-^;  163.0762[M+COOH-HCOOH-C_15_H_12_O_4_]^-^ |  |  |  |  |  | √ |
| M33 | 8.11 | [M-H]^-^ | C_21_H_20_O_11_ | 448.1004 | 447.0931 | -0.30 | Liquiritin apioside-apiose-Glu+O+GluA | 447.0931[M-H]^-^;  271.0622[M-H-GluA]^-^;  177.0199[M-H-C_15_H_10_O_5_]^-^;1  51.0049[M-H-GluA-C_8_H_7_O]^-^;  119.0516[M-H-GluA-C_7_H_4_O_4_]^-^;  107.0154[M-H-GluA-C_9_H_8_O_3_]^-^ |  |  |  |  |  | √ |
| M34 | 8.25 | [M-H]^-^ | C_15_H_14_O_5_ | 274.0842 | 273.0769 | 0.40 | Liquiritin-Glu+2H+O | 273.0769[M-H]^-^;  255.0606[M-H-H_2_O]^-^;  151.0405[M-H-C_7_H_6_O_2_]^-^;  135.0095[M-H-H_2_O-C_8_H_8_O]^-^ |  |  |  | √ |  |  |
| M35 | 8.25 | [M-H]^-^ | C_15_H_10_O_5_ | 270.0528 | 269.0457 | 0.56 | Liquiritin-Glu+O-2H | 269.0465[M-H]^-^;  135.0095[M-H-C_8_H_6_O_2_]^-^;  133.0308[M-H-C_7_H_3_O_2_-HO]^-^;  115.0190[M-H-C_7_H_3_O_2_-HO-H_2_O]^-^ |  |  |  |  | √ |  |
| M36 | 8.30 | [M+H]^+^ | C_15_H_12_O_5_ | 272.0676 | 273.0749 | -3.20 | Liquiritigenin+O | 273.0782[M+H]^+^;  255.1034[M+H-H_2_O]^+^;  163.0382[M+H-C_6_H_5_O_2_]^+^;  147.0454[M+H-C_6_H_6_O_3_]^+^;  135.0446[M+H-C_8_H_8_O]^+^ |  |  |  |  | √ | √ |
| M37 | 8.39 | [M+COOH]^-^ | C_23_H_24_O_9_ | 444.1420 | 489.1395 | 0.80 | Liquiritin-O+C_2_H_2_O | 489.1411[M+COOH]^-^;  311.0919[M+COOH-HCOOH-C_8_H_4_O_2_]^-^;  269.1200[M+COOH-HCOOH-C_8_H_4_O_2_-C_2_H_2_O]^-^;  121.0311[M+COOH-HCOOH-C_8_H_4_O_2_-C_2_H_2_O-C_6_H_12_O_4_]^-^ |  |  |  |  |  | √ |
| M38 | 8.51 | [M+H]^+^ | C_21_H_20_O_11_ | 448.1006 | 449.1074 | -1.00 | Isoliquiritin-Glu+O+GluA | 449.2785[M+H]^+^;  272.2219[M+H-GluA]^+^;  153.0181[M+H-GluA-C_6_H_6_O-C_2_H_2_]^+^;  147.0440[M+H-GluA-C_6_H_6_O_3_]^+^;  119.0490[M+H-GluA-C_7_H_5_O_4_]^+^ | √ |  |  |  |  | √ |
| M39 | 8.56 | [M-H]^-^ | C_15_H_14_O_5_ | 274.0841 | 273.0771 | 0.94 | Liquiritin-Glu+2H+O | 273.0735[M-H]^-^;  181.0647[M-H-C_6_H_4_O]^-^;  149.0254[M-H-C_6_H_3_O_2_-HO]^-^;  136.0178[M-H-C_7_H_5_O_3_]^-^;  121.0311[M-H-C_7_H_4_O_4_]^-^;  108.0226[M-H-C_9_H_9_O_3_]^-^ |  |  |  | √ |  | √ |
| M40 | 8.66 | [M+H]^+^ | C_15_H_14_O_5_ | 274.0841 | 275.0909 | -1.81 | Liquiritin-Glu+O+2H | 275.1811[M+H]^+^;  165.0698[M+H-C_6_H_6_O_2_]^+^;  137.0230[M+H-C_8_H_10_O_2_]^+^;  135.0457[M+H-C_7_H_8_O_3_]^+^;  119.0500[M+H-C_7_H_8_O_3_]^+^ |  |  |  |  |  | √ |
| M41 | 8.68 | [M-H]^-^ | C_27_H_30_O_13_ | 562.1686 | 561.1615 | 0.15 | Liquiritin apioside-apiose-2O+GluA | 561.1563[M-H]^-^;  385.1294[M-H-GluA]^-^;  370.1094[M-H-C_6_H_10_O_4_-CHO_2_]^-^;  311.0569[M-H-C_6_H_10_O_3_-C_8_H_7_O]^-^;  297.0416[M-H-C_6_H_10_O_3_-C_8_H_7_O_2_]^-^;  253.0517[M-H-C_6_H_9_O_6_-C_6_H_11_O_3_]^-^ |  |  |  |  |  | √ |
| M42 | 8.71 | [M-H]^-^ | C_26_H_30_O_12_ | 534.1737 | 533.1669 | 0.79 | Liquiritin apioside-O | 533.1661[M-H]^-^;  443.1253[M-H-C_6_H_2_O]^-^;  427.1389[M-H-C_20_H_26_O_10_]^-^;  298.1217[M-H-C_5_H_8_O_3_-C_7_H_3_O_2_]^-^;  117.0197[M-C_21_H_20_O_9_]^-^ |  |  |  |  |  | √ |
| M43 | 8.75 | [M+H]^+^ | C_22_H_22_O_9_ | 430.1264 | 431.1342 | 1.32 | Liquiritin-Glu-O+CH_2_+GluA | 431.2934[M+H]^+^;  413.2707[M+H-H_2_O]^+^;  339.1117[M+H-C_6_H_4_O]^+^;  327.2449[M+H-C_7_H_4_O]^+^;  294.0721[M+H-C_6_H_4_O-CHO_2_]^+^;  121.0650[M+H-Glu A-C_9_H_10_O]^+^ |  |  |  |  |  | √ |
| M44 | 8.77 | [M+COOH]^-^ | C_22_H_24_O_9_ | 432.1419 | 477.1401 | -0.30 | Liquiritin+CH_2_ | 477.1401[M+COOH]^-^;  253.1251[M+COOH-HCOOH-C_6_H_10_O_6_]^-^;  135.0457[M+COOH-HCOOH-C_6_H_10_O_6_-C_8_H_8_O]^-^;  117.0186[M+COOH-HCOOH-C_6_H_10_O_6_-C_8_H_8_O-H_2_O]^-^ |  |  |  |  |  | √ |
| M45 | 8.84 | [M-H]^-^ | C_27_H_32_O_12_ | 548.1894 | 547.1828 | 1.22 | Liquiritin apioside-O+CH_2_ | 547.1837[M-H]^-^;  413.1575[M-H-C_7_H_3_O_3_]^-^;  249.1125[M-H-C_5_H_8_O_4_-C_7_H_3_O_3_-CH_3_O]^-^;  235.0980[M-H-C_5_H_9_O_5_-C_9_H_7_O_3_]^-^;  205.0863[M-H-C_5_H_9_O_5_-C_9_H_6_O_3_-CH_3_O]^-^;  135.0460[M-H-C_12_H_20_O_8_-C_8_H_8_O]^-^ |  |  |  |  |  | √ |
| M46 | 8.94 | [M-H]^-^ | C_22_H_24_O_10_ | 448.1369 | 447.1299 | 0.51 | Liquiritin apioside-apiose+O+CH_2_ | 447.1322[M-H]^-^;  429.1206[M-H-H_2_O]^-^;  313.1087[M-H-C_8_H_5_O_2_]^-^;  271.0984[M-H-C_10_H_8_O_3_]^-^;  135.0464[M-H-C_6_H_10_O_5_-C_8_H_6_O_3_]^-^ |  |  |  |  |  | √ |
| M47 | 9.05 | [M-H]^-^ | C_21_H_22_O_11_ | 450.1163 | 449.1090 | 0.20 | Isoliquiritin+2O | 449.1093[M-H]^-^;  271.0619[M-H-C_9_H_6_O_4_]^-^;  163.0413[M-H-C_15_H_10_O_6_]^-^;  135.0103[M-H-C_6_H_10_O_5_-C_7_H_3_O_4_]^-^;  109.0312[M-H-C_6_H_10_O_5_-C_9_H_6_O_3_-O]^-^ | √ |  |  |  |  | √ |
| M48 | 9.07 | [M-H]^-^ | C_42_H_62_O_19_S | 902.3590 | 901.3517 | -1.80 | Glycyrrhizic acid+Sul | 901.3356[M-H]^-^;  473.1461[M-H-C_6_H_8_O_10_S]^-^;  175.0253[M-H-C_21_H_40_O_17_S-CHO_2_]^-^ |  |  |  |  |  | √ |
| M49 | 9.14 | [M+H]^+^ | C_22_H_24_O_10_ | 448.1369 | 449.1437 | -1.21 | Liquiritin+O+CH_2_ | 449.3200[M+H]^+^;  146.1069[M+H-C_6_H_4_O_3_-C_6_H_11_O_6_]^+^;  137.0596[M+H-C_9_H_9_O-C_6_H_11_O_6_]^+^;  107.0492[M+H-C_10_H_11_O_2_-C_6_H_11_O_6_]^+^ |  |  |  |  |  | √ |
| M50 | 9.22 | [M-H]^-^ | C_21_H_22_O_10_ | 434.1213 | 433.1136 | -0.98 | Isoliquiritigenin+2H+GluA | 433.1153[M-H]^-^  ;257.0824[M-H-GluA]^-^;  193.0511[M-H-C_15_H_12_O_3_]^-^;  151.0409[M-H-GluA-C_7_H_6_O]^-^;  117.0206[M-H-GluA-C_7_H_8_O_3_]^-^ |  |  |  |  |  | √ |
| M51 | 9.23 | [M+H]^+^ | C_26_H_30_O_13_ | 550.1686 | 551.1756 | -0.49 | Liquiritin apioside+iso | 551.2974[M+H]^+^;  257.0808[M+H-C_5_H_10_O_4_-C_6_H_8_O_5_]^+^;  239.0688[M+H-C_5_H_10_O_4_-C_6_H_8_O_5_-H_2_O]^+^;  137.0230[M+H-C_5_H_10_O_4_-C_6_H_8_O_5_-C_8_H_8_O]^+^ |  |  |  |  |  | √ |
| M52 | 9.29 | [M-H]^-^ | C_21_H_22_O_8_ | 402.1315 | 401.1239 | -0.68 | Liquiritin apioside-apiose-O | 401.2002[M-H]^-^;  283.0609[M-H-C_7_H_2_O_2_]^-^;  255.0286[M-H-C_6_H_10_O_4_]^-^;  151.0401[M-H-C_6_H_10_O_4_-C_7_H_4_O]^-^;  121.0308[M-H-C_6_H_10_O_4_-C_8_H_6_O_2_]^-^ |  |  |  |  | √ |  |
| M53 | 9.36 | [M-H]^-^ | C_21_H_22_O_10_ | 434.1213 | 433.1144 | 0.93 | Liquiritin apioside-apiose+iso+O | 433.1120[M-H]^-^;  163.0400[M-H-C_15_H_10_O_5_]^-^;  151.0395[M-H-C_6_H_10_O_5_-C_7_H_4_O_2_]^-^;  135.0094[M-H-C_6_H_10_O_5_-C_7_H_4_O_3_]^-^ | √ |  |  |  |  | √ |
| M54 | 9.46 | [M+H]^+^ | C_23_H_24_O_10_ | 460.1369 | 461.1438 | -0.83 | Isoliquiritin+C_2_H_2_O | 461.2425[M+H]^+^;  256.0742[M+H-C_6_H_10_O_5_-C_2_H_2_O]^+^;  205.1049[M+H-C_12_H_16_O_6_]^+^;  137.0594[M+H-C_6_H_10_O_5_-C_2_H_2_O-C_8_H_7_O-]^+^ |  |  |  |  | √ |  |
| M55 | 9.55 | [M+H]^+^ | C_21_H_22_O_7_ | 386.1366 | 387.1438 | -0.16 | Liquiritin apioside-apiose-2O | 387.1476[M+H]^+^;  369.1349[M+H-H_2_O]^+^;  351.1195[M+H-2H_2_O]^+^;  313.0706[M+H-C_3_H_6_O_2_]^+^;  147.0437[M+H-C_6_H_12_O_3_-C_6_H_4_O_2_]^+^;  137.0591[M+H-C_6_H_12_O_3_-C_8_H_6_O]^+^ |  |  |  |  | √ |  |
| M56 | 9.58 | [M-H]^-^ | C_22_H_22_O_10_ | 446.1213 | 445.1143 | 0.59 | Liquiritin apioside-apiose-Glu+CH_2_+GluA | 445.1910[M-H]^-^;  269.0824[M-H-GluA]^-^;  254.0590[M-H-C_6_H_7_O_7_]^-^;  239.0357[M-H-C_9_H_5_O_3_-CHO_2_]^-^;  135.0105[M-H-C_6_H_7_O_7_-C_8_H_7_O]^-^ |  |  |  |  |  | √ |
| M57 | 9.61 | [M-H]^-^ | C_21_H_20_O_8_ | 400.1158 | 399.1083 | -0.71 | Liquiritin apioside-apiose-O-2H | 399.1474[M-H]^-^;  281.0457[M-H-C_7_H_2_O_2_]^-^;  253.0503[M-H-C_6_H_10_O_4_]^-^;  145.0301[M-H-C_6_H_10_O_4_-C_6_H_4_O_2_]^-^;  135.0088[M-H-C_6_H_10_O_4_-C_8_H_5_O]^-^;  119.0504[M-H-C_6_H_10_O_4_-C_8_H_5_O-O]^-^ |  |  |  |  | √ |  |
| M58 | 9.68 | [M+H]^+^ | C_15_H_14_O_4_ | 258.0885 | 259.0957 | -2.90 | Liquiritin-Glu+2H | 259.0957[M+H]^+^;  148.0475[M+H-C_6_H_8_O_2_]^+^;  138.0266[M+H-C_8_H_9_O]^+^;  119.0488[M+H-C_6_H_8_O_2_-CHO]^+^;  107.0500[M+H-C_8_H_8_O_3_]^+^ |  |  |  |  |  | √ |
| M59 | 9.69 | [M+H]^+^ | C_15_H_10_O_4_ | 254.0579 | 255.0649 | -1.18 | Liquiritigenin-2H | 255.0649[M+H]^+^;  237.0533[M+H-H_2_O]^+^;  199.0741[M+H-C_3_H_4_O]^+^;  145.0286[M+H-C_6_H_4_O_2_]^+^;  137.0236[M+H-C_8_H_6_O]^+^;  121.0293[M+H-C_8_H_6_O_2_]^+^;  117.0337[M+H-C_7_H_6_O_3_]^+^;  109.0288[M+H-C_9_H_6_O_2_]^+^ |  |  |  |  | √ |  |
| M60 | 9.76 | [M+H]^+^ | C_21_H_22_O_10_ | 434.1193 | 435.1266 | -4.60 | Isoliquiritin+O | 435.1266[M+H]^+^;  257.0743[M+H-C_6_H_10_O_5_-O]^+^;  153.0545[M+H-C_6_H_12_O_5_-C_8_H_6_O]^+^;  149.0593[M+H-C_6_H_12_O_5_-C_7_H_5_O]^+^;  135.0446[M+H-C_6_H_10_O_5_-O-C_8_H_10_O]^+^;  107.0499[M+H-C_6_H_12_O_5_-C_6_H_5_O_3_-H_2_O]^+^ | √ |  |  |  |  |  |
| M61 | 9.79 | [M+H]^+^ | C_17_H_14_O_4_ | 282.0890 | 283.0963 | -0.66 | Liquiritin-Glu+C_2_H_2_O-O | 283.0963[M+H]^+^;  163.0399[M+H-C_8_H_8_O]^+^;  145.0307[M+H-C_6_H_7_-C_2_H_3_O_2_]^+^;  135.0470[M+H-C_9_H_8_O_2_]^+^;  131.0833[M+H-C_8_H_8_O_3_]^+^;  119.0502[M+H-C_9_H_8_O_2_-O]^+^;  107.0503[M+H-C_9_H_9_O-C_2_H_3_O]^+^ |  |  |  |  |  | √ |
| M62 | 9.81 | [M-H]^-^ | C_21_H_20_O_10_ | 432.1056 | 431.0982 | -0.51 | Isoliquiritigenin+GluA | 431.2153[M-H]^-^;  255.0664[M-H-GluA]^-^;  149.0245[M-H-GluA-C_7_H_6_O]^-^;  135.0091[M-H-GluA-C_8_H_8_O]^-^;  119.0510[M-H-GluA-C_7_H_4_O_3_]^-^ | √ |  |  |  |  | √ |
| M63 | 9.97 | [M+H]^+^ | C_21_H_20_O_7_ | 384.1209 | 385.1281 | -0.12 | Isoliquiritin-O-H_2_O | 385.2464[M+H]^+^;  352.0948[M+H-CH_5_O]^+^;  209.1154[M+H-C_6_H_14_O_3_-C_2_H_2_O]^+^;  137.0236[M+H-C_6_H_12_O_3_-C_8_H_5_O]^+^ |  |  |  |  |  | √ |
| M64 | 10.07 | [M-H]^-^ | C_27_H_32_O_12_ | 548.1892 | 547.1820 | -0.30 | Liquiritin apioside-O+CH_2_+iso | 547.1827[M-H]^-^;  413.1581[M-H-C_5_H_10_O_4_]^-^;  255.0669[M-H-C_12_H_20_O_8_]^-^;  253.0901[M-H-C_5_H_8_O_4_-C_9_H_6_O_3_]^-^;  135.0083[M-H-C_12_H_20_O_8_-C_8_H_8_O]^-^ |  |  |  |  |  | √ |
| M65 | 10.10 | [M+H]^+^ | C_15_H_10_O_5_ | 270.0528 | 271.0596 | -1.84 | Liquiritin apioside-apiose-Glu+O-2H | 271.0609[M+H]^+^;  229.0501[M+H-C_2_H_2_O]^+^;  179.0353[M+H-C_6_H_4_O]^+^;  137.0227[M+H-C_8_H_8_O]^+^;  121.02998[M+H-C_7_H_4_O_3_]^+^ |  |  |  |  |  | √ |
| M66 | 10.16 | [M+H]^+^ | C_16_H_14_O_5_ | 286.0841 | 287.0909 | -1.63 | Liquiritigenin+O+CH_2_ | 287.0866[M+H]^+^;  181.0655[M+H-C_7_H_6_O]^+^;  177.0549[M+H-C_6_H_6_O_2_]^+^;  163.0396[M+H-C_6_H_6_O_2_-CH_2_]^+^;  149.0601[M+H-C_6_H_6_O_2_-C_2_H_4_]^+^;  137.0241[M+H-C_8_H_6_O_3_]^+^;  134.0371[M+H-C_6_H_6_O_2_-C_2_H_3_O]^+^;  123.0446[M+H-C_6_H_6_O_2_-C_3_H_2_O]^+^;  109.0298[M+H-C_10_H_10_O_3_]^+^ |  |  |  |  |  | √ |
| M67 | 10.21 | [M-H]^-^ | C_42_H_64_O_17_ | 840.4144 | 839.4079 | 0.99 | Glycyrrhizic acid+O+2H | 839.4124[M-H]^-^;  663.3782[M-H-GluA]^-^;  351.0574[M-H-C_30_H_48_O_5_]^-^;  193.0353[M-H-C_30_H_48_O_5_-C_6_H_8_O_5_]^-^;  113.0254[M-H-C_30_H_48_O_5_-C_6_H_8_O_5_-CO_2_-2H_2_O]^-^ |  |  |  |  | √ |  |
| M68 | 10.24 | [M+H]^+^ | C_21_H_22_O_6_ | 370.1416 | 371.1492 | 0.65 | Liquiritin apioside-apiose-3O | 371.1113[M+H]^+^;  311.1006[M+H-C_3_H_8_O]^+^;  267.0630[M+H-C_5_H_12_O_2_]^+^;  131.0861[M+H-C_15_H_12_O_3_]^+^;  107.0502[M+H-C_6_H_12_O_3_-C_9_H_8_O]^+^ |  |  |  |  | √ |  |
| M69 | 10.26 | [M-H]^-^ | C_22_H_24_O_10_ | 448.1369 | 447.1299 | 0.59 | Isoliquiritin+O+CH_2_ | 447.1318[M-H]^-^;  271.0983[M-H-C_10_H_8_O_3_]^-^;  241.0509[M-H-C_10_H_7_O_3_-CH_3_O]^-^;  149.0621[M-H-C_16_H_11_O_4_-CH_3_O]^-^;  135.0468[M-H-C_6_H_10_O_5_-C_8_H_6_O_3_]^-^ | √ |  |  |  |  | √ |
| M70 | 10.35 | [M-H]^-^ | C_42_H_62_O_18_ | 854.3936 | 853.3865 | 0.21 | Glycyrrhizic acid+2O | 853.3881[M-H]^-^;  441.1784[M-H-C_12_H_16_O_13_-CO_2_]^-^;  425.1822[M-H-C_12_H_17_O_13_-CO_2_-CH_3_]^-^;  411.2000[M-H-C_15_H_21_O_3_-C_6_H_9_O_7_]^-^;  249.1495[M-H-C_27_H_39_O_15_]^-^;  175.0255[M-H-C_15_H_21_O_3_-C_21_H_31_O_8_-H_2_O]^-^;  113.0267[M-H-C_15_H_21_O_3_-C_21_H_31_O_8_-H_2_O-HO]^-^ |  |  |  |  |  | √ |
| M71 | 10.39 | [M-H]^-^ | C_21_H_20_O_9_ | 416.1107 | 415.1033 | -0.46 | Liquiritin apioside-apiose-Glu+GluA-O+iso | 415.2182[M-H]^-^;  255.0696[M-H-C_6_H_8_O_5_]^-^;  163.0363[M-H-C_6_H_8_O_5_-C_6_H_4_O]^-^;  151.0401[M-H-C_6_H_8_O_5_-C_7_H_5_O]^-^;  109.0325[M-H-C_6_H_8_O_5_-C_9_H_6_O_2_]^-^ | √ |  |  |  |  |  |
| M72 | 10.46 | [M-H]^-^ | C_15_H_10_O_5_ | 270.0528 | 269.0451 | -1.82 | Liquiritin apioside-apiose-Glu+O-2H | 269.0434[M-H]^-^;  252.0388[M-H-H_2_O]^-^;  133.0274[M-H-C_7_H_4_O_3_]^-^;  121.0321[M-H-C_8_H_4_O_3_]^-^ |  |  |  |  | √ |  |
| M73 | 10.55 | [M-H]^-^ | C_15_H_12_O_7_S | 336.0304 | 335.0230 | -0.18 | Isoliquiritigenin+Sul | 335.0242[M-H]^-^;  255.0673[M-H-SO_3_]^-^;  149.0247[M-H-SO_3_-C_7_H_6_O]^-^;  135.0101[M-H-SO_3_-C_8_H_8_O]^-^;  119.0521[M-H-SO_3_-C_7_H_4_O_3_]^-^ |  |  |  |  | √ | √ |
| M74 | 10.65 | [M+H]^+^ | C_21_H_24_O_7_ | 388.1522 | 389.1586 | -2.17 | Liquiritin-2O+2H+iso | 389.2797[M+H]^+^;  297.0737[M+H-C_7_H_8_]^+^;  285.2336[M+H-C_8_H_8_]^+^;  135.0428[M+H-C_7_H_7_-C_6_H_11_O_5_]^+^;  121.0663[M+H-C_8_H_9_-C_6_H_11_O_5_]^+^ |  |  |  |  |  | √ |
| M75 | 10.69 | [M+H]^+^ | C_22_H_24_O_10_ | 448.1365 | 449.1438 | -1.00 | Liquiritin apioside-apiose+O+CH_2_+iso | 449.3294[M+H]^+^;  295.0835[M+H-C_7_H_6_O_4_]^+^;  285.1133[M+H-C_6_H_12_O_5_]^+^;  163.0753[M+H-C_16_H_14_O_5_]^+^;  161.0601[M+H-C_6_H_12_O_5_-C_6_H_4_O_3_]^+^ | √ |  |  |  |  | √ |
| M76 | 10.91 | [M-H]^-^ | C_22_H_24_O_10_S | 480.1089 | 479.1016 | -0.20 | Isoliquiritin-2O+CH_2_+Sul+iso | 479.1009M-H]^-^;  365.2331[M-H-SO_4_-H_2_O]^-^;  339.0863[M-H-C_6_H_5_O_2_-CH_3_O]^-^;  252.0516[M-H-C_6_H_10_O_2_S]^-^ |  |  |  |  |  | √ |
| M77 | 10.99 | [M-H]^-^ | C_15_H_14_O_5_ | 274.0841 | 273.0767 | -0.52 | Isoliquiritigenin+H_2_O | 273.0822[M-H]^-^;  167.0376[M-H-C_7_H_6_O]^-^;  151.0425[M-H-C_7_H_6_O-O]^-^;  135.0094[M-H-C_7_H_7_O-CH_3_]^-^;  109.0312[M-H-C_7_H_6_O-C_2_H_2_O_2_]^-^;  107.0522[M-H-C_8_H_6_O_4_]^-^ |  |  |  |  | √ |  |
| M78 | 11.10 | [M+H]^+^ | C_21_H_20_O_8_ | 400.1158 | 401.1230 | -0.14 | Liquiritin apioside-apiose-2H-O | 401.1263[M+H]^+^;  307.0568[M+H-C_6_H_6_O]^+^;  279.1134[M+H-C_7_H_6_O_2_]^+^;  235.1517[M+H-C_6_H_12_O_4_-H_2_O]^+^ |  |  |  |  | √ |  |
| M79 | 11.24 | [M-H]^-^ | C_21_H_24_O_9_ | 420.1420 | 419.1341 | -1.46 | Liquiritin+2H | 419.1366[M-H]^-^;  239.0569[M-H-C_6_H_10_O_5_-H_2_O]^-^;  179.0348[M-H-C_15_H_12_O_3_]^-^;  137.0608[M-H-C_9_H_8_O_3_-C_8_H_6_O]^-^;  119.0512[M-H-C_9_H_8_O_3_-C_7_H_4_O_3_]^-^ |  |  |  |  | √ |  |
| M80 | 11.37 | [M-H]^-^ | C_22_H_22_O_10_ | 446.1213 | 445.1145 | 0.97 | Liquiritin apioside-apiose-Glu+CH_2_+GluA+iso | 445.2114[M-H]^-^;  269.0817[M-H-GluA]^-^;  254.0574[M-H-GluA-CH_3_]^-^;  117.0196[M-H-GluA-C_8_H_8_O_3_]^-^ | √ |  |  |  |  |  |
| M81 | 11.48 | [M+H]^+^ | C_22_H_22_O_10_ | 446.1213 | 447.1284 | -0.40 | Isoliquiritigenin+CH_2_+GluA | 447.2565[M+H]^+^;  411.2624[M+H-2H_2_O]^+^;  271.0958[M+H-GluA]^+^;  161.0612[M+H-C_6_H_10_O_5_-O-C_6_H_4_O_2_]^+^;  137.0598[M+H-C_6_H_10_O_5_-O-C_9_H_8_O]^+^;  105.0709[M+H-C_6_H_10_O_5_-O-C_9_H_8_O_3_]^+^ |  |  |  |  |  | √ |
| M82 | 11.54 | [M+H]^+^ | C_15_H_12_O_3_ | 240.0786 | 241.0852 | -3.11 | Liquiritin apioside-apiose-Glu-O | 241.1005[M+H]^+^;  197.0593[M+H-C_2_H_4_O]^+^;  147.0445[M+H-C_6_H_6_O]^+^;  131.0499[M+H-C_6_H_6_O_2_]^+^;  119.0503[M+H-C_7_H_6_O_2_]^+^;  107.0502[M+H-C_9_H_10_O]^+^ |  |  |  |  | √ | √ |
| M83 | 11.70 | [M-H]^-^ | C_42_H_64_O_16_ | 824.4194 | 823.4124 | 0.26 | Glycyrrhizic acid+2H | 823.4149[M-H]^-^;  761.4151[M-H-CO_2_-H_2_O]^-^;  647.3833[M-H-GluA]^-^;  351.0565[M-H-C_30_H_48_O_4_]^-^;  193.0352[M-H-C_30_H_48_O_4_-C_6_H_8_O_5_]^-^;  175.0252[M-H-C_30_H_48_O_4_-C_6_H_8_O_5_-H_2_O]^-^;  113.0253[M-H-C_30_H_48_O_4_-C_6_H_8_O_5_-2H_2_O-CO_2_]^-^ |  |  |  |  | √ |  |
| M84 | 12.14 | [M+H]^+^ | C_15_H_12_O_5_ | 272.0685 | 273.0751 | -2.27 | Isoliquiritigenin+O | 273.0752[M+H]^+^;  153.0184[M+H-C_8_H_8_O]^+^;  147.0445[M+H-C_6_H_6_O_3_]^+^;  119.0502[M+H-C_7_H_6_O_4_]^+^;  107.0499[M+H-C_8_H_6_O_4_]^+^ |  |  |  |  |  | √ |
| M85 | 12.17 | [M-H]^-^ | C_28_H_32_O_12_ | 560.1896 | 559.1823 | 0.40 | Liquiritin apioside-2O+C_2_H_2_O | 559.1818[M-H]^-^;  427.1813[M-H-C_5_H_8_O_4_]^-^;  383.1514[M-H-C_2_H_2_O_2_-C_7_H_3_O_2_]^-^;  368.0848[M-H-C_5_H_8_O_4_-C_2_H_3_O_2_]^-^;  297.0389[M-H-C_5_H_8_O_4_-C_6_H_10_O_3_]^-^;  178.0270[M-H-C_5_H_8_O_4_-C_6_H_10_O_3_-C_8_H_7_O]^-^ |  |  |  |  |  | √ |
| M86 | 12.25 | [M-H]^-^ | C_30_H_46_O_8_S | 566.2913 | 565.2832 | -1.51 | Glycyrrhetinic acid+O+Sul | 565.2851[M-H]^-^;  535.2377[M-H-O-CH_2_]^-^;  455.2805[M-H-SO_4_-CH_2_]^-^ |  |  |  |  | √ |  |
| M87 | 12.28 | [M+H]^+^ | C_21_H_20_O_7_ | 384.1209 | 385.1282 | -0.02 | Liquiritin apioside-apiose-2O-2H | 385.1293[M+H]^+^;  313.0714[M+H-C_4_H_8_O]^+^;  149.0240[M+H-C_6_H_12_O_3_-C_7_H_5_O]^+^;  135.0439[M+H-C_6_H_12_O_3_-C_8_H_4_O]^+^;  107.0488[M+H-C_6_H_12_O_3_-C_9_H_6_O_2_]^+^ |  |  |  |  | √ |  |
| M88 | 12.31 | [M+H]^+^ | C_42_H_62_O_18_ | 854.3936 | 855.4014 | 0.54 | Glycyrrhizic acid+2O | 855.3933[M+H]^+^;  661.3578[M+H-C_6_H_10_O_7_]^+^;  485.3258[M+H-C_6_H_10_O_7_-GluA]^+^;  261.1491[M+H-C_6_H_10_O_7_-GluA-C_14_H_24_O_2_]^+^;  233.1543[M+H-C_6_H_10_O_7_-GluA-C_15_H_29_O_3_]^+^ |  |  |  |  | √ |  |
| M89 | 12.31 | [M+H]^+^ | C_16_H_16_O_4_ | 272.1049 | 273.1114 | -2.56 | Liquiritin apioside-apiose-Glu+2H+CH_2_ | 273.0756[M+H]^+^;  137.0596[M+H-C_9_H_12_O]^+^;  122.0357[M+H-C_9_H_11_O_2_]^+^;  109.0653[M+H-C_10_H_12_O_2_]^+^;  107.0502[M+H-C_9_H_10_O_3_]^+^ |  |  |  |  | √ | √ |
| M90 | 12.35 | [M+COOH]^-^ | C_15_H_10_O_3_ | 238.0636 | 283.0618 | 2.20 | Isoliquiritin-Glu-O-2H+iso | 283.0625[M+COOH]^-^;  195.0461[M+COOH-HCOOH-C_2_H_2_O]^-^;  143.0508[M+COOH-HCOOH-C_6_H_4_-H_2_O]^-^;  135.0098[M+COOH-HCOOH-C_8_H_6_]^-^;  117.0351[M+COOH-HCOOH-C_7_H_4_O_2_]^-^ |  |  |  |  |  | √ |
| M91 | 12.51 | [M+COOH]^-^ | C_15_H_10_O_3_ | 238.0630 | 283.0607 | 2.27 | Isoliquiritin-Glu-H_2_O+iso | 283.0635[M+COOH]^-^;  211.0406[M+COOH-HCOOH-C_2_H_2_]^-^;  195.0458[M+COOH-HCOOH-C_2_H_2_O]^-^;  183.0451[M+COOH-HCOOH-C_3_H_2_O]^-^ |  |  |  |  | √ |  |
| M92 | 12.62 | [M+H]^+^ | C_16_H_14_O_4_ | 270.0892 | 271.0959 | -2.23 | Liquiritigenin+CH_2_ | 271.0590[M+H]^+^;  239.0707[M+H-CH_4_O]^+^;  177.0534[M+H-C_6_H_6_O]^+^;  162.0304[M+H-C_6_H_5_O_2_]^+^;  134.0361[M+H-C_6_H_5_O_2_-CO]^+^;  121.0287[M+H-C_9_H_10_O_2_]^+^;  107.0495[M+H-C_9_H_8_O_3_]^+^ |  |  |  |  | √ | √ |
| M93 | 12.65 | [M-H]^-^ | C_26_H_28_O_11_ | 516.1632 | 515.1562 | 0.61 | Liquiritin apioside-2O-2H | 515.2871[M-H]^-^;  339.1239[M-H-C_7_H_12_O_5_]^-^;  175.0739[M-H-C_19_H_15_O_6_]^-^;  133.0662[M-H-C_5_H_8_O_4_-C_6_H_10_O_3_-C_8_H_8_O]^-^;  119.0504[M-H-C_5_H_8_O_4_-C_6_H_10_O_3_-C_8_H_6_O_2_]^-^ | √ |  |  |  |  |  |
| M94 | 12.83 | [M+H]^+^ | C_15_H_10_O_5_ | 270.0528 | 271.0598 | -1.17 | Isoliquiritin-Glu+O-2H | 271.0603[M+H]^+^;  253.0503[M+H-H_2_O]^+^;  229.0496[M+H-C_2_H_2_O]^+^;  163.0385[M+H-C_6_H_4_O_2_]^+^;  153.0182[M+H-C_8_H_6_O]^+^;  145.0289[M+H-C_6_H_6_O_3_]^+^;  119.0500[M+H-H_2_O-C_8_H_6_O_2_]^+^ |  |  |  |  |  | √ |
| M95 | 12.97 | [M-H]^-^ | C_22_H_24_O_10_S | 480.1090 | 479.1013 | -0.95 | Liquiritin-2O+CH_2_+Sul+iso | 479.1017[M-H]^-^;  399.1440[M-H-SO_3_]^-^;  367.1197[M-H-HSO_3_-CH_3_O]^-^;  263.0914[M-H-SO_3_-C_7_H_4_O_3_]^-^;  231.0991[M-H-SO_3_-C_7_H_4_O_3_-CH_4_O]^-^;  135.0453[M-H-C_15_H_20_O_7_S]^-^ |  |  |  |  | √ |  |
| M96 | 12.97 | [M-H]^-^ | C_27_H_32_O_11_ | 532.1945 | 531.1872 | 0.02 | Liquiritin apioside-2O+CH_2_ | 531.1890[M-H]^-^;  411.1892[M-H-C_7_H_4_O_2_]^-^;  397.1678[M-H-C_8_H_4_O_2_]^-^;  355.1560[M-H-C_10_H_8_O_3_]^-^;  254.0588[M-H-C_11_H_17_O_8_]^-^;  135.0464[M-H-C_11_H_18_O_7_-C_8_H_6_O_2_]^-^ |  |  |  |  |  | √ |
| M97 | 13.13 | [M-H]^-^ | C_27_H_32_O_11_ | 532.1945 | 531.1878 | 1.25 | Liquiritin apioside-2O+CH_2_+iso | 531.1909[M-H]^-^;  383.3025[M-H-C_5_H_8_O_5_]^-^;  355.1556[M-H-C_10_H_8_O_3_]^-^;  233.1171[M-H-C_5_H_8_O_5_-C_8_H_6_O_3_]^-^;  219.0964[M-H-C_5_H_8_O_5_-C_9_H_8_O_3_]^-^;  205.0851[M-H-C_5_H_8_O_5_-C_10_H_10_O_3_]^-^;  147.0456[M-H-C_5_H_8_O_4_-C_6_H_10_O_3_-C_7_H_6_O_2_]^-^;  135.0439[M-H-C_5_H_8_O_4_-C_6_H_10_O_3_-C_8_H_7_O-CH_3_]^-^ | √ |  |  |  |  |  |
| M98 | 13.17 | [M-H]^-^ | C_26_H_28_O_13_ | 548.1528 | 547.1455 | -0.40 | Liquiritin apioside-2H | 547.1511[M-H]^-^;  427.1948[M-H-C_7_H_4_O_2_]^-^;  411.2005[M-H-C_7_H_3_O_3_]^-^;  324.0991[M-H-C_6_H_3_O-C_5_H_8_O_4_]^-^ |  |  |  |  |  | √ |
| M99 | 13.34 | [M-H]^-^ | C_21_H_24_O_7_ | 388.1522 | 387.1444 | -1.23 | Liquiritin apioside-apiose-2O+2H | 387.2244[M-H]^-^;  219.1042[M-H-C_7_H_5_O_3_-CH_3_O]^-^;  207.1023[M-H-C_6_H_4_O_2_-C_4_H_8_O]^-^;  205.0876[M-H-C_6_H_4_O-C_3_H_6_O_2_]^-^;  192.0809[M-H-C_7_H_4_O_3_-C_3_H_6_O]^-^;  151.0398[M-H-C_13_H_16_O_4_]^-^;  149.0262[M-H-C_8_H_6_O_3_-C_4_H_8_O_2_]^-^;  137.0250[M-H-C_14_H_18_O_4_]^-^;  108.0243[M-H-C_15_H_19_O_5_]^-^ |  |  |  |  | √ |  |
| M100 | 13.65 | [M-H]^-^ | C_15_H_14_O_4_ | 258.0892 | 257.0818 | -0.66 | Isoliquiritigenin+2H | 257.0805[M-H]^-^;  151.0402[M-H-C_7_H_6_O]^-^;  122.0384[M-H-C_7_H_3_O_3_]^-^;  109.0305[M-H-C_9_H_8_O_2_]^-^;  107.0513[M-H-C_8_H_6_O_3_]^-^ | √ | √ | √ | √ | √ | √ |
| M101 | 13.83 | [M+H]^+^ | C_22_H_24_O_7_ | 400.1522 | 401.1608 | 3.32 | Liquiritin apioside-apiose-2O+CH_2_+iso | 401.1750[M+H]^+^;  279.0338[M+H-C_7_H_6_O_2_]^+^;  221.1262[M+H-C_6_H_12_O_4_-CH_4_O]^+^;  179.0679[M+H-C_6_H_11_O_4_-C_6_H_4_]^+^  ;123.0449[M+H-C_6_H_12_O_4_-C_9_H_6_O]^+^ |  |  |  |  | √ |  |
| M102 | 13.83 | [M-H]^-^ | C_42_H_60_O_17_ | 836.3831 | 835.3766 | 0.98 | Glycyrrhizic acid+O-2H | 835.3797[M-H]^-^;  659.3478[M-H-GluA]^-^;  351.0569[M-H-C_30_H_44_O_5_]^-^;  193.0353[M-H-C_30_H_44_O_5_-C_6_H_8_O_5_]^-^;  113.0252[M-H-C_30_H_44_O_5_-C_6_H_8_O_5_-2H_2_O-CO_2_]^-^ |  |  |  |  | √ |  |
| M103 | 13.90 | [M-H]^-^ | C_26_H_30_O_12_ | 534.1737 | 533.1659 | -0.99 | Liquiritin apioside-O+iso | 533.1687[M-H]^-^;  487.2383[M-H-HCOOH]^-^;  423.1328[M-H-C_6_H_6_O_2_]^-^;  117.0200[M-H-C_21_H_20_O_9_]^-^;  109.0309[M-H-C_20_H_24_O_10_]^-^ |  |  |  |  |  | √ |
| M104 | 13.97 | [M+H]^+^ | C_42_H_60_O_17_ | 836.3831 | 837.3910 | 0.75 | Glycyrrhizic acid+O-2H | 837.3897[M+H]^+^;  643.3473[M+H-C_6_H_10_O_7_]^+^;  625.3368[M+H-C_6_H_10_O_7_-H_2_O]^+^;  467.3131[M+H-C_6_H_10_O_7_-GluA]^+^;  449.3038[M+H-C_6_H_10_O_7_-H_2_O]^+^;  355.2255[M+H-C_6_H_10_O_7_-GluA-C_6_H_7_O_2_]^+^;  233.1530[M+H-C_6_H_10_O_7_-GluA-C_15_H_23_O_2_]^+^ |  |  |  |  | √ |  |
| M105 | 14.07 | [M+H]^+^ | C_21_H_20_O_6_ | 368.1260 | 369.1332 | -0.13 | Isoliquiritin-2O-H_2_O | 369.1317[M+H]^+^;  229.0909[M+H-C_6_H_5_O_2_-CH_3_O]^+^;  137.0601[M+H-C_6_H_12_O_2_-C_8_H_4_O]^+^;  131.0856[M+H-C_15_H_10_O_3_]^+^ |  |  |  |  | √ |  |
| M106 | 14.1 | [M+COOH]^-^ | C_15_H_14_O_3_ | 242.0949 | 287.0931 | 0.07 | Isoliquiritin-Glu-O+2H | 287.0929[M+COOH]^-^;  242.1760[M+COOH-HCOOH]^-^;  166.0272[M+COOH-HCOOH-C_6_H_3_]^-^;  151.0400[M+COOH-HCOOH-C_7_H_6_]^-^;  136.0181[M+COOH-HCOOH-C_8_H_9_]^-^;  109.0290[M+COOH-HCOOH-C_9_H_8_O]^-^ |  |  |  | √ |  |  |
| M107 | 14.11 | [M+H]^+^ | C_30_H_46_O_6_ | 502.3294 | 503.3367 | -0.11 | Glycyrrhetinic acid+2O | 503.3357[M+H]^+^;  485.3266[M+H-H_2_O]^+^;  467.3163[M+H-2H_2_O]^+^;  439.3207[M+H-2H_2_O-CO]^+^;  333.2066[M+H-C_10_H_18_O_2_]^+^;  315.1961[M+H-C_10_H_18_O_2_-H_2_O]^+^;  233.1538[M+H-C_15_H_26_O_4_]^+^ |  |  |  |  | √ |  |
| M108 | 14.29 | [M+H]^+^ | C_21_H_22_O_6_ | 370.1415 | 371.1488 | -0.30 | Liquiritin apioside-apiose-3O+iso | 371.2560[M+H]^+^;  297.0433[M+H-C_4_H_10_O]^+^;  283.0576[M+H-C_4_H_8_O_2_]^+^;  261.1089[M+H-C_6_H_6_O_2_]^+^;  237.1641[M+H-C_6_H_12_O_2_-H_2_O]^+^;  233.1187[M+H-C_7_H_6_O_3_]^+^;  145.1007[M+H-C_6_H_12_O_2_-C_6_H_6_O_2_]^+^;  115.0542[M+H-C_15_H_12_O_4_]^+^ |  |  |  |  | √ |  |
| M109 | 14.30 | [M-H]^-^ | C_22_H_24_O_11_S | 496.1028 | 495.0956 | -2.20 | Isoliquiritin-O+CH_2_+Sul | 495.0972[M-H]^-^;  415.1405[M-H-SO_3_]^-^;  195.1026[M-H-C_16_H_13_O_4_-CH_3_O]^-^;  163.0762[M-H-C_6_H_11_O_7_S-C_7_H_5_O]^-^;  151.0406[M-H-SO_3_-C_6_H_10_O_4_-C_8_H_6_O]^-^ |  |  |  |  | √ |  |
| M110 | 14.70 | [M+H]^+^ | C_16_H_14_O_4_ | 270.0892 | 271.0955 | -3.57 | Isoliquiritigenin+CH_2_ | 271.0878[M+H]^+^;  255.0542[M+H-CH_4_]^+^;  239.0610[M+H-CH_3_-HO]^+^;  165.0686[M+H-C_7_H_6_O]^+^;  107.0498[M+H-C_9_H_8_O_3_]^+^ |  |  |  |  | √ | √ |
| M111 | 14.90 | [M-H]^-^ | C_42_H_64_O_16_ | 824.4194 | 823.4125 | 0.39 | Glycyrrhizic acid+2H | 823.4154[M-H]^-^;  777.4101[M-H-HCOOH]^-^;  351.0572[M-H-C_30_H_48_O_3_]^-^;  193.0361[M-H-C_30_H_48_O_3_-C_6_H_8_O_5_]^-^;  113.0256[M-H-C_30_H_48_O_3_-C_6_H_8_O_5_-2H_2_O-CO_2_]^-^ |  |  |  |  | √ |  |
| M112 | 15.18 | [M+H]^+^ | C_15_H_12_O_3_ | 240.0786 | 241.0851 | -3.39 | Isoliquiritigenin-O | 241.0843[M+H]^+^;  137.0243[M+H-C_6_H_6_-CO]^+^;  131.0506[M+H-C_6_H_6_O_2_]^+^;  103.0556[M+H-C_6_H_6_O_2_-CO]^+^ |  |  |  |  |  | √ |
| M113 | 15.29 | [M+H]^+^ | C_32_H_48_O_4_ | 496.3553 | 497.3624 | -0.32 | Glycyrrhizic acid-2GluA-O+C_2_H_2_O | 497.2805[M+H]^+^;  437.3391[M+H-C_2_H_4_O_2_]^+^;  381.1352[M+H-C_2_H_4_O_2_-C_4_H_8_]^+^;  285.2200[M+H-C_2_H_4_O_2_-C_11_H_20_]^+^;  219.1733[M+H-C_2_H_4_O-C_15_H_22_O]^+^ |  |  |  |  | √ |  |
| M114 | 15.30 | [M+H]^+^ | C_17_H_14_O_5_ | 298.0841 | 299.0911 | -1.09 | Liquiritin apioside-apiose-Glu+C_2_H_2_O+iso | 299.0911[M+H]^+^;  284.0673[M+H-CH_3_]^+^;  256.0727[M+H-C_2_H_3_O]^+^;  239.0704[M+H-C_2_H_4_O_2_]^+^;  213.0547[M+H-C_2_H_4_O_2_-CO]^+^ |  |  |  |  | √ | √ |
| M115 | 15.53 | [M-H]^-^ | C_21_H_22_O_8_ | 402.1315 | 401.1239 | -0.76 | Liquiritin apioside-apiose+iso-O | 401.2144[M-H]^-^;  261.0419[M-H-C_6_H_5_O_2_-CH_3_O]^-^;  189.0565[M-H-C_6_H_4_O_2_-C_4_H_8_O_3_]^-^;  176.0128[M-H-C_7_H_4_O_3_-C_4_H_9_O_2_]^-^109.0315[M-H-C_15_H_16_O_6_]^-^ |  |  |  |  | √ |  |
| M116 | 15.60 | [M-H]^-^ | C_42_H_60_O_16_ | 820.3881 | 819.3817 | 1.07 | Glycyrrhizic acid-2H | 819.3866[M-H]^-^;  757.3863[M-H-CO_2_-H_2_O]^-^;  643.3536[M-H-GluA]^-^;  351.0578[M-H-C_30_H_42_O_4_]^-^;  193.0360[M-H-C_30_H_42_O_4_-C_6_H_8_O_5_]^-^;  113.0261[M-H-C_30_H_48_O_4_-C_6_H_8_O_5_-2H_2_O-CO_2_]^-^ |  |  |  |  | √ |  |
| M117 | 15.65 | [M+H]^+^ | C_16_H_14_O_5_ | 286.0841 | 287.0909 | -1.64 | Isoliquiritigenin+O+CH_2_ | 287.0917[M+H]^+^;  271.0598[M+H-CH_4_]^+^;  177.0548[M+H-C_6_H_6_O_2_]^+^;  163.0405[M+H-C_7_H_8_O_2_]^+^;  137.0242[M+H-C_8_H_8_O_2_-CH_2_]^+^;  134.0371[M+H-C_7_H_9_O_2_-CO]^+^ |  |  |  |  |  | √ |
| M118 | 15.67 | [M-H]^-^ | C_42_H_62_O_17_ | 838.3987 | 837.3923 | 1.05 | Glycyrrhizic acid+O | 837.3966[M-H]^-^;  775.3967[M-H-CO_2_-H_2_O]^-^;  661.3630[M-H-GluA]^-^;  351.0577[M-H-C_30_H_46_O_5_]^-^;  193.0363[M-H-C_30_H_46_O_5_-C_6_H_8_O_5_]^-^;  113.0263[M-H-C_30_H_47_O_4_-C_6_H_8_O_5_-2H_2_O-CO_2_]^-^ | √ |  |  |  | √ | √ |
| M119 | 15.85 | [M+H]^+^ | C_36_H_54_O_11_ | 662.3666 | 663.3742 | 0.51 | Glycyrrhetinic acid+O+GluA | 663.3735[M+H]^+^;  645.3678[M+H-H_2_O]^+^;  487.3421[M+H-GluA]^+^;  469.3332[M+H-GluA-H_2_O]^+^;317.2136[M+H-GluA-H_2_O-C_10_H_16_O]^+^;  189.1633[M+H-GluA-H_2_O-C_15_H_22_O_2_-H_2_O-CO]^+^ |  |  |  |  | √ |  |
| M120 | 15.86 | [M+H]^+^ | C_42_H_62_O_17_ | 838.3987 | 839.4065 | 0.60 | Glycyrrhizic acid+O | 839.4088[M+H]^+^;  663.3745[M+H-GluA]^+^;  645.3622[M+H-GluA-H_2_O]^+^;  627.3526[M+H-GluA-2H_2_O]^+^;  469.3294[M+H-2GluA-H_2_O]^+^;  439.3205[M+H-C_12_H_18_O_12_-H_2_O-CO]^+^;  317.2125[M+H-2GluA-H_2_O-C_10_H_17_O]^+^;  263.1649[M+H-2GluA-H_2_O-C_14_H_22_O]^+^;  235.1693[M+H-2GluA-H_2_O-C_15_H_22_O_2_]^+^ |  |  |  |  | √ |  |
| M121 | 16 | [M+COOH]^-^ | C_15_H_14_O_2_ | 226.0994 | 271.0979 | 5.32 | Isoliquiritin-Glu-2O+2H | 271.0979[M+COOH]^-^;  226.8909[M+COOH-HCOOH]^-^;  149.0193[M+COOH-HCOOH-C_6_H_4_]^-^;  105.0382[M+COOH-HCOOH-C_7_H_4_O_2_]^-^ |  |  |  | √ |  |  |
| M122 | 16.18 | [M+H]^+^ | C_30_H_46_O_6_ | 502.3294 | 503.3367 | 0.05 | Glycyrrhetinic acid+2O | 503.3356[M+H]^+^;  485.3257[M+H-H_2_O]^+^;  467.3165[M+H-2H_2_O]^+^;  403.3006[M+H-C_6_H_12_O]^+^;  333.2053[M+H-C_10_H_18_O_2_]^+^;  265.1437[M+H-C_15_H_26_O_2_]^+^ |  |  |  |  | √ |  |
| M123 | 16.26 | [M+H]^+^ | C_30_H_48_O_5_ | 488.3502 | 489.3572 | -0.54 | Glycyrrhetinic acid+O+2H | 489.3556[M+H]^+^  ;471.3481[M+H-H_2_O]^+^;  453.3360[M+H-2H_2_O]^+^;  435.3186[M+H-3H_2_O]^+^;  319.2252[M+H-C_10_H_20_O_2_]^+^;  233.1530[M+H-2H_2_O-C_15_H_24_O]^+^;  187.1493[M+H-2H_2_O-C_15_H_24_O-H_2_O-CO]^+^ |  |  |  |  | √ |  |
| M124 | 16.50 | [M+H]^+^ | C_43_H_64_O_17_ | 852.4148 | 853.4220 | 0.50 | Glycyrrhizic acid+O+CH_2_ | 853.4220[M+H]^+^;  439.3248[M+H-C_12_H_18_O_13_-CO_2_]^+^;  251.1712[M+H-C_12_H_18_O_12_-C_16_H_24_O_2_]^+^ |  |  |  |  | √ |  |
| M125 | 16.51 | [M-H]^-^ | C_42_H_64_O_15_ | 808.4245 | 807.4180 | 0.94 | Glycyrrhizic acid-GluA+Glu | 807.4224[M-H]^-^;  745.4241[M-H-CO_2_-H_2_O]^-^;  351.0582[M-H-C_30_H_46_O_3_]^-^;  193.0362[M-H-C_30_H_46_O_3_-C_6_H_8_O_5_]^-^;  113.0258[M-H-C_30_H_46_O_3_-C_6_H_8_O_5_-2H_2_O-CO_2_]^-^ |  |  |  |  | √ |  |
| M126 | 16.63 | [M+H]^+^ | C_38_H_56_O_11_ | 688.3823 | 689.3900 | 0.63 | Glycyrrhizic acid-GluA+C_2_H_2_O | 689.3887[M+H]^+^;  435.3271[M+H-C_8_H_12_O_8_-H_2_O]^+^;  285.2230[M+H-C_12_H_26_O_8_-H_2_O-CO]^+^;  217.1597[M+H-C_8_H_12_O_8_-C_14_H_22_-H_2_O-CO]^+^ |  |  |  |  | √ |  |
| M127 | 16.64 | [M+H]^+^ | C_44_H_64_O_17_ | 864.4144 | 865.4218 | 0.25 | Glycyrrhizic acid+C_2_H_2_O | 865.4042[M+H]^+^;  513.3580[M+H-C_8_H_12_O_7_-C_6_H_10_O_2_-H_2_O]^+^;  495.3460[M+H-C_24_H_34_O_3_]^+^;  477.3367[M+H-C_24_H_34_O_3_-H_2_O]^+^;  435.3264[M+H-C_14_H_20_O_14_-H_2_O]^+^;  417.3164[M+H-C_14_H_20_O_14_-2H_2_O]^+^ |  |  |  |  | √ |  |
| M128 | 17.74 | [M+H]^+^ | C_22_H_24_O_7_ | 400.1522 | 401.1593 | -0.39 | Liquiritin apioside-apiose-2O+CH_2_ | 401.1593[M+H]^+^;  167.0705[M+H-C_6_H_10_O_4_-C_7_H_4_]^+^;  151.0768[M+H-C_6_H_12_O_4_-C_8_H_6_]^+^;  123.0452[M+H-C_6_H_12_O_4_-C_9_H_6_O]^+^ |  |  |  |  | √ |  |
| M129 | 17.96 | [M+H]^+^ | C_36_H_54_O_11_ | 662.3652 | 663.3725 | -2.20 | Glycyrrhetinic acid+O+GluA | 663.3314[M+H]^+^;  645.3619[M+H-H_2_O]^+^;  617.3496[M+H-H_2_O-CO]^+^;  583.3705[M+H-H_2_O-CO-2HO]^+^;  487.3412[M+H-GluA]^+^;  469.3314[M+H-GluA-H_2_O]^+^;  451.3201[M+H-GluA-2H_2_O]^+^;  439.3177[M+H-C_6_H_10_O_6_-H_2_O-CO]^+^;  423.3266[M+H-GluA-2H_2_O-CO]^+^;  405.3159[M+H-GluA-3H_2_O-CO]^+^;  303.2010[M+H-GluA-2H_2_O-C_11_H_16_]^+^;  285.2248[M+H-GluA-3H_2_O-CO-C_9_H_12_]^+^;  251.1775[M+H-C_6_H_10_O_6_-C_15_H_22_O_2_]^+^;  233.1537[M+H-GluA-2H_2_O-C_15_H_23_O]^+^;  189.1632[M+H-GluA-2H_2_O-C_15_H_23_O-CO_2_]^+^ |  |  |  |  | √ |  |
| M130 | 18.03 | [M-H]^-^ | C_42_H_64_O_15_ | 808.4245 | 807.4176 | 0.42 | Glycyrrhizic acid-GluA+Glu | 807.4217[M-H]^-^;  745.4219[M-H-CO_2_-H_2_O]^-^;  351.0582[M-H-C_30_H_44_O_3_]^-^;  193.0362[M-H-C_30_H_44_O_3_-C_6_H_8_O_5_]^-^;  113.0261[M-H-C_30_H_44_O_3_-C_6_H_8_O_5_-2H_2_O-CO_2_]^-^ |  |  |  |  | √ |  |
| M131 | 18.17 | [M+H]^+^ | C_36_H_52_O_10_ | 644.3560 | 645.3628 | -0.86 | Glycyrrhizic acid-GluA-2H | 645.3637[M+H]^+^;  599.3552[M+H-H_2_O-CO]^+^;  451.3222[M+H]^+^;  405.3160[M+H-C_6_H_10_O_7_-CO_2_]^+^;  261.1497[M+H-C_30_H_32_O_7_]^+^ |  |  |  |  | √ |  |
| M132 | 18.37 | [M-H]^-^ | C_42_H_64_O_15_ | 808.4245 | 807.4168 | -0.51 | Glycyrrhizic acid-O+2H | 807.4152[M-H]^-^;  409.1862[M-H-C_12_H_17_O_12_-CHO_2_]^-^;  397.2209[M-H-GluA-C_15_H_22_O_2_]^-^;  233.1533[M-H-GluA-C_21_H_34_O_7_]^-^; 175.0232[M-H-C_30_H_36_O_3_-C_6_H_8_O_5_-H_2_O]^-^;  113.0243[M-H-C_30_H_36_O_3_-C_6_H_8_O_5_-2H_2_O-CO_2_]^-^ |  |  |  |  |  | √ |
| M133 | 18.43 | [M-H]^-^ | C_43_H_64_O_15_ | 820.4245 | 819.4169 | -0.41 | Glycyrrhizic acid-O+CH_2_ | 819.4106[M-H]^-^;  757.3805[M-H-CO_2_-H_2_O]^-^;  351.0571[M-H-C_31_H_48_O_3_]^-^;  113.0240[M-H-C_31_H_48_O_3_-C_6_H_8_O_5_-2H_2_O-CO_2_]^-^ |  |  |  |  | √ |  |
| M134 | 18.58 | [M+H]^+^ | C_36_H_52_O_11_ | 660.3510 | 661.3576 | -1.02 | Glycyrrhizic acid-GluA+O-2H | 661.3589[M+H]^+^;  485.3265[M+H-GluA]^+^;  439.3214[M+H-GluA-CO-H_2_O]^+^;  235.1689[M+H-C_6_H_10_O_7_-C_15_H_20_O_2_]^+^ | √ |  |  |  | √ |  |
| M135 | 18.62 | [M+H]^+^ | C_43_H_64_O_16_ | 836.4194 | 837.4259 | -0.92 | Glycyrrhizic acid+CH_2_ | 837.3865[M+H]^+^;  453.3372[M+H-C_12_H_19_O_13_-CH_3_]^+^;  435.3216[M+H-C_12_H_19_O_13_-CH_3_-H_2_O]^+^;  407.3314[M+H-C_12_H_19_O_13_-CH_3_-H_2_O-CO]^+^ |  |  |  |  | √ |  |
| M136 | 19.15 | [M-H]^-^ | C_30_H_46_O_5_ | 486.3345 | 485.3265 | -1.51 | Glycyrrhizic acid-2GluA+O | 485.3271[M-H]^-^;  441.3367[M-H-CO_2_]^-^;  423.3274[M-H-CO_2_-H_2_O]^-^;  385.2756[M-H-C_5_H_8_O_2_]^-^;  370.2524[M-H-C_5_H_8_O_2_-CH_3_]^-^;  115.0407[M-H-C_24_H_34_O_3_]^-^ |  |  |  |  | √ |  |
| M137 | 19.39 | [M-H]^-^ | C_21_H_20_O_6_ | 368.1260 | 367.1180 | -1.84 | Liquiritin apioside-apiose-3O-2H | 367.1199[M-H]^-^;  337.0726[M-H-CH_2_O]^-^;  309.0409[M-H-CH_2_O-C_2_H_4_]^-^;  297.0413[M-H-C_4_H_7_O]^-^;  281.0462[M-H-C_4_H_6_O_2_]^-^;  253.0513[M-H-C_6_H_10_O_2_]^-^;  237.0558[M-H-C_6_H_10_O_3_]^-^;  201.0199[M-H-C_6_H_3_O-C_3_H_7_O_2_]^-^;  161.0245[M-H-C_6_H_10_O_2_-C_6_H_4_O]^-^;  148.0177[M-H-C_6_H_10_O_2_-C_7_H_5_O]^-^ |  |  |  |  | √ |  |
| M138 | 19.39 | [M-H]^-^ | C_42_H_64_O_16_ | 824.4194 | 823.4125 | 0.39 | Glycyrrhizic acid+2H | 823.4162[M-H]^-^;  351.0569[M-H-C_30_H_44_O_3_]^-^;  193.0356[M-H-C_30_H_44_O_3_-C_6_H_10_O_5_]^-^;  113.0253[M-H-C_30_H_44_O_3_-C_6_H_10_O_6_-H_2_O-CHO_2_-HO]^-^ |  |  |  |  | √ |  |
| M139 | 19.63 | [M-H]^-^ | C_42_H_60_O_15_ | 804.3932 | 803.3867 | 0.93 | Glycyrrhizic acid-O-2H | 803.3915[M-H]^-^;  627.3594[M-H-C_6_H_6_O_5_]^-^;  351.0572[M-H-C_30_H_44_O_3_]^-^ |  |  |  |  | √ |  |
| M140 | 19.78 | [M+H]^+^ | C_43_H_64_O_16_ | 836.4194 | 837.4260 | -0.84 | Glycyrrhizic acid+CH_2_ | 837.4226[M+H]^+^;  453.3370[M+H-C_12_H_19_O_13_-CH_3_]^+^;  435.3263[M+H-C_12_H_19_O_13_-CH_3_-H_2_O]^+^;  407.3317[M+H-C_12_H_19_O_13_-CH_3_-H_2_O-CO]^+^ |  |  |  |  | √ |  |
| M141 | 20.06 | [M-H]^-^ | C_42_H_64_O_14_ | 792.4296 | 791.4227 | 0.51 | Glycyrrhizic acid-2O+2H | 791.4270[M-H]^-^;  729.4303[M-H-CO_2_-H_2_O]^-^;  351.0572[M-H-C_29_H_44_O_3_]^-^;  193.0339[M-H-C_29_H_44_O_3_-C_7_H_10_O_4_]^-^;  113.0248[M-H-C_29_H_44_O_3_-C_7_H_10_O_4_-2H_2_O-CO_2_-]^-^ |  |  |  |  | √ |  |
| M142 | 20.21 | [M-H]^-^ | C_44_H_64_O_16_ | 848.4194 | 847.4122 | 0.06 | Glycyrrhizic acid-O+C_2_H_2_O | 847.4167[M-H]^-^;  785.4189[M-H-CO_2_-H_2_O]^-^;  455.2506[M-H-C_14_H_16_O_13_]^-^;  351.0565[M-H-C_30_H_45_O_3_-C_2_H_3_O]^-^;  193.0342[M-H-C_30_H_45_O_3_-C_2_H_3_O-C_6_H_8_O_5_]^-^;  113.0247[M-H-C_30_H_45_O_3_-C_2_H_3_O-C_6_H_8_O_5_-2H_2_O-CO_2_]^-^ |  |  |  |  | √ |  |
| M143 | 20.21 | [M-H]^-^ | C_42_H_62_O_15_ | 806.4089 | 805.4021 | 0.60 | Glycyrrhetinic acid-O+2GluA | 805.4043[M-H]^-^;  629.3720[M-H-GluA]^-^;  351.0570[M-H-C_30_H_46_O_3_]^-^;  193.0357[M-H-C_30_H_46_O_3_-C_6_H_8_O_5_]^-^;  113.0252[M-H-C_30_H_46_O_3_-C_6_H_8_O_5_-2H_2_O-CO_2_]^-^ |  |  |  |  | √ |  |
| M144 | 20.31 | [M+H]^+^ | C_36_H_54_O_9_ | 630.3768 | 631.3829 | -1.91 | Glycyrrhizic acid-GluA-O | 631.3680[M+H]^+^;  437.3417[M+H-GluA-H_2_O]^+^;  301.2211[M+H-C_6_H_10_O_5_-C_10_H_16_O_2_]^+^;  247.1663[M+H-C_6_H_10_O_5_-C_14_H_22_O_2_]^+^ |  |  |  |  | √ |  |
| M145 | 20.63 | [M-H]^-^ | C_22_H_24_O_7_ | 400.1522 | 399.1448 | -0.38 | Liquiritin apioside-apiose-2O+CH_2_+iso | 399.1295[M-H]^-^;  338.1159[M-H-CH_2_O-CH_3_O]^-^;  295.0615[M-H-2CH_3_O-C_2_H_2_O]^-^;  161.0233[M-H-C_15_H_10_O_3_]^-^;  149.0252[M-H-C_7_H_12_OO_3_-C_7_H_5_O]^-^;  109.0314[M-H-C_7_H_12_O_4_-C_9_H_6_O]^-^ |  |  |  |  | √ |  |
| M146 | 21.29 | [M+H]^+^ | C_36_H_54_O_10_ | 646.3717 | 647.3784 | -0.90 | Glycyrrhizic acid-GluA | 647.3797[M+H]^+^;  471.3478[M+H-GluA]^+^;  435.3257[M+H-GluA-2H_2_O]^+^;  407.3315[M+H-GluA-H_2_O-CO]^+^;  285.2218[M+H-GluA-H_2_O-CO-C_9_H_14_]^+^;  189.1650[M+H-GluA-H_2_O-C_15_H_23_O-H_2_O-CO]^+^ | √ |  | √ | √ | √ |  |
| M147 | 21.35 | [M+H]^+^ | C_36_H_54_O_8_ | 614.3819 | 615.3883 | -1.29 | Glycyrrhizic acid-GluA-2O | 615.3892[M+H]^+^;  407.3338[M+H-C_6_H_10_O_5_-H_2_O-CO]^+^;  285.0803[M+H-C_6_H_10_O_5_-H_2_O-CO-C_9_H_15_]^+^;  235.1727[M+H-C_6_H_10_O_5_-C_15_H_22_O]^+^;  189.1643[M+H-C_6_H_10_O_5_-H_2_O-CO-C_15_H_22_O]^+^ |  |  |  |  | √ |  |
| M148 | 21.35 | [M+H]^+^ | C_36_H_56_O_9_ | 632.3924 | 633.3993 | -0.61 | Glycyrrhizic acid-2GluA+Glu | 633.3968[M+H]^+^;  471.3473[M+H-C_6_H_10_O_5_]^+^;  407.3318[M+H-C_6_H_12_O_6_-H_2_O-CO]^+^;  317.2140[M+H-C_6_H_12_O_6_-C_10_H_16_]^+^;  235.1705[M+H-C_6_H_12_O_6_-C_15_H_22_O]^+^;  189.1650[M+H-C_6_H_12_O_6_-H_2_O-CO-C_15_H_22_O]^+^ |  |  |  |  | √ |  |
| M149 | 21.55 | [M+H]^+^ | C_30_H_48_O_4_ | 472.3553 | 473.3626 | 0.06 | Glycyrrhetinic acid+2H | 473.3612[M+H]^+^;  455.3524[M+H-H_2_O]^+^;  437.3403[M+H-2H_2_O]^+^;  409.3083[M+H-2H_2_O-CO]^+^;  319.2274[M+H-C_9_H_14_O_2_]^+^ |  |  |  |  | √ |  |
| M150 | 21.91 | [M+H]^+^ | C_30_H_46_O_5_ | 486.3345 | 487.3416 | -0.47 | Glycyrrhetinic acid+O | 487.3411[M+H]^+^;  451.3222[M+H-2H_2_O]^+^;  441.3380[M+H-H_2_O-CO]^+^;  423.3270[M+H-2H_2_O-CO]^+^;  405.3166[M+H-3H_2_O-CO]^+^;  317.2127[M+H-2H_2_O-C_10_H_14_]^+^;  271.2068[M+H-2H_2_O-C_10_H_14_-H_2_O-CO]^+^;  235.1701[M+H-2H_2_O-C_15_H_20_O]^+^;  189.1642[M+H-3H_2_O-C_15_H_20_O-CO]^+^ | √ |  |  |  | √ |  |
| M151 | 22.36 | [M+H]^+^ | C_30_H_44_O_5_ | 484.3189 | 485.3258 | -0.81 | Glycyrrhetinic acid+O-2H | 485.3254[M+H]^+^;  467.3155[M+H-H_2_O]^+^;  449.3056[M+H-2H_2_O]^+^;  439.3209[M+H-H_2_O-CO]^+^;  421.3109[M+H-2H_2_O-CO]^+^;  403.3009[M+H-3H_2_O-CO]^+^;  317.2125[M+H-2H_2_O-C_10_H_12_]^+^;  271.2073[M+H-3H_2_O-C_10_H_12_-CO]^+^;  235.1700[M+H-2H_2_O-C_15_H_18_O]^+^;  189.1638[M+H-2H_2_O-C_15_H_18_O-H_2_O-CO]^+^ | √ |  |  |  | √ |  |
| M152 | 22.51 | [M+H]^+^ | C_37_H_56_O_10_ | 660.3873 | 661.3951 | 0.78 | Glycyrrhetinic acid+CH_2_+GluA | 661.3969[M+H]^+^;  453.3347[M+H-C_6_H_10_O_7_-CH_2_]^+^;407.3306[M+H-C_6_H_10_O_7_-CH_2_-H_2_O-CO]^+^;  317.2114[M+H-C_6_H_10_O_7_-CH_2_-C_10_H_16_]^+^ |  |  |  |  | √ |  |
| M153 | 22.91 | [M+H]^+^ | C_30_H_44_O_4_ | 468.3240 | 469.3314 | 0.41 | Glycyrrhizic acid-2GluA-2H | 469.3317[M+H]^+^;  451.3106[M+H-H_2_O]^+^;  423.3106[M+H-H_2_O-CO]^+^;  355.2290[M+H-H_2_O-CO-C_6_H_8_]^+^;  329.2110[M+H-H_2_O-C_6_H_12_O-C_3_H_3_]^+^;  315.1972[M+H-H_2_O-CO-C_6_H_8_-CH_4_]^+^;  287.2016[M+H-H_2_O-CO-C_6_H_8_-CH_4_-C_2_H_4_]^+^;  261.1500[M+H-H_2_O-C_6_H_12_O-C_3_H_3_-C_5_H_7_]^+^;  233.1545[M+H-H_2_O-C_6_H_12_O-C_3_H_3_-C_5_H_7_-CO]^+^ |  |  |  |  | √ |  |
| M154 | 23.24 | [M+H]^+^ | C_32_H_48_O_5_ | 512.3502 | 513.3573 | -0.23 | Glycyrrhetinic acid+C_2_H_2_O | 513.3566[M+H]^+^;  495.3466[M+H-H_2_O]^+^;  477.3360[M+H-2H_2_O]^+^;  453.3372[M+H-C_2_H_4_O_2_]^+^;  435.3270[M+H-C_2_H_4_O_2_-H_2_O]^+^;  407.3317[M+H-C_2_H_4_O_2_-H_2_O-CO]^+^;  299.2017[M+H-C_2_H_4_O_2_-H_2_O-CO-C_8_H_13_]^+^;  189.1636[M+H-C_2_H_4_O_2_-C_15_H_22_O-H_2_O-CO]^+^ |  |  |  |  | √ |  |
| M155 | 23.69 | [M+H]^+^ | C_30_H_48_O_3_ | 456.3603 | 457.3674 | -0.45 | Glycyrrhetinic acid-O+2H | 457.3661[M+H]^+^;  439.3566[M+H-H_2_O]^+^;  421.3459[M+H-2H_2_O]^+^;  303.2326[M+H-C_11_H_22_]^+^ |  |  |  |  | √ |  |
| M156 | 23.73 | [M+H]^+^ | C_30_H_44_O_4_ | 468.3240 | 469.3311 | -0.23 | Glycyrrhetinic acid-2H | 469.3311[M+H]^+^;  451.3219[M+H-H_2_O]^+^;  423.3271[M+H-H_2_O-CO]^+^;  405.3156[M+H-2H_2_O-CO]^+^;  317.2130[M+H-H_2_O-C_10_H_14_]^+^;  271.2064[M+H-H_2_O-C_10_H_14_-H_2_O-CO]^+^;  235.1700[M+H-H_2_O-C_15_H_20_O]^+^;  189.1647[M+H-2H_2_O-C_15_H_20_O-CO]^+^ |  |  |  |  | √ |  |
| M157 | 23.75 | [M+H]^+^ | C_30_H_44_O_3_ | 452.3290 | 453.3347 | -3.49 | Glycyrrhetinic acid-O-2H | 453.3363[M+H]^+^;  435.3264[M+H-H_2_O]^+^;  407.3308[M+H-H_2_O-CO]^+^;  351.2303[M+H-H_2_O-CO-C_4_H_7_]^+^;  299.1995[M+H-H_2_O-CO-C_8_H_13_]^+^ | √ |  |  |  |  |  |
| M158 | 24.05 | [M-H]^-^ | C_31_H_48_O_5_ | 500.3502 | 499.3418 | -2.20 | Glycyrrhetinic acid+O+CH_2_ | 499.3455[M+H]^+^;  439.6509[M-H-CO_2_-CH_4_]^-^;  395.2948[M-H-CO_2_-C_2_H_5_O_2_]^-^;  379.6500[M-H-CO_2_-C_2_H_5_O_2_-CH_3_]^-^ |  |  |  |  | √ |  |
| M159 | 24.08 | [M-H]^-^ | C_30_H_48_O_4_ | 472.3553 | 471.3468 | -2.52 | Glycyrrhetinic acid+2H | 471.3484[M-H]^-^;  453.3361[M-H-H_2_O]^-^;  427.3229[M-H-CO_2_]^-^;  407.3315[M-H-H_2_O-HCOOH]^-^ |  |  |  |  | √ |  |
| M160 | 24.32 | [M+H]^+^ | C_30_H_46_O_3_ | 454.3441 | 455.3514 | -1.30 | Glycyrrhetinic acid-O | 455.3158[M+H]^+^;  437.3417[M+H-H_2_O]^+^;  339.2672[M+H-C_6_H_12_O_2_]^+^；325.2564[M+H-H_2_O-CO-C_6_H_12_]^+^;  271.2050[M+H-C_10_H_17_-H_2_O-CO]^+^ |  |  |  |  |  | √ |
| M161 | 25.05 | [M+H]^+^ | C_30_H_46_O_2_ | 438.3498 | 439.3564 | -1.47 | Glycyrrhetinic acid-2O | 439.3274[M+H]^+^;  421.3458[M+H-H_2_O]^+^;  341.3083[M+H-CH_2_O-C_3_H_6_-CO]^+^;  245.1893[M+H-C_14_H_26_]^+^;  189.1633[M+H-CH_2_O-C_15_H_24_O]^+^ |  |  |  |  |  | √ |
| M162 | 25.21 | [M+H]^+^ | C_30_H_48_O_2_ | 440.3654 | 441.3720 | -1.66 | Glycyrrhetinic acid-2O+2H | 441.3501[M+H]^+^;  423.3591[M+H-H_2_O]^+^;  411.3519[M+H-C_2_H_6_]^+^;  341.2846[M+H-C_6_H_17_O]^+^;  287.2310[M+H-C_2_H_6_-C_9_H_16_]^+^;  257.2223[M+H-C_2_H_5_-C_9_H_16_-CH_3_O]^+^ |  |  |  |  |  | √ |

**Table S5** Typical metabolic reaction and the corresponding offsets of formula and mass.

| **Phase** | **Biotransformation reaction** | **Mass shift (Da)** | **Molecular**  **formula change** |
| --- | --- | --- | --- |
| Phase Ⅰ | Di-deglucuronidation | -352.0642 | -2GluA |
|  | Deglucuronidation | -176.0321 | -GluA (C_6_H_8_O_5_) |
|  | Deglycosylation | -162.0528 | -C_6_H_10_O_5_ |
|  | Loss of Apiose | -132.0423 | -Api (C_5_H_8_O_4_) |
|  | Deacetylation | -42.0106 | -C_2_H_2_O |
|  | Loss of CO | -27.9949 | -CO |
|  | Dehydration | -18.0106 | -H_2_O |
|  | Dehydroxylation | -15.9949 | -O |
|  | Dehydrogenation | -2.0141 | -2H |
|  | Parent | 0 | — |
|  | Isomerization | 0 | — |
|  | Dehydrogenation |  |  |
|  | Hydrogenation | 2.0157 | +2H |
|  | Hydroxylation | 15.9949 | +O |
|  | Hydrogenation+Methylation | 16.0298 | +2H+CH_2_ |
|  | Internal Hydrolysis (Hydration) | 18.0106 | +H_2_O |
|  | Dihydroxylation | 31.9898 | +2O |
| Phase Ⅱ | Methylation | 14.0157 | +CH_2_ |
|  | Hydroxylation+Methylation | 30.0106 | +O+CH_2_ |
|  | Acetylation | 42.0106 | +C_2_H_2_O |
|  | Methylation+Acetylation | 56.0263 | +CH_2_+C_2_H_2_O |
|  | Sulfate Conjugation | 79.9568 | +Sul (SO_3_) |
|  | Methylation+Sulfate Conjugation | 93.9725 | +CH_2_+SO_3_ |
|  | Hydroxylation+Sulfate Conjugation | 95.9517 | +O+SO_3_ |
|  | Glucose Conjugation | 162.0528 | +C_6_H_10_O_5_ |
|  | Glucuronidation | 176.0321 | +C_6_H_8_O_6_ |
|  | Hydrogenation+Glucuronidation | 178.0478 | +2H+GluA |
|  | Methylation+Glucuronidation | 190.0478 | +CH_2_+GluA |
|  | Hydroxylation+Glucuronidation | 192.0270 | +O+GluA |
|  | Sulfate Conjugation+Glucuronidation | 255.9889 | +C_6_H_8_O_6_+SO_3_ |
|  | Di-glucuronidation | 352.0642 | +2GluA |





**Figure S1** The chemical structures of 9 reference standards of ZGC.

**

**

**Figure S2** The EICs of 9 reference standards of ZGC by UPLC-Q-TOF-MS in positive (A) and negative ion mode (B).





**Figure S3.** The spectrum of liquiritin in negative ion mode.





**Figure S4.** The proposed fragmentation pathway of liquiritin in negative ion mode.





**Figure S5.** The spectrum of liquiritin apioside in negative ion mode.





**Figure S6.** The proposed fragmentation pathway of liquiritin apioside in negative ion mode.





**Figure S7** The spectrum of ononin in negative ion mode.





**Figure S8** The proposed fragmentation pathway of ononin in negative ion mode.





**Figure S9** The spectrum of isoliquiritin in negative ion mode.

**

**

**Figure S10** The proposed fragmentation pathway of isoliquiritin in negative ion mode.





**Figure S11** The spectrum of liquiritigenin in negative ion mode.





**Figure S12** The proposed fragmentation pathway of liquiritigenin in negative ion mode.





**Figure S13** The spectrum of isoliquiritigenin in negative ion mode.





**Figure S14** The proposed fragmentation pathway of isoliquiritigenin in negative ion mode.





**Figure S15** The spectrum of glycyrrhizic acid in negative ion mode.





**Figure S16** The proposed fragmentation pathway of glycyrrhizic acid in negative ion mode.





**Figure S17** The spectrum of glycyrrhizic acid ammonium salt in positive ion mode.





**Figure S18** The proposed fragmentation pathway of glycyrrhizic acid ammonium salt in positive ion mode.





**Figure S19** The spectrum of glycyrrhetinic acid in positive ion mode.





**Figure S20** The proposed fragmentation pathway of glycyrrhetinic acid in positive ion mode.


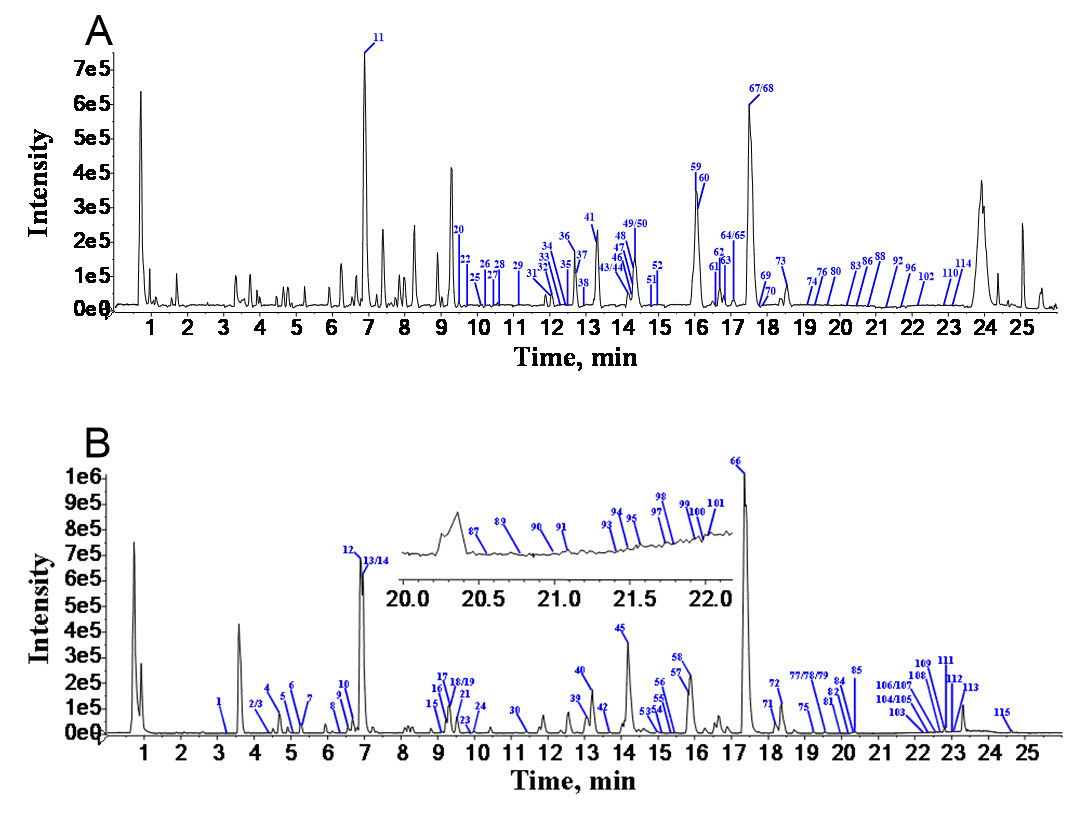


**Figure S21** The base peak chromatograms (BPCs) of the chemical compounds of ZGC aqueous extract by UPLC-Q-TOF-MS in positive ion mode (A) and in negative ion mode (B).





**Figure S22** The GO secondary classification histogram of the top 10 of biological process, cellular component and molecular function terms.





**Figure S23** The bubble of top 14 pathways of key targets from prototypes and phase Ⅰ metabolites against detoxification.
